# Novel Polyhydroquinoline-Hydrazide-Linked Schiff’s Base Derivatives: Multistep Synthesis, Antimicrobial, and Calcium-Channel-Blocking Activities

**DOI:** 10.3390/antibiotics11111568

**Published:** 2022-11-07

**Authors:** Zainab, Haitao Yu, Najeeb Ur Rehman, Mumtaz Ali, Aftab Alam, Abdul Latif, Nazish Shahab, Irfan Amir Khan, Abdul Jabbar Shah, Momin Khan, Ahmed Al-Ghafri, Ahmed Al-Harrasi, Manzoor Ahmad

**Affiliations:** 1 College of Chemistry and Materials Science, Hebei Normal University, Shijiazhuang 050024, China; 2 Department of Chemistry, University of Malakand, Chakdara 18800, Pakistan; 3 Natural & Medical Sciences Research Center, University of Nizwa, Nizwa 616, Oman; 4 State Key Laboratory of Chemical Resource Engineering, Beijing Engineering Center for Hierarchical Catalysis, Beijing Advanced Innovation Center for Soft Matter Science and Engineering, Beijing University of Chemical Technology, Beijing 100029, China; 5 Department of Pharmacy, COMSATS University Islamabad, Abbottabad Campus, Abbottabad 22060, Pakistan; 6 Institute of Pathology and Diagnostic Medicine, Khyber Medical University, Peshawar 25120, Pakistan

**Keywords:** polyhydroquinoline, Hantzsch derivatives, antibacterial, calcium-channel-blocking, spectroscopic data

## Abstract

Polyhydroquinoline (PHQ) are the unsymmetrical Hantzsch derivatives of 1,4-dihydropyridines with several biological applications. In this work, twenty-five (**3**–**27**) new Schiff’s base derivatives of polyhydroquinoline hydrazide were synthesized in excellent to good yields by a multi-component reaction. The structures of the synthesized products (**1**–**27**) were deduced with the help of spectroscopic techniques, such as ^1^H-, ^13^C -NMR, and HR-ESI-MS. The synthesized products (**1**–**27**) were tested for their antibacterial and in vitro calcium -channel-blocking (CCB) potentials using the agar-well diffusion method, and isolated rat aortic ring preparations, respectively. Among the series, sixteen compounds were found to inhibit the growth of *Escherichia coli* and *Enterococcus faecalis*. Among them, compound **17** was observed to be the most potent one at a dose 2 µg/mL, with an 18 mm zone of inhibition against both bacteria when it was compared with the standard drug amoxicillin. Eight compounds showed CCB activity of variable potency; in particular, compound **27** was more potent, with an EC_50_ value of 0.7 (0.3–1.1) µg/mL, indicating their CCB effect.

## 1. Introduction

Heterocycles containing polyhydroquinoline (PHQ) are asymmetric Hantzsch derivative of 1,4-dihydropyridines (1,4-DHPs) that are obtained by a four-component process [1]. They are a noticeable class of privileged compounds that are highly recognized in the field of medicines and organic chemistry [2,3,4,5] due to their pharmacological and biological activities. Some of the derivatives are well-known calcium- (Ca^2+^)-channel blockers [6] and have been reported to have anti-tubercular [7] bronchodilator properties [8], neuroprotection [9] anti-hyperglycemic [10], anti-diabetic [11,12], anti-inflammatory [13], platelet anti-aggregation [14], neuroprotective [15], anticancer [16], antibacterial [17], and antioxidant activities [18]. In addition, these compounds are used as calcium-channel blockers for the treatment of cardiovascular diseases, such as hypertension [19]. Previous studies have determined that PHQ derivatives can be utilized as anti-ischemics as well as, in the treatment of Alzheimer’s disease [20,21]. However, the importance of these bioactive molecules has inspired researchers to identify new similar substances with different structures and to better understand the relationship between the changes in the chemical structure and biological activities.

The rapid and continuous enhancement of drug-resistant bacterial infections has demanded a worldwide effort to search for new generation drugs [22,23,24]. Exploring non-toxic, effective, and safe chemotherapeutical gents is still a very important concern due to the growth of multi-resistant bacterial strains [25,26]. Infectious diseases continue to attack human beings with opportunistic infectious disorders, and infection with drug-resistant microorganisms [27,28]. During recent years, the treatment of bacterial infections has been challenging in patients with weak immune systems or with other associated diseases [29,30]. Natural and synthetic products are still one of the major sources of new drug molecules today, and synthetic products occupy a major part of the antimicrobial compounds that have been discovered to date. However, most of the commercially available antibiotics are correlated with side effects, such as nausea, dizziness, allergic reactions, and neurotoxicity [31]. Keeping in mind the side effects that are associated with many of the commercial antibiotics, it is highly recommended to discover a novel group of antibacterial agents with minimal side effects [32].

Calcium-channel blockers (CCBs) are a class of drug that are used to lower blood pressure by inhibiting the calcium movement from entering the cells of the heart and the arteries [33]. They are used in the management of angina pectoris, hypertension, subarachnoid hemorrhage, supraventricular arrhythmias, and pulmonary hypertension, and for the prevention of migraines [34]. As calcium causes heart and arteries to squeeze and contract more strongly by blocking calcium, while CCBs permit the blood vessels to relax, open, and slow down the heart rate, which can further lower the blood pressure. Since their introduction nearly two decades ago, CCBs have been shown to be effective medications in controlling blood pressure and anginal symptoms [35,36]. Furthermore, the Joint National Committee on Prevention, Detection, Evaluation, and Treatment of High Blood Pressure [37], as well as the European Society of Hypertension [38], recommend calcium-channel blockers of the dihydropyridines (nicardipine, nifedipine, amlodipine, felodipine, lacidipine, and isradipine), and the nondihydropyridines (verapamil and diltiazem) for hypertension prevention and management [39,40].

The dominance of the PHQ core unit in medicinal chemistry, from pharmacological and biological point o view, clearly demonstrates the extraordinary potential of new PHQ analogs as a source of valuable drug candidates [41]. In addition, the synthesis of PHQs has been extensively studied to improve the reaction conditions, to maximize the product yields, and to minimize the reaction time, as well as a precursor bias in order to obtain multi-functionalized PHQs [42]. However, to the best of our knowledge, the combination of polyhydroquinolines (PHQs) and hydrazide with different aliphatic/aromatic aldehydes for antibacterial and calcium-channel-blocking has never been investigated. Hence, based on the above facts, the objective of this study was to synthesize new polyhydroquinoline-linked Schiff’s base derivatives and to investigate the above-mentioned activities.

## 2. Results and Discussion

### 2.1. Chemistry

**Scheme 1:**
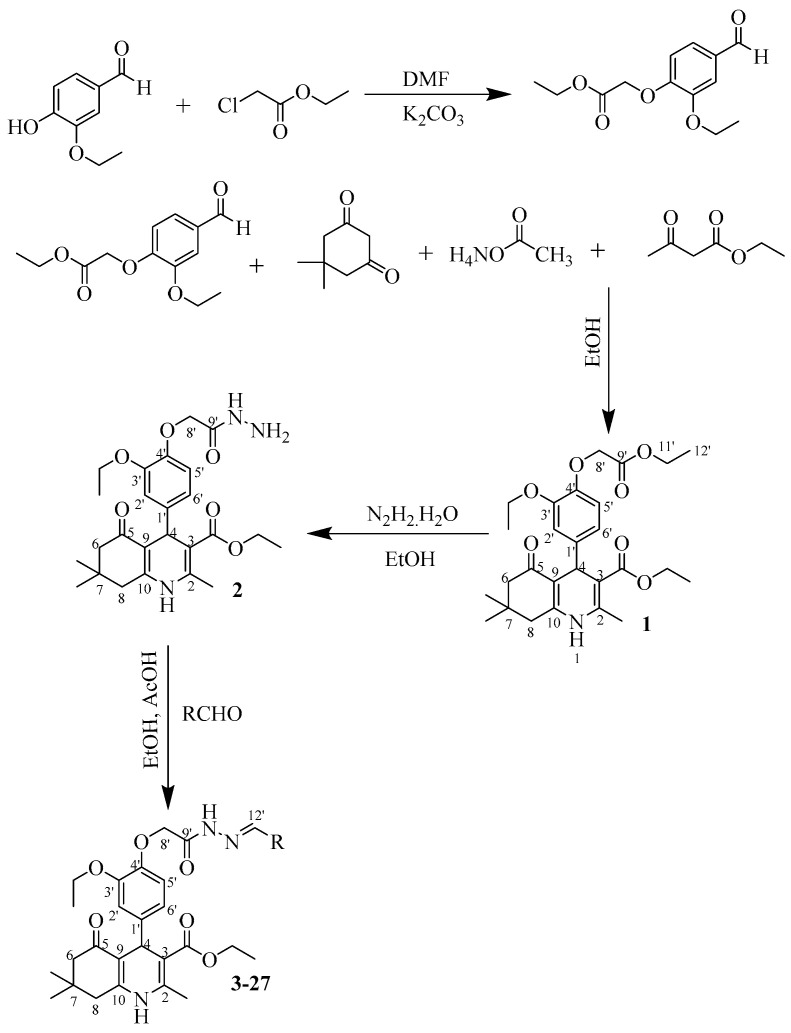
Synthesis of Schiff’s base derivatives based on polyhydroquinoline nucleus.

We have successfully synthesized twenty-five new biologically/pharmacologically active polyhydroquinoline Schiff’s base derivatives through multi-step reactions. In the first step, 3-ethoxy-4-hydroxybenzaldehyde was reacted with ethyl chloroacetate in the presence of potassium carbonate in dimethylformamide (DMF) solvent to produce esterified aldehyde. In the second step, the esterified aldehyde was reacted with dimedone, ethyl acetoacetate, and ammonium acetate in ethanol [43] to produce polyhydroquinoline (**1**). In the third step, a mixture of polyhydroquinoline and hydrazine hydrate was dissolved in ethanol to produce polyhydroquinoline hydrazide (**2**). Finally, polyhydroquinoline hydrazide (**2**) was further reacted with various substituted aliphatic/aromatic aldehydes in the presence of acid using ethanol solvent in order to produce the desired Schiff’s base derivatives of polyhydroquinoline **3**–**27** (Scheme 1, Table 1).

### 2.2. Antibacterial Bioassay

The antibacterial activities of the newly synthesized compounds (**1**–**27**) against *Escherichia coli* (gram-negative bacterium) and *Enterococcus faecalis* (gram-positive bacterium) were evaluated by measuring the zone of inhibition (ZOI) using the agar-well diffusion method [44,45]. The tested compounds were compared with the zone of inhibition that was produced by the amoxycillin. The results were recorded and interpreted accordingly (Table 1). Nine compounds (**17**, **21**, **22**, **1**, **16**, **8**, **2**, **26**, and **13**) displayed significant inhibition against *E. coli* and *E. faecalis*. Four compounds (**1**, **17**, **21**, and **22**) were found the most potent at a dose 2 µg/mL, with 18 mm ZOI against *E. coli* and *E. faecalis* when compared with the standard amoxicillin. Two compounds (**8** and **16**) displayed a good inhibition of 16 mm at a higher concentration (2 µg/mL) against *E. coli* and a promising inhibition of 18 mm in the case of *E. faecalis.* Similarly, compounds **2**, **26**, **13**, **3**, and **12** showed moderate to good inhibition, while compounds **9**, **18**, **5**, and **27** attributed non-significance inhibition (8–10 mm) (Table 2). Furthermore, compounds **4**, **6**, **7**, **10**, **11**, **14**, **15**, **19**, **20**, **23**, and **27** did not show any inhibition from the lower to the higher concentrations.

### 2.3. In Vitro Calcium-Channel-Blocking Study in Isolated Aorta from SD Rats

**Figure 1:**
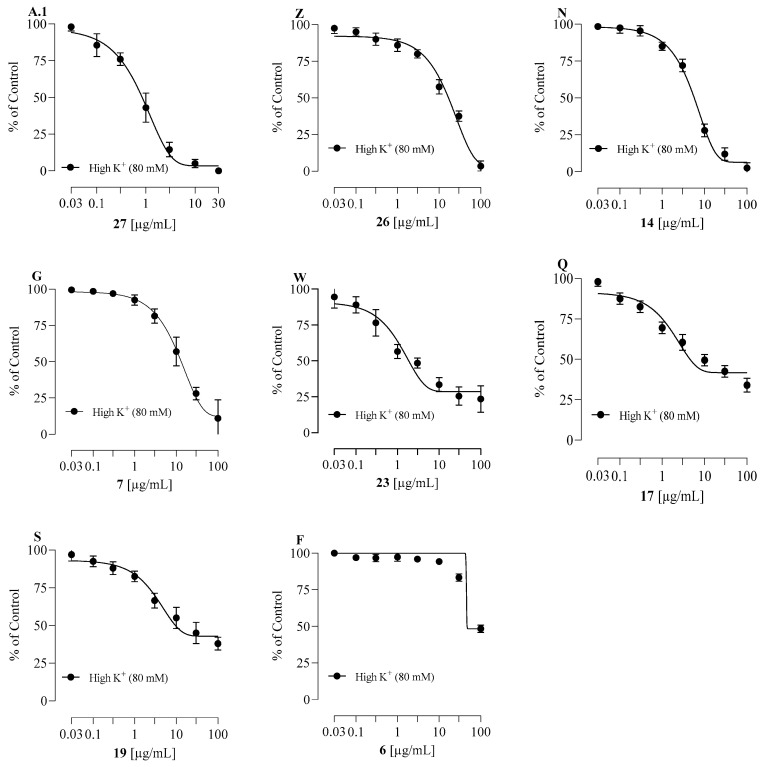
The effect of synthesized compounds **27** (A.1), **26** (Z), **14** (N), **7** (G), **23** (W), **17** (Q), **19** (S), and **6** (F) against high K^+^ (80 mM)-induced contraction (n = 3–5), values represented as mean ± SEM using two-way ANOVA.

An aortic ring was incubated in normal Kreb’s solutions and the cumulative addition of the tested compounds (**1**–**27**) determined a vasorelaxant response against high K^+^-induced contraction. The results have suggested that compound **27** was the most potent compound, with 100% vasorelaxation in the isolated aortic ring preparation, with an EC_50_ value of 0.7 (0.3–1.1) µg/mL (Figure 1 (A.1)). Similarly, compounds **26**, **14**, **7**, **23**, **17**, **19,** and **6** displayed a significant vasorelaxant response against high K^+^-induced contraction, with EC_50_ values of 14.8 (9.5–20.1), 5.18 (2.5–7.8), 12.5 (9.5–15.6), 1.6 (1.2–2.1), 3.8 (3.2–4.4), 9.6 (2.5–16.7), and 46.4 (29.5–63.3) respectively, indicating calcium channel antagonistic/blocking potential (Figure 1, Z, N, G, W, Q, S, F). Compounds **1**–**5**, **8**–**13**, **15**, **16**, **18**, **20**–**22**, **24**, and **25** displayed no significant vasorelaxant response against high K^+^-induced contraction, indicting poor calcium-channel-antagonistic/blocking potential (Figure S1).

In the isolated rat aortic ring preparation, the synthesized compounds were investigated against high K^+^ (80 mM)-induced contraction. The high K^+^ depolarizes the smooth muscle cell membrane and opens the voltage-dependent calcium channels (VDCCs), resulting in an influx of extracellular calcium (Ca^++^) and an activation of the contractile machinery [46]. Some of the compounds (**27**, **26**, **14**, **7**, **23**, **17**, **19**, and **6**) displayed a significant vasorelaxant response against high K^+^-induced contraction. It is well known that the contraction of the smooth muscles, such as rat aortic ring preparation, is dependent upon an increase in the cytoplasmic concentration of calcium (Ca^++^) ions for activating the contractile element [47]. The increase in intracellular Ca^++^ occurs either via influx through VDCCs or via its release from intracellular stores in the sarcoplasmic reticulum [48]. Some of these synthesized compounds caused the inhibition of high K^+^-induced contraction in the isolated rat aortic ring preparations, indicating their blocking effect on VDCCs. The findings from our current investigation provide the mechanistic pharmacological rationale of compounds **27**, **26**, **14**, **7**, **23**, **17**, **19**, and **6** for further detailed evaluation as antihypertensive agents, and compounds **27**, **26**, **14**, **7**, **23**, **17**, **19**, and **6** as antimicrobial agents.

## 3. Conclusions

Novel polyhydroquinoline derivatives (**1**–**27**) were synthesized in good to excellent yields using a standard procedure. All the synthesized derivatives were confirmed with the help of various spectroscopic techniques, such as HR-ESI-MS, ^1^H-NMR, and ^13^C-NMR, and, finally, were screened for their antimicrobial, and in vitro calcium-channel-blocking (CCB) potential using the agar-well diffusion method and isolated rat aortic ring preparations, respectively. Among the series, sixteen compounds were found to inhibit the growth of *Escherichia coli* and *Enterococcus faecalis.* Compound **17** was found to be the most potent at a dose of 2 µg/mL, with an 18 mm zone of inhibition against *E. coli* and *E. faecalis* when compared with the standard drug amoxicillin. Eight compounds showed CCB activity of variable potency; compound **27** was more potent, with an EC_50_ value of 0.7 (0.3–1.1) µg/mL, indicating a CCB effect. It can be concluded that due to the active potential of the synthesized derivatives, the medicinal chemists need to investigate these compounds in more detail in the field of medicinal chemistry.

## 4. Experimental

### 4.1. General

All of the chemicals used were analytical grade and were purchased from Sigma-Aldrich (St Louis, MO, USA) and used without further purification. Thin-layer chromatography (TLC) was performed on Merck Silica gel 60 F_254_ plates using the solvent system ethyl acetate/*n*-hexane. The melting points were recorded on a Stuart apparatus. Modern high-resolution electrospray ionization spectroscopy (HR-ESIMS) (Agilent 6530 LC Q-TOF, manufactured in USA/EU, made in Singapore) was used to confirm the masses of the synthesized compounds. ^1^H- and ^13^C-NMR spectra were recorded on a nuclear magnetic resonance (NMR) spectrometer (BRUKER, Zürich, Switzerland) spectrometer at 600 MHz and 150 MHz, respectively, using MeOD and CDCl_3_ solvents. The abbreviations that have been used in the work are as follows: s: singlet, d: doublet, t: triplet, m: multiplet and *J*: coupling constant to explain NMR signals in Hertz (Hz) and chemical shifts (δ). The values were expressed in parts per million (ppm). The structures of all compounds were confirmed with the help of HRESIMS and 1D (^1^H- and ^13^C) NMR spectroscopy.

### 4.2. Animals

The Sprague–Dawley rats (200–250 g) and mice (20–25 g) of either sex used in the study were housed in the animal house of the COMSATS University Islamabad Abbottabad campus in a controlled environment. The animals were given tap water ad libitum and a standard diet.

### 4.3. Synthesis of Ethyl-2-(2-ethoxy-4-formylphenoxy) Acetate

In a 100 mL round bottomed (RB) flask, 3-ethoxy-4-hydroxy benzaldehyde (3 g, 0.018 moles) was dissolved into 30 mL DMF solvent and potassium carbonate (K_2_CO_3_) was added to it. The reaction was continuously stirred for 30 min at 120 °C. After 30 min, ethyl chloroacetate (1.9 mL) was added to it and it was refluxed for 8–10 h. The product formation was checked with thin-layer chromatography (TLC) in a solvent system *n*-hexane and ethyl acetate (7:3). Upon completion, the reaction mixture was cooled to room temperature and poured into ice-cold distilled water. The precipitates that were formed were filtered, washed with an excess of water, dried under air, weighed, and recrystallized with ethanol to obtain the pure esterified product.

White amorphous powder was as follows: Yield: 92%; M.P: 55–58 °C; ^1^H-NMR (500 MHz, DMF): δ 9.82 (s, 1H, -CHO), 7.49–7.40 (m, 1H, Ar-H), 7.40 (s, 1H, Ar-H), 7.06 (d, *J* = 10 Hz, 1H, Ar-H), 4.93 (s, 2H, -OCH_2_CO), 4.19–4.08 (m, 4H, 2-OCH_2_CH_3_), 1.35 (t, *J* = 8.5 Hz, 3H, -OCH_2_CH_3_), and 1.20 (t, *J* = 9 Hz, 3H, -OCH_2_CH_3_). EI-MS (*m/z*: 252.1 [M^+^]).

### 4.4. Synthesis of Polyhydroquinoline (***1***)

Ethyl-2-(2-ethoxy-4-formylphenoxy)acetate Figure S2 (3.27 g) and dimedone (1.8 g) were dissolved into 100 mL RB flask, dissolved in 30 mL absolute ethanol, and stirred for 30 min. Then, ethyl acetoacetate (1.65 mL) and ammonium acetate (3.96 g) were added to the reaction mixture and refluxed for 6–7 h. The progress of the reaction was monitored by TLC using system *n*-hexane and ethyl acetate (7:3), respectively. After the reaction was complete, as indicated by TLC, the reaction mixture was poured into cold water. The resulting precipitate was filtered, washed with an excess of water and hot *n*-hexane, dried, and collected for further reaction. The obtained product was further confirmed by different spectroscopic techniques, such as NMR (^1^H-, ^13^C) and HR-ESI-MS.

### 4.5. Synthesis of Polyhydroquinoline Hydrazide (***2***)

Polyhydroquinoline **1** (5.01 g) and hydrazine hydrate (1.5 mL) were taken into a 100 mL RB flask in 15 mL ethanol solvent and refluxed for 4–5 h to produce the desired product (hydrazide) in good yield. The product formation was checked by TLC using a solvent system (*n*-hexane: ethyl acetate, 3:7). After the completion of the reaction, it was cooled to room temperature and poured into a beaker containing ice-cold distilled water. Precipitates were formed, which were filtered and washed with an excess of water to remove un-reacted hydrazine. The product was dried at room temperature and recrystallized from ethanol to obtain the desired compound in pure form. The formation of the product was further confirmed by mass and NMR.

### 4.6. General Procedure for the Synthesis of Schiff’s Base Derivatives of Polyhydroquinoline (***3***–***27***)

Twenty-five polyhydroquinoline-based Schiff’s base derivatives were synthesized from the desired hydrazide **2**. Various substituted aromatic/aliphatic aldehydes were reacted with hydrazide of polyhydroquinoline in 100 mL RB flask containing 15 mL ethanol solvent, a catalytic amount of glacial acetic acid was added, and it was stirred for 20–30 min (Table 1). The polyhydroquinoline hydrazide (120 mg) was then added to the reaction mixture and refluxed for 2–3 h. The progress of the reaction was monitored by TLC using a solvent system of hexane and ethyl acetate (6:4). After the completion of the reaction, the mixture was cooled to room temperature and poured into ice-cold distilled water. Precipitates were formed, filtered, washed with hot *n*-hexane to remove un-reacted aldehydes, and dried under air to obtain pure compounds **3**–**27**. In some cases, no precipitates were formed, so the reaction mixture was extracted with ethyl acetate to obtain pure products. The desired products were collected, weighed, and confirmed by different spectroscopic techniques, such as mass and NMR.

### 4.7. Spectral Interpretation of the Synthesized Compounds (Figure S3–S29)

Ethyl 4-(3-Ethoxy-4-(2-ethoxy-2-oxoethoxy)phenyl)-2,7,7-trimethyl-5-oxo-1,4,5,6,7,8-hexahydroquinoline-3-carboxylate (**1**)

Light yellow amorphous powder was as follows: Yield: 80%; M.P: 126–127 °C; ^1^H-NMR (600 MHz, CDCl_3_): *δ* 6.90 (s, 1H, Ar-H, H-2′), 6.67 (s, 2H, Ar-H, H-5′, H-6′), 4.96 (s, 1H, H-4), 4.58 (s, 2H, H-8′), 4.22–4.19 (m, 2H, OCH_2_CH_3_), 4.05–4.03 (m, 4H, OCH_2_CH_3_, CH_2_CH_3_), 2.37 (s, 3H, -CH_3_), 2.26–2.21 (m, 4H, H-6, H-8), 1.38 (t, *J* = 7.2 Hz, 3H, CH_2_CH_3_), 1.25 (t, *J* = 7.2 Hz, 3H, OCH_2_CH_3_), 1.17 (t, *J* = 7.2 Hz, 3H, OCH_2_CH_3_), 1.05 (s, 3H, CH_3_), and 0.90 (s, 3H, CH_3_). ^13^C-NMR (150 MHz, CDCl_3_) was as follows: *δ* 170.5 (-CO), 169.5 (-CO), 168.5 (-CO), 149.1 (C), 148.5 (C), 147.7 (C), 146.1 (C), 138.5 (C), 121.9 (C), 120.2 (CH), 115.4 (CH), 114.4 (CH), 104.2 (C), 67.1 (CH_2_), 64.9 (OCH_2_*CH_3_), 61.8 (OCH_2_*CH_3_), 61.1 (OCH_2_*CH_3_), 50.3 (CH_2_), 41.1 (CH_2_), 40.8 (CH), 32.8 (C), 27.2 (CH_3_), 27.1 (CH_3_), 19.1 (CH_3_), 14.8 (CH_2_CH_3_*), 14.2 (OCH_2_CH_3_*), and 14.1 (OCH_2_CH_3_*). LC-HRMS (ESI^+^) was as follows: Found [M − H]^+^: 484.23828; cald. for C_27_H_34_NO_7_: 484.23353.

Ethyl 4-(3-ethoxy-4-(2-(hydrazinyloxy)-2-oxoethoxy)phenyl)-2,7,7-trimethyl-5-oxo-1,4,5,6,7,8-hexahydroquinoline-3-carboxylate (**2**)

White amorphous powder was as follows: Yield: 79%; M.P: 204–205 °C; ^1^H-NMR (600 MHz, CDCl_3_): *δ* 10.26 (br.s, 1H, H-10′), 8.54 (s, 1H, H-1), 6.83 (s, 1H, Ar-H, H-2′), 6.70 (s, 2H, Ar-H, H-5′), 6.47 (s, 2H, Ar-H, H-6′), 4.83 (br.s, 1H, H-4), 4.50 (s, 2H, H-8′), 3.98–3.91 (m, 6H, H-11′, CH_2_CH_3_, OCH_2_CH_3_), 2.49 (br.s, 1H, H-6), 2.26 (s, 3H, CH_3_), 2.10–1.97 (m, 3H, H-6, H-8), 1.38 (t, *J* = 7.2 Hz, 3H, CH_2_CH_3_), 1.15 (t, *J* = 7.2 Hz, 3H, OCH_2_CH_3_), 1.05 (s, 3H, CH_3_), and 0.89 (s, 3H, CH_3_). ^13^C-NMR (150 MHz, CDCl_3_) was as follows: *δ* 194.9 (-CO), 167.9 (-CO), 167.2 (-CO), 152.8 (C), 149.3 (C), 148.0 (C), 145.0 (C), 143.2 (C), 119.6 (CH), 116.0 (CH), 113.4 (CH), 110.5 (C), 104.2 (C), 68.8 (CH_2_), 63.8 (CH_2_*CH_3_), 62.5 (OCH_2_*CH_3_), 50.4 (CH_2_), 40.1 (CH_2_), 35.6 (CH), 32.0 (C), 29.1 (2 × CH_3_), 18.4 (CH_3_), 14.5 (CH_2_CH_3_*), and 14.0 (OCH_2_CH_3_*). LC-HRMS (ESI^+^) was as follows: Found [M + H]^+^: 472.25445; cald. for C_25_H_34_N_3_O_6_: 472.24476.

Ethyl 4-(3-ethoxy-4-(2-(2-(4-nitrobenzylidene)hydrazinyl)-2-oxoethoxy)phenyl)-2,7,7-trimethyl-5-oxo-1,4,5,6,7,8-hexahydroquinoline-3-carboxylate (**3**)

Orange amorphous powder was as follows: Yield: 81%; M.P: 142–144 °C; ^1^H-NMR (600 MHz, CDCl_3_): *δ* 10.43 (s, 1H, H-10′), 8.69 (s, 1H, H-12′), 8.35 (s, 1H, H-1), 8.23 (d, *J* = 9.0 Hz, 2H, Ar-H, H-3″, H-5″), 7.90 (d, *J* = 9.0 Hz, 2H, Ar-H, H-2″, H-6″), 7.02 (br.s, 1H, Ar-H, H-2′), 6.79–6.74 (m, 2H, Ar-H, H-5′, H-6′), 4.99 (s, 1H, H-4), 4.63 (br.s, 2H, H-8′), 4.15–4.02 (m, 4H, OCH_2_CH_3_, CH_2_CH_3_), 2.35 (s, 3H, CH_3_), 2.35–2.20 (m, 2H, H-6), 2.22–2.15 (m, 2H, H-8), 1.45 (t, *J* = 7.2 Hz, 3H, OCH_2_CH_3_), 1.23 (s, 3H, CH_3_), 1.18 (t, *J* = 7.2 Hz, 3H, CH_2_CH_3_), and 1.05 (s, 3H, CH_3_). ^13^C-NMR (150 MHz, CDCl_3_) was as follows: *δ* 195.6 (-CO), 167.3 (-CO), 166.0 (-CO), 160.6 (C), 148.8 (C), 148.3 (C), 146.2 (CH), 145.6 (C), 143.3 (C), 139.7 (C), 129.4 (C), 128.3 (2 × CH), 124.1 (C), 124.0 (2 × -CH), 120.4 (CH), 116.8 (CH), 114.4 (CH), 105.8 (C), 70.5 (CH_2_), 64.6 (OCH_2_*CH_3_), 59.9 (CH_2_*CH_3_), 50.6 (CH_2_), 41.1 (CH_2_), 36.3 (CH), 32.7 (C), 29.7 (CH_3_), 27.2 (CH_3_), 19.5 (CH_3_), 15.1 (OCH_2_CH_3_*), and 14.1 (CH_2_CH_3_*). LC-HRMS (ESI^+^) was as follows: Found [M + H]^+^: 605.26020; cald. for C_32_H_37_N_4_O_8_: 605.26114.

Ethyl 4-(3-ethoxy-4-(2-(2-(3-nitrobenzylidene)hydrazinyl)-2-oxoethoxy)phenyl)-2,7,7-trimethyl-5-oxo-1,4,5,6,7,8-hexahydroquinoline-3-carboxylate (**4**)

Yellowish amorphous powder was as follows: Yield: 75%; M.P: 122–124 °C; ^1^H-NMR (600 MHz, CDCl_3_): *δ* 10.46 (s, 1H, H-10′), 8.47 (s, 1H, H-1), 8.37 (s, 1H, H-12′), 8.22 (d, *J* = 6.6 Hz, 1H, Ar-H, H-6″), 8.13 (d, *J* = 7.8 Hz, 1H, Ar-H, H-4″), 7.55 (t, *J* = 7.8 Hz, 1H, Ar-H, H-5″), 6.99 (s, 1H, Ar-H, H-2″), 6.77 (m, 2H, Ar-H, H-5′, H-6′), 6.52 (br.s, 1H, Ar-H, H-2′), 4.98 (s, 1H, H-4), 4.62 (s, 2H, H-8′), 4.06–4.02 (m, 4H, OCH_2_CH_3_, CH_2_CH_3_), 2.34 (s, 3H, CH_3_), 2.29–2.12 (m, 4H, H-6, H-8), 1.44 (t, *J* = 7.2 Hz, 3H, OCH_2_CH_3_), 1.19 (t, *J* = 7.2 Hz, 3H, CH_2_CH_3_), 1.03 (s, 3H, CH_3_), and 0.90 (s, 3H, CH_3_). ^13^C-NMR (150 MHz, CDCl_3_) was as follows: *δ* 195.6 (-CO), 167.4 (-CO), 166.0 (-CO), 148.6 (C), 148.4 (C), 146.5 (CH), 145.6 (C), 143.5 (C), 143.3 (C), 135.6 (C), 134.7 (C), 132.9 (CH), 129.8 (CH), 124.9 (CH), 122.6 (CH), 120.5 (CH), 116.7 (CH), 114.4 (CH), 111.8 (C), 106.0 (C), 70.4 (CH_2_), 64.6 (OCH_2_*CH_3_), 59.9 (CH_2_*CH_3_), 50.6 (CH_2_), 41.0 (CH_2_), 36.3 (CH), 32.7 (C), 29.4 (CH_3_), 27.2 (CH_3_), 19.4 (CH_3_), 15.1 (OCH_2_CH_3_*), and 14.3 (CH_2_CH_3_*). LC-HRMS (ESI^+^) was as follows: Found [M + H]^+^: 605.25955; cald. for C_32_H_37_N_4_O_8_: 605.26114.

Ethyl 4-(4-(2-(2-(2,4-dichlorobenzylidene)hydrazinyl)-2-oxoethoxy)-3-ethoxyphen yl)-2,7,7-trimethyl-5-oxo-1,4,5,6,7,8-hexahydroquinoline-3-carboxylate (**5**)

Light yellow amorphous powder was as follows: Yield: 69%; M.P: 120–121 °C; ^1^H-NMR (600 MHz, CDCl_3_): *δ* 10.50 (s, 1H, H-10′), 8.47 (s, 1H, H-1), 8.09 (d, *J* = 9.0 Hz, 1H, Ar-H, H-6″), 7.37 (s, 1H, H-12′), 7.23 (s, 1H, Ar-H, H-5″), 7.00 (s, 1H, Ar-H, H-3″), 6.78–6.74 (m, 2H, Ar-H, H-5′, H-6′), 6.36 (br.s, 1H, Ar-H, H-2′), 4.98 (s, 1H, H-4), 4.63 (s, 2H, H-8′), 4.13–4.08 (m, 4H, OCH_2_CH_3_, CH_2_CH_3_), 2.34 (s, 3H, CH_3_), 2.29–2.14 (m, 4H, H-6, H-8), 1.46 (t, *J* = 7.2 Hz, 3H, OCH_2_CH_3_), 1.17 (t, *J* = 7.2 Hz, 3H, CH_2_CH_3_), and 1.22 (s, 6H, 2 × -CH_3_). ^13^C-NMR (150 MHz, CDCl_3_) was as follows: *δ* 195.6 (-CO), 167.4 (-CO), 165.8 (-CO), 148.3 (C), 145.6 (C), 144.1 (CH), 143.6 (C), 143.3 (C), 136.9 (C), 134.8 (C), 129.6 (CH), 129.5 (2 × C), 128.8 (CH), 127.7 (CH), 120.5 (CH), 116.7 (CH), 114.2 (CH), 111.9 (C), 105.8 (C), 70.5 (CH_2_), 64.6 (OCH_2_*CH_3_), 59.9 (CH_2_*CH_3_), 50.6 (CH_2_), 41.0 (CH_2_), 36.3 (CH), 32.7 (C), 29.4 (CH_3_), 27.1 (CH_3_), 19.4 (CH_3_), 15.1 (OCH_2_CH_3_*), and 14.3 (CH_2_CH_3_*). LC-HRMS (ESI^+^) was as follows: Found [M + H]^+^: 628.20041; cald. for C_32_H_36_Cl_2_N_3_O_6_: 628.19812.

Ethyl 4-(3-ethoxy-4-(2-(2-(4-hydroxybenzylidene)hydrazinyl)-2-oxoethoxy)phen yl)-2,7,7-trimethyl-5-oxo-1,4,5,6,7,8-hexahydroquinoline-3-carboxylate (**6**)

Yellow amorphous powder was as follows: Yield: 71%; M.P: 142–144 °C; ^1^H-NMR (600 MHz, CDCl_3_): *δ* 10.13 (s, 1H, H-10′), 9.95 (s, 1H, H-1), 9.83 (s, 1H, H-OH), 8.03 (s, 1H, H-12′), 7.97 (s, 1H, Ar-H, H-2′), 7.77 (d, *J* = 8.4 Hz, 2H, Ar-H, H-3″, H-5″), 7.59 (d, *J* = 9.0 Hz, 2H, Ar-H, H-5′, H-6′), 7.53 (d, *J* = 8.4 Hz, 2H, Ar-H, H-2″, H-6″), 4.98 (s, 2H, H-8′), 4.86 (s, 1H, H-4), 4.65–4.58 (m, 2H, OCH_2_CH_3_, CH_2_CH_3_), 2.37 (s, 3H, CH_3_), 2.35–2.15 (m, 4H, H-6, H-8), 1.42 (t, *J* = 7.2 Hz, 6H, OCH_2_CH_3_, CH_2_CH_3_), 1.05 (s, 3H, CH_3_), and 0.89 (s, 3H, CH_3_). ^13^C-NMR (150 MHz, CDCl_3_) was as follows: *δ* 190.8 (-CO), 167.4 (-CO), 165.8 (-CO), 149.5 (CH), 148.9 (C), 148.2 (C), 145.1 (C), 143.2 (C), 132.3 (2 × C), 129.7 (2 × CH), 125.3 (C), 120.3 (CH), 116.0 (C-5′), 115.9 (2 × CH), 114.4 (CH), 112.0 (C), 106.0 (C), 69.4 (CH_2_), 64.6 (OCH_2_*CH_3_), 59.9 (CH_2_*CH_3_), 50.5 (C-6), 41.0 (CH_2_), 36.4 (CH), 32.8 (C), 29.3 (CH_3_), 27.2 (CH_3_), 19.4 (CH_3_), 14.9 (OCH_2_CH_3_*), and 14.3 (CH_2_CH_3_*). LC-HRMS (ESI^+^) was as follows: Found [M + H]^+^: 576.27357; cald. for C_32_H_38_N_3_O_7_: 576.27098.

Ethyl 4-(3-ethoxy-4-(2-(2-(3-hydroxybenzylidene)hydrazinyl)-2-oxoethoxy)phen yl)-2,7,7-trimethyl-5-oxo-1,4,5,6,7,8-hexahydroquinoline-3-carboxylate (**7**)

Yellow amorphous powder was as follows: Yield: 72%; M.P: 135–137 °C; ^1^H-NMR (600 MHz, CD_3_OD): *δ* 10.46 (s, 1H, H-10′), 8.19 (s, 1H, H-1), 7.87 (s, 1H, H-12′), 7.28 (br.s, 1H, Ar-H, H-2″), 7.27–7.22 (m, 1H, Ar-H, H-5″), 7.19–7.09 (m, 1H, Ar-H, H-6″), 7.00 (br.s, 1H, Ar-H, H-2′), 6.93 (d, *J* = 8.4 Hz, 1H, Ar-H, H-6′), 6.88–6.86 (m, 1H, Ar-H, H-4″), 6.82–6.76 (m, 1H, Ar-H, H-5′), 4.95 (s, 1H, H-4), 4.64 (s, 2H, H-8′), 4.15–4.05 (m, 4H, OCH_2_CH_3_, CH_2_CH_3_), 2.48 (d, *J* = 17.4 Hz, 1H, H-6), 2.36 (s, 3H, CH_3_), 2.35 (d, *J* = 16.8 Hz, 1H, H-6), 2.29 (d, *J* =16.2 Hz, 1H, H-8), 2.12 (d, *J* = 16.2 Hz, 1H, H-8), 1.43 (t, *J* = 7.2 Hz, 3H, OCH_2_CH_3_), 1.21 (t, *J* = 6.0 Hz, 3H, CH_2_CH_3_), 1.09 (s, 3H, CH_3_), and 0.93 (s, 3H, CH_3_). ^13^C-NMR (150 MHz, CD_3_OD) was as follows: *δ* 198.5 (-CO), 169.4 (-CO’), 168.5 (-CO), 159.0 (C), 152.6 (C), 151.2 (CH), 149.9 (C), 147.3 (C), 144.6 (C), 136.9 (C), 136.5 (C), 130.9 (CH), 120.9 (CH), 120.2 (CH), 118.0 (CH), 116.6 (CH), 115.5 (CH), 114.4 (CH), 112.1 (C), 106.5 (C), 70.8 (CH_2_), 68.3 (OCH_2_*CH_3_), 60.9 (CH_2_*CH_3_), 51.5 (CH_2_), 41.1 (CH_2_), 37.5 (CH), 32.8 (C), 29.7 (CH_3_), 27.1 (CH_3_), 18.6 (CH_3_), 15.3 (OCH_2_CH_3_*), and 14.7 (CH_2_CH_3_*). LC-HRMS (ESI^+^) was as follows: Found [M + H]^+^: 576.27434; cald. for C_32_H_38_N_3_O_7_: 576.27098.

Ethyl 4-(4-(2-(2-(3,5-dibromo-4-hydroxybenzylidene)hydrazinyl)-2-oxoethoxy)-3-ethoxyphenyl)-2,7,7-trimethyl-5-oxo-1,4,5,6,7,8-hexahydroquinoline-3-carboxylate (**8**)

Light yellow amorphous powder was as follows: Yield: 81%; M.P: 140–142 °C; ^1^H-NMR (600 MHz, CDCl_3_): *δ* 10.21 (s, 1H, H-10′), 8.01 (s, 1H, H-12′), 7.84 (s, 2H, Ar-H, H-2″, H-6″), 7.24 (s, 1H, H-1), 6.77–6.74 (m, 2H, Ar-H, H-5′, H-6′), 6.00 (s, 1H, Ar-H, H-2′), 4.98 (s, 1H, H-4), 4.61 (s, 2H, H-8′), 4.13–4.04 (m, 4H, OCH_2_CH_3_, CH_2_CH_3_), 2.35 (s, 3H, CH_3_), 2.29–2.13 (m, 4H, H-6, H-8), 1.43 (t, *J* = 7.2 Hz, 3H, OCH_2_CH_3_), 1.18 (t, *J* = 7.2 Hz, 3H, CH_2_CH_3_), 1.05 (s, 3H, CH_3_), and 0.92 (s, 3H, CH_3_). ^13^C-NMR (150 MHz, CDCl_3_) was as follows: *δ* 195.6 (-CO), 167.3 (-CO), 165.6 (-CO), 151.2 (C), 148.3 (C), 145.7 (CH), 145.6 (C), 143.3 (C), 143.2 (C), 136.5 (C), 131.1 (2 × CH), 128.5 (C), 120.4 (CH), 116.6 (CH), 114.4 (CH), 112.1 (C), 110.4 (2 × CH), 106.5 (C), 70.3 (CH_2_), 64.6 (OCH_2_*CH_3_), 59.9 (CH_2_*CH_3_), 50.6 (CH_2_), 41.1 (CH_2_), 36.3 (CH), 32.7 (C), 29.4 (CH_3_), 27.2 (CH_3_), 19.5 (CH_3_), 15.1 (OCH_2_CH_3_*), and 14.3 (CH_2_CH_3_*). LC-HRMS (ESI^+^) was as follows: Found [M + H]^+^: 734.09716; cald. for C_32_H_36_Br_2_N_3_O_7_: 734.08995.

Ethyl 4-(4-(2-(2-(2,4-dihydroxybenzylidene)hydrazinyl)-2-oxoethoxy)-3-ethoxy phenyl)-2,7,7-trimethyl-5-oxo-1,4,5,6,7,8-hexahydroquinoline-3-carboxylate (**9**)

Yellow amorphous powder was as follows: Yield: 74%; M.P: 143–145 °C; ^1^H-NMR (600 MHz, CD_3_OD): *δ* 10.36 (s, 1H, H-10′), 8.83 (s, 1H, H-12′), 8.34 (s, 1H, H-1), 7.21 (d, *J* = 8.4 Hz, 1H, Ar-H, H-6″), 6.99 (br.s, 1H, Ar-H, H-3″), 6.92 (d, *J* = 8.4 Hz, 1H, Ar-H, H-5″), 6.79–6.77 (m, 1H, Ar-H, H-5′), 6.39–6.38 (m, 1H, Ar-H, H-6′), 6.38 (br.s, 1H, Ar-H, H-2′), 4.97 (s, 1H, H-4), 4.59 (s, 2H, H-8′), 4.13–4.05 (m, 4H, OCH_2_CH_3_, CH_2_CH_3_), 2.48 (d, *J* = 17.4 Hz, 1H, H-6), 2.36 (s, 3H, CH_3_), 2.35 (d, *J* = 17.4 Hz, 1H, H-6), 2.29 (d, *J* = 16.2 Hz, 1H, H-8), 2.12 (d, *J* = 16.2 Hz, 1H, H-8), 1.43 (t, *J* = 6.6 Hz, 3H, OCH_2_CH_3_), 1.21 (t, *J* = 7.2 Hz, 3H, CH_2_CH_3_), 1.09 (s, 3H, CH_3_), and 0.93 (s, 3H, CH_3_). ^13^C-NMR (150 MHz, CD_3_OD) was as follows: *δ* 198.5 (-CO), 169.4 (-CO), 167.4 (-CO), 162.8 (C), 161.5 (C), 153.1 (CH), 152.6 (C), 149.9 (C), 147.3 (C), 146.2 (C), 144.6 (C), 133.5 (CH), 121.5 (CH), 118.0 (CH), 115.4 (CH), 112.1 (C), 111.6 (C), 108.9 (C), 106.5 (C), 103.9 (C), 70.8 (CH_2_), 65.7 (OCH_2_*CH_3_), 60.9 (CH_2_*CH_3_), 51.5 (CH_2_), 41.1 (CH_2_), 37.5 (CH), 33.5 (C), 29.7 (CH_3_), 27.1 (CH_3_), 18.6 (CH_3_), 15.3 (OCH_2_CH_3_*), and 14.7 (CH_2_CH_3_*). LC-HRMS (ESI^+^) was as follows: Found [M + H]^+^: 592.27153; cald. for C_32_H_37_N_3_O_8_: 592.26589.

Ethyl 4-(3-ethoxy-4-(2-(2-(2-hydroxy-3-methoxybenzylidene)hydrazinyl)-2-oxoe thoxy)phenyl)-2,7,7-trimethyl-5-oxo-1,4,5,6,7,8-hexahydroquinoline-3-carboxylate (**10**)

Light yellow amorphous powder was as follows: Yield: 68%; M.P: 125–127 °C; ^1^H-NMR (600 MHz, CDCl_3_): *δ* 11.09 (s, 1H, H-OH), 10.37 (s, 1H, H-10′), 9.89 (s, 1H, H-1), 8.51 (s, 1H, H-12′), 6.98 (s, 1H, Ar-H, H-2′), 6.95–6.81 (m, 3H, Ar-H, H-4″, H-5″, H-6″), 6.74 (br.s, 2H, Ar-H, H-5′, H-6′), 4.98 (s, 1H, H-4), 4.59 (s, 2H, H-8′), 4.06–4.03 (m, 4H, OCH_2_CH_3_, CH_2_CH_3_), 3.89 (s, 3H, OCH_3_), 2.36 (s, 3H, CH_3_), 2.24–2.17 (m, 4H, H-6, H-8), 1.43 (t, *J* = 7.2 Hz, 3H, OCH_2_CH_3_), 1.17 (t, *J* = 7.2 Hz, 3H, CH_2_CH_3_), 1.04 (s, 3H, CH_3_), and 0.91 (s, 3H, CH_3_). ^13^C-NMR (150 MHz, CDCl_3_) was as follows: *δ* 196.7 (-CO), 167.3 (-CO), 165.3 (-CO), 152.6 (C), 151.8 (CH), 148.3 (2 × C), 145.7 (C), 143.4 (C), 143.2 (C), 124.5 (C), 122.6 (CH), 120.4 (CH), 119.6 (CH), 119.1 (CH), 117.9 (C), 117.6 (C), 114.2 (CH), 114.1 (CH), 106.5 (C), 70.6 (CH_2_), 64.6 (OCH_2_*CH_3_), 59.9 (CH_2_*CH_3_), 56.2 (OCH_3_), 50.4 (CH_2_), 41.0 (CH_2_), 36.3 (CH), 32.7 (C), 29.3 (CH_3_), 27.2 (CH_3_), 19.4 (CH_3_), 15.0 (OCH_2_CH_3_*), and 14.0 (CH_2_CH_3_*). LC-HRMS (ESI^+^) was as follows: Found [M + H]^+^: 606.28084; cald. for C_33_H_40_N_3_O_8_: 606.28154.

Ethyl 4-(4-(2-(2-(2,4-dimethoxybenzylidene)hydrazinyl)-2-oxoethoxy)-3-ethoxy phenyl)-2,7,7-trimethyl-5-oxo-1,4,5,6,7,8-hexahydroquinoline-3-carboxylate (**11**)

Yellow amorphous powder was as follows: Yield: 82%; M.P: 112–114 °C; ^1^H-NMR (600 MHz, CDCl_3_): *δ* 10.49 (s, 1H, H-10′), 8.51 (s, 1H, H-12′), 7.96 (d, *J* = 8.4 Hz, 1H, Ar-H, H-6″), 7.24 (s, 1H, H-1), 6.99 (s, 1H, Ar-H, H-2′), 6.69 (br.s, 2H, Ar-H, H-5′, H-6′), 6.50 (d, *J* = 8.4 Hz, 1H, Ar-H, H-5″), 6.42 (br.s, 1H, Ar-H, H-3″), 4.98 (s, 1H, H-4), 4.59 (s, 2H, H-8′), 4.11–4.02 (m, 4H, OCH_2_CH_3_, CH_2_CH_3_), 3.86 (s, 3H, OCH_3_), 3.81 (s, 3H, OCH_3_), 2.39 (s, 3H, CH_3_), 2.37–2.21 (m, 4H, H-6, H-8), 1.45 (t, *J* = 7.6 Hz, 3H, OCH_2_CH_3_), 1.17 (t, *J* = 7.2 Hz, 3H, CH_2_CH_3_), 1.04 (s, 3H, CH_3_), and 0.91 (s, 3H, CH_3_). ^13^C-NMR (150 MHz, CDCl_3_) was as follows: *δ* 195.1 (-CO), 167.2 (-CO), 165.4 (-CO), 163.6 (C), 148.4 (C), 145.6 (CH), 144.2 (C), 143.5 (C), 142.7 (C), 140.3 (C), 136.9 (C), 130.8 (CH), 120.1 (CH), 116.6 (CH), 114.0 (CH), 115.0 (C), 111.4 (C), 106.0 (CH), 105.7 (CH), 104.0 (C), 70.1 (CH_2_), 64.4 (OCH_2_*CH_3_), 59.9 (CH_2_*CH_3_), 55.8 (OCH_3_), 55.6 (OCH_3_), 50.6 (CH_2_), 40.9 (CH_2_), 36.2 (CH), 32.8 (C), 29.1 (CH_3_), 27.3 (CH_3_), 19.2 (CH_3_), 14.9 (OCH_2_CH_3_*), and 14.3 (CH_2_CH_3_*). LC-HRMS (ESI^+^) was as follows: Found [M + H]^+^: 620.27785; cald. for C_34_H_42_N_3_O_8_: 620.29719.

Ethyl 4-(4-(2-(2-(4-(diethylamino)benzylidene)hydrazinyl)-2-oxoethoxy)-3-ethoxy phenyl)-2,7,7-trimethyl-5-oxo-1,4,5,6,7,8-hexahydroquinoline-3-carboxylate (**12**)

Orange amorphous powder was as follows: Yield: 70%; M.P: 104–105 °C; ^1^H-NMR (600 MHz, CDCl_3_): *δ* 9.91 (s, 1H, H-10′), 7.93 (s, 1H, H-1), 7.69 (d, *J* = 8.4 Hz, 1H, Ar-H, H-5′), 7.56 (br.s, 2H, Ar-H, H-2″, H-6″), 7.00 (s, 1H, H-12′), 6.75 (s, 2H, Ar-H, H-3″, H-5″), 6.66 (d, *J* = 9.0 Hz, 1H, Ar-H, H-6′), 6.58 (s, 1H, Ar-H, H-2′), 4.99 (s, 1H, H-4), 4.59 (s, 2H, H-8′), 4.12–4.02 (m, 4H, OCH_2_CH_3_, CH_2_CH_3_), 3.36–3.35 (m, 4H, 2 NCH_2_CH_3_), 2.35 (s, 3H, CH_3_), 2.34–2.11 (m, 4H, H-6, H-8), 1.43 (t, *J* = 7.2 Hz, 3H, OCH_2_CH_3_), 1.20 (t, *J* = 7.2 Hz, 3H, CH_2_CH_3_), 1.16 (t, 6H, 2NCH_2_CH_3_), 1.02 (s, 3H, CH_3_), and 0.90 (s, 3H, CH_3_). ^13^C-NMR (150 MHz, CDCl_3_) was as follows: *δ* 195.7 (-CO), 167.5 (-CO), 165.5 (-CO), 152.3 (C), 149.9 (C), 148.4 (C), 148.3 (C), 145.6 (C), 143.2 (CH), 132.5 (C), 129.7 (2×CH), 120.2 (CH), 116.4 (CH), 114.1 (CH), 124.5 (C), 111.9 (C), 111.0 (CH), 110.6 (CH), 105.6 (C), 70.2 (CH_2_), 64.4 (OCH_2_*CH_3_), 59.8 (CH_2_*CH_3_), 50.7 (CH_2_), 44.7 (NCH_2_*CH_3_), 44.4 (NCH_2_*CH_3_), 40.9 (CH_2_), 36.2 (CH), 32.7 (C), 29.4 (CH_3_), 27.2 (CH_3_), 19.3 (CH_3_), 15.1 (OCH_2_CH_3_*), 14.3 (CH_2_CH_3_*), and 12.5 (2NCH_2_CH_3_*). LC-HRMS (ESI^+^) was as follows: Found [M + H]^+^: 631.35680; cald. for C_36_H_46_N_4_O_6_: 631.34956.

Ethyl 4-(4-(2-(2-(4-bromo-2-fluorobenzylidene)hydrazinyl)-2-oxoethoxy)-3-ethoxy phenyl)-2,7,7-trimethyl-5-oxo-1,4,5,6,7,8-hexahydroquinoline-3-carboxylate (**13**)

Light yellow amorphous powder was as follows: Yield: 67%; M.P: 90–91 °C; ^1^H-NMR (600 MHz, CD_3_OD): *δ* 10.09 (s, 1H, H-10′), 8.50 (s, 1H, H-1), 8.13 (s, 1H, H-12′), 8.07 (t, *J* = 8.4 Hz, 1H, Ar-H, H-3″), 7.46–7.40 (m, 2H, Ar-H, H-5″, H-6″), 7.00 (br.s, 1H, Ar-H, H-2′), 6.92 (d, *J* = 7.8 Hz, 1H, Ar-H, H-5′), 6.82–6.76 (m, 1H, Ar-H, H-6′), 4.94 (s, 1H, H-4), 4.65 (s, 2H, H-8′), 4.14–4.05 (m, 4H, OCH_2_CH_3_, CH_2_CH_3_), 2.45 (d, *J* = 16.2 Hz, 1H, H-6), 2.36 (s, 3H, CH_3_), 2.29–2.12 (m, 4H, H-6, H-8), 1.42 (t, *J* = 7.2 Hz, 3H, OCH_2_CH_3_), 1.24 (t, *J* = 7.2 Hz, 3H, CH_2_CH_3_), 1.09 (s, 3H, CH_3_), and 0.95 (s, 3H, CH_3_). ^13^C-NMR (150 MHz, CD_3_OD) was as follows: *δ* 198.5 (-CO), 172.6 (-CO), 168.8 (-CO), 161.8 (C), 152.5 (C), 149.8 (C), 147.9 (C), 146.0 (C), 142.7 (CH), 138.2 (CH), 129.3 (CH), 128.9 (C), 126.0 (C), 121.3 (CH), 120.5 (CH), 117.9 (C), 116.6 (CH), 115.5 (CH), 112.1 (C), 106.5 (C), 70.7 (CH_2_), 68.3 (OCH_2_*CH_3_), 65.7 (CH_2_*CH_3_), 51.0 (CH_2_), 41.1 (CH_2_), 37.5 (CH), 33.5 (C), 29.7 (CH_3_), 27.1 (CH_3_), 18.6 (CH_3_), 15.3 (OCH_2_CH_3_*), and 14.7 (CH_2_CH_3_*). LC-HRMS (ESI^+^) was as follows: Found [M + H]^+^: 658.18143; cald. for C_32_H_36_BrFN_3_O_6_: 658.17511.

Ethyl 4-(3-ethoxy-4-(2-oxo-2-(2-(3,4,5-trimethoxybenzylidene)hydrazinyl)ethoxy) phenyl)-2,7,7-trimethyl-5-oxo-1,4,5,6,7,8-hexahydroquinoline-3-carboxylate (**14**)

Yellow amorphous powder was as follows: Yield: 66%; M.P: 121–122 °C; ^1^H-NMR (600 MHz, CDCl_3_): *δ* 10.10 (s, 1H, H-10′), 8.09 (s, 1H, H-1), 7.00 (s, 1H, H-12′), 6.95 (s, 2H, Ar-H, H-2″, H-6″), 6.77–6.72 (m, 2H, Ar-H, H-5′, H-6′), 6.20 (br.s, 1H, Ar-H, H-2′), 4.98 (s, 1H, H-4), 4.61 (s, 2H, H-8′), 4.11–4.03 (m, 4H, OCH_2_CH_3_, CH_2_CH_3_), 3.87 (s, 9H, OCH_3_), 2.36 (s, 3H, H-14), 2.33–2.17 (m, 4H, H-6, H-8), 1.44 (t, *J* = 6.6 Hz, 3H, OCH_2_CH_3_), 1.18 (t, *J* = 7.2 Hz, 3H, CH_2_CH_3_), 1.04 (s, 3H, CH_3_), and 0.91 (s, 3H, CH_3_). ^13^C-NMR (150 MHz, CDCl_3_) was as follows: *δ* 191.1 (-CO), 167.4 (-CO), 165.5 (-CO), 153.7 (C), 153.5 (2 × C), 149.4 (CH), 148.4 (C), 148.3 (C), 145.6 (C), 140.4 (C), 136.9 (C), 128.8 (C), 120.3 (CH), 114.4 (CH), 106.7 (CH), 105.0 (2 × CH), 111.9 (C), 106.7 (C), 70.1 (CH_2_), 64.6 (OCH_2_*CH_3_), 60.9 (CH_2_*CH_3_), 60.0 (OCH_3_), 56.3 (2 × OCH_3_), 50.7 (CH_2_), 41.1 (CH_2_), 36.3 (CH), 32.8 (C), 29.2 (CH_3_), 27.3 (CH_3_), 19.3 (CH_3_), 15.0 (OCH_2_CH_3_*), and 14.0 (CH_2_CH_3_*). LC-HRMS (ESI^+^) was as follows: Found [M + H]^+^: 650.31818; cald. for C_35_H_44_N_3_O_7_: 650.30776.

Ethyl 4-(3-ethoxy-4-(2-(2-((2-hydroxynaphthalen-1-yl)methylene)hydrazinyl)-2-oxoethoxy)phenyl)-2,7,7-trimethyl-5-oxo-1,4,5,6,7,8-hexahydroquinoline-3-carboxylate (**15**)

Dark yellow amorphous powder was as follows: Yield: 81%; M.P: 105–107 °C; ^1^H-NMR (600 MHz, CDCl_3_): *δ* 13.14 (s, 1H, H-OH), 10.80 (s, 1H, H-10′), 9.31 (s, 1H, H-1), 8.34 (d, *J* = 8.4 Hz, 1H, Ar-H, H-6″), 7.94 (d, *J* = 9.2 Hz, 1H, Ar-H, H-3″), 7.49 (t, *J* = 7.2 Hz, 1H, Ar-H, H-5″), 7.43 (t, *J* = 7.2 Hz, 1H, Ar-H, H-4″), 7.19 (d, *J* = 9.0 Hz, 1H, Ar-H, H-8″), 7.13 (d, *J* = 9.0 Hz, 1H, Ar-H, H-7″), 7.01 (s, 1H, H-12′), 6.79–6.77 (m, 2H, Ar-H, H-5′, H-6′), 6.40 (br.s, 1H, Ar-H, H-2′), 5.00 (s, 1H, H-4), 4.63 (s, 2H, H-8′), 4.14–4.03 (m, 4H, OCH_2_CH_3_, CH_2_CH_3_), 2.36 (s, 3H, CH_3_), 2.35–2.11 (m, 4H, H-6, H-8), 1.46 (t, *J* = 7.2 Hz, 3H, OCH_2_CH_3_), 1.17 (t, *J* = 7.2 Hz, 3H, CH_2_CH_3_), 1.04 (s, 3H, CH_3_), and 0.91 (s, 3H, CH_3_). ^13^C-NMR (150 MHz, CDCl_3_) was as follows: *δ* 195.6 (-CO), 167.3 (-CO), 165.0 (-CO), 159.3 (C), 148.5 (C), 148.3 (CH), 145.7 (C), 143.4 (C), 143.3 (C), 132.9 (CH), 132.1 (C), 129.5 (C), 129.2 (CH), 127.8 (C), 127.6 (CH), 124.5 (CH), 123.5 (CH), 120.5 (CH), 119.7 (CH), 117.0 (CH), 114.3 (CH), 111.8 (C), 111.3 (C), 107.8 (C), 70.6 (CH_2_), 64.6 (OCH_2_*CH_3_), 59.9 (CH_2_*CH_3_), 50.5 (CH_2_), 41.0 (CH_2_), 36.3 (CH), 32.7 (C), 29.4 (CH_3_), 27.2 (CH_3_), 19.4 (CH_3_), 15.1 (OCH_2_CH_3_*), and 14.3 (CH_2_CH_3_*). LC-HRMS (ESI^+^) was as follows: Found [M + H]^+^: 626.29339; cald. for C_36_H_40_N_3_O_7_: 626.28663.

Ethyl 4-(3-ethoxy-4-(2-(2-(2-methoxybenzylidene)hydrazinyl)-2-oxoethoxy) phenyl)-2,7,7-trimethyl-5-oxo-1,4,5,6,7,8-hexahydroquinoline-3-carboxylate (**16**)

Yellow amorphous powder was as follows: Yield: 65%; M.P: 114–116 °C; ^1^H-NMR (600 MHz, CDCl_3_): *δ* 10.31 (s, 1H, H-10′), 8.52 (s, 1H, H-12′), 8.01 (d, *J* = 7.2 Hz, 1H, Ar-H, H-6″), 7.34 (t, *J* = 7.2 Hz, 1H, Ar-H, H-4″), 7.00 (br.s, 2H, H-1, H-2′), 6.91 (t, *J* = 7.2 Hz, 1H, Ar-H, H-5″), 6.88 (d, *J* = 8.4 Hz, 1H, Ar-H, H-3″), 6.77–6.72 (m, 2H, Ar-H, H-5′, H-6′), 4.99 (s, 1H, H-4), 4.62 (s, 2H, H-8′), 4.15–4.02 (m, 4H, OCH_2_CH_3_, CH_2_CH_3_), 3.84 (s, 3H, OCH_3_), 2.37 (s, 3H, CH_3_), 2.31–2.19 (m, 4H, H-6, H-8), 1.47 (t, *J* = 7.2 Hz, 3H, OCH_2_CH_3_), 1.17 (t, *J* = 7.2 Hz, 3H, CH_2_CH_3_), 1.03 (s, 3H, CH_3_), and 0.90 (s, 3H, CH_3_). ^13^C-NMR (150 MHz, CDCl_3_) was as follows: *δ* 195.3 (-CO), 167.3 (-CO), 165.5 (-CO), 158.2 (C), 148.4 (C), 145.7 (C), 145.2 (CH), 143.6 (C), 143.0 (C), 136.0 (C), 132.2 (CH), 127.1 (CH), 120.9 (CH), 120.3 (CH), 116.7 (C), 114.0 (CH), 111.6 (2 × CH), 110.9 (CH), 106.1 (C), 70.5 (CH_2_), 64.5 (OCH_2_*CH_3_), 59.9 (CH_2_*CH_3_), 55.8 (OCH_3_), 50.1 (CH_2_), 41.0 (CH_2_), 36.3 (CH), 32.7 (C), 29.3 (CH_3_), 27.2 (CH_3_), 19.2 (CH_3_), 14.9 (OCH_2_CH_3_*), and 14.3 (CH_2_CH_3_*). LC-HRMS (ESI^+^) was as follows: Found [M + H]^+^: 590.28288; cald. for C_33_H_39_N_3_O_7_: 590.28663.

Ethyl 4-(4-(2-(2-(2-chlorobenzylidene)hydrazinyl)-2-oxoethoxy)-3-ethoxyphen yl)-2,7,7-trimethyl-5-oxo-1,4,5,6,7,8-hexahydroquinoline-3-carboxylate (**17**)

Light yellow amorphous powder was as follows: Yield: 73%; M.P: 120–121 °C; ^1^H-NMR (600 MHz, CDCl_3_): *δ* 10.42 (s, 1H, H-10′), 8.53 (s, 1H, H-12′), 8.16–8.14 (m, 1H, Ar-H, H-4″), 7.36 (d, *J* = 7.8 Hz, 1H, Ar-H, H-6″), 7.32–7.29 (m, 1H, Ar-H, H-5″), 7.27 (d, *J* = 7.8 Hz, 1H, Ar-H, H-3″), 7.24 (s, 1H, H-1), 7.01 (br.s, 1H, Ar-H, H-2′), 6.80 (d, *J* = 8.4 Hz, 1H, Ar-H, H-5′), 6.77 (d, *J* = 8.4 Hz, 1H, Ar-H, H-6′), 4.99 (s, 1H, H-4), 4.64 (s, 2H, H-8′), 4.16–4.02 (m, 4H, OCH_2_CH_3_, CH_2_CH_3_), 2.36 (s, 3H, CH_3_), 2.33–2.13 (m, 4H, H-6, H-8), 1.47 (t, *J* = 7.2 Hz, 3H, OCH_2_CH_3_), 1.17 (t, *J* = 7.2 Hz, 3H, CH_2_CH_3_), 1.05 (s, 3H, CH_3_), and 0.91 (s, 3H, CH_3_). ^13^C-NMR (150 MHz, CDCl_3_) was as follows: *δ* 195.5 (-CO), 167.3 (-CO), 165.6 (-CO), 148.4 (C), 147.9 (C), 145.2 (CH), 145.7 (C), 143.4 (C), 143.2 (C), 134.4 (C), 131.5 (CH), 130.8 (C), 129.7 (CH), 128.1 (CH), 127.1 (CH), 120.4 (CH), 116.7 (CH), 114.2 (CH), 112.1 (C), 105.8 (C), 70.5 (CH_2_), 64.6 (OCH_2_*CH_3_), 59.9 (CH_2_*CH_3_), 50.7 (CH_2_), 41.1 (CH_2_), 36.2 (CH), 32.7 (C), 29.4 (CH_3_), 27.2 (CH_3_), 19.5 (CH_3_), 15.1 (OCH_2_CH_3_*), and 14.3 (CH_2_CH_3_*). LC-HRMS (ESI^+^) was as follows: Found [M + H]^+^: 594.23510; cald. for C_32_H_37_ClN_3_O_6_: 594.23709.

Ethyl 4-(4-(2-(2-(3,4-dichlorobenzylidene)hydrazinyl)-2-oxoethoxy)-3-ethoxy phenyl)-2,7,7-trimethyl-5-oxo-1,4,5,6,7,8-hexahydroquinoline-3-carboxylate (**18**)

Yellow amorphous powder was as follows: Yield: 79%; M.P: 122–124 °C; ^1^H-NMR (600 MHz, CDCl_3_): *δ* 10.44 (s, 1H, H-10′), 8.53 (s, 1H, H-12′), 8.47 (s, 1H, H-1), 8.12 (d, *J* = 8.4 Hz, 1H, Ar-H, H-6″), 7.38 (s, 1H, Ar-H, H-2″), 7.26 (s, 1H, Ar-H, H-5″), 7.00 (s,1H, Ar-H, H-2′), 6.79–6.75 (m, 2H, H-5′, H-6′), 4.99 (s, 1H, H-4), 4.64 (s, 2H, H-8′), 4.16–4.02 (m, 4H, OCH_2_CH_3_, CH_2_CH_3_), 2.36 (s, 3H, CH_3_), 2.34–2.16 (m, 4H, H-6, H-8), 1.46 (t, *J* = 6.6 Hz, 3H, OCH_2_CH_3_), 1.17 (t, *J* = 6.6 Hz, 3H, CH_2_CH_3_), 1.05 (s, 3H, CH_3_), and 0.91 (s, 3H, CH_3_). ^13^C-NMR (150 MHz, CDCl_3_) was as follows: *δ* 195.5 (-CO), 167.3 (-CO), 165.7 (-CO), 148.4 (C), 145.7 (C), 144.0 (C-H), 143.3 (C-), 143.2 (C), 136.9 (C), 134.8 (2 x C), 130.0 (C), 129.6 (CH), 128.8 (CH), 127.7 (CH), 120.4 (CH), 116.8 (CH), 114.2 (CH), 112.1 (C), 105.8 (C), 70.6 (CH_2_), 64.6 (OCH_2_*CH_3_), 59.9 (CH_2_*CH_3_), 50.5 (CH_2_), 41.2 (CH_2_), 36.3 (CH), 32.7 (C), 29.4 (CH_3_), 27.2 (CH_3_), 19.5 (CH_3_), 15.1 (OCH_2_CH_3_*), and 14.3 (CH_2_CH_3_*). LC-HRMS (ESI^+^) was as follows: Found [M + H]^+^: 628.19870; cald. for C_32_H_36_Cl_2_N_3_O_6_: 628.19812.

Ethyl 4-(4-(2-(2-(3,4-dimethoxybenzylidene)hydrazinyl)-2-oxoethoxy)-3-ethoxy phenyl)-2,7,7-trimethyl-5-oxo-1,4,5,6,7,8-hexahydroquinoline-3-carboxylate (**19**)

Yellow amorphous powder was as follows: Yield: 72%; M.P: 122–124 °C; ^1^H-NMR (600 MHz, CDCl_3_): *δ* 10.13 (s, 1H, H-10′), 8.06 (s, 1H, H-12′), 7.43 (s, 1H, H-1), 7.09 (d, *J* = 7.8 Hz, 1H, Ar-H, H-6″), 6.99 (s, 1H, Ar-H, H-2″), 6.84 (d, *J* = 7.8 Hz, 1H, Ar-H, H-5″), 6.73 (s, 2H, Ar-H, H-5′, H-6′), 6.58 (br.s, 1H, Ar-H, H-2′), 4.98 (s, 1H, H-4), 4.60 (s, 2H, H-8′), 4.11–4.03 (m, 4H, OCH_2_CH_3_, CH_2_CH_3_), 3.89 (s, 3H, OCH_3_), 3.88 (s, 3H, OCH_3_), 2.36 (s, 3H, CH_3_), 2.31–2.14 (m, 4H, H-6, H-8), 1.43 (t, *J* = 6.6 Hz, 3H, OCH_2_CH_3_), 1.17 (t, *J* = 7.2 Hz, 3H, CH_2_CH_3_), 1.03 (s, 3H, CH_3_), and 0.90 (s, 3H, CH_3_). ^13^C-NMR (150 MHz, CDCl_3_) was as follows: *δ* 190.8 (-CO), 167.2 (-CO), 165.2 (-CO), 151.4 (C), 149.4 (CH), 148.1 (C), 145.4 (C), 143.3 (C), 142.8 (C), 130.0 (C), 126.8 (C), 126.1 (C), 123.1 (CH), 120.3 (CH), 116.4 (CH), 114.3 (CH), 111.6 (C), 110.5 (CH), 108.5 (CH), 106.0 (C), 70.1 (CH_2_), 64.5 (OCH_2_*CH_3_), 59.9 (CH_2_*CH_3_), 56.1 (OCH_3_), 56.0 (OCH_3_), 50.4 (CH_2_), 41.0 (CH_2_), 36.2 (CH), 32.5 (C), 29.3 (CH_3_), 27.2 (CH_3_), 19.4 (CH_3_), 15.0 (OCH_2_CH_3_*), and 14.3 (CH_2_CH_3_*). LC-HRMS (ESI^+^) was as follows: Found [M + H]^+^: 620.29385; cald. for C_34_H_42_N_3_O_8_: 620.29719.

Ethyl 4-(4′-(9′-(11′-(2″-hydroxy-3″-methoxybenzylidene)hydrazinyl)-9′-oxoe thoxy)-3′-ethoxyphenyl)-2,7,7-trimethyl-5-oxo-1,4,5,6,7,8-hexahydroquinoline-3-carboxylate (**20**)

Yellow amorphous powder was as follows: Yield: 78%; M.P: 137–139 °C; ^1^H-NMR (600 MHz, CDCl_3_): *δ* 10.39 (s, 1H, H-10′), 8.51 (s, 1H, H-12′), 8.47 (s, 1H, H-1), 6.97 (s, 1H, Ar-H, H-2′), 6.92 (d, *J* = 7.8 Hz, 1H, Ar-H, H-6″), 6.88 (d, *J* = 7.2 Hz, 1H, Ar-H, H-3″), 6.84 (t, *J* = 7.8 Hz, 1H, Ar-H, H-5″), 6.74 (br.s, 2H, Ar-H, H-5′, H-6′), 4.98 (s, 1H, H-4), 4.59 (s, 2H, H-8′), 4.09–4.03 (m, 4H, OCH_2_CH_3_, CH_2_CH_3_), 3.88 (s, 3H, OCH_3_), 2.35 (s, 3H, CH_3_), 2.32–2.16 (m, 4H, H-6, H-8), 1.42 (t, *J* = 6.6 Hz, 3H, OCH_2_CH_3_), 1.17 (t, *J* = 7.2 Hz, 3H, CH_2_CH_3_), 1.04 (s, 3H, CH_3_), and 0.91 (s, 3H, CH_3_). ^13^C-NMR (150 MHz, CDCl_3_) was as follows: *δ* 195.3 (-CO), 167.2 (-CO), 165.3 (-CO), 151.8 (C-H), 148.5 (C), 148.3 (2 × C), 145.7 (C), 143.3 (C), 124.5 (C), 122.6 (CH), 120.4 (CH), 119.5 (C), 119.1 (CH), 117.6 (CH), 114.2 (C), 114.1 (CH), 111.5 (C), 106.1 (C), 70.1 (CH_2_), 64.6 (OCH_2_*CH_3_), 60.0 (CH_2_*CH_3_), 56.2 (OCH_3_), 50.1 (CH_2_), 41.0 (CH_2_), 36.3 (CH), 32.7 (C), 29.2 (CH_3_), 27.2 (CH_3_), 19.3 (CH_3_), 15.0 (OCH_2_CH_3_*), and 14.3 (CH_2_CH_3_*). LC-HRMS (ESI^+^) was as follows: Found [M + H]^+^: 606.27595; cald. for C_33_H_40_N_3_O_8_: 606.28154.

Ethyl 4-(3-ethoxy-4-(2-(2-((5-methylfuran-2-yl)methylene)hydrazinyl)-2-oxoethoxy)phenyl)-2,7,7-trimethyl-5-oxo-1,4,5,6,7,8-hexahydroquinoline-3-carboxylate (**21**)

Pale yellow amorphous powder was as follows: Yield: 76%; M.P: 112–114 °C; ^1^H-NMR (600 MHz, CDCl_3_): *δ* 10.04 (s, 1H, H-10′), 8.50 (s, 1H, H-12′), 8.06 (s, 1H, H-1), 6.99–6.97 (m, 2H, Ar-H, H-2″, H-3″), 6.07 (br.s, 2H, Ar-H, H-5′, H-6′), 6.62 (s, 1H, Ar-H, H-2′), 4.97 (s, 2H, H-8′), 4.64 (s, 1H, H-4), 4.05–4.02 (m, 4H, OCH_2_CH_3_, CH_2_CH_3_), 2.31 (s, 6H, 2CH_3_), 2.30–2.05 (m, 4H, H-6, H-8), 1.43 (t, *J* = 7.2 Hz, 3H, OCH_2_CH_3_), 1.17 (t, *J* = 7.2 Hz, 3H, CH_2_CH_3_), 1.03 (s, 3H, CH_3_), and 0.91 (s, 3H, CH_3_). ^13^C-NMR (150 MHz, CDCl_3_) was as follows: *δ* 195.7 (-CO), 167.5 (-CO), 165.5 (-CO), 155.6 (C), 152.3 (C), 149.9 (C), 148.3 (C), 147.3 (C), 145.6 (C), 143.2 (CH), 132.5 (C), 120.2 (CH), 116.4 (CH), 114.1 (CH), 111.9 (C), 110.1 (CH), 106.7 (CH), 105.6 (C), 70.2 (CH_2_), 64.4 (CH_2_*CH_3_), 59.8 (OCH_2_*CH_3_), 50.7 (CH_2_), 40.9 (CH_2_), 36.2 (CH), 32.7 (C), 29.4 (CH_3_), 27.2 (CH_3_), 19.3 (CH_3_), 15.1 (OCH_2_CH_3_*), 14.3 (CH_2_CH_3_*), and 13.4 (CH_3_). LC-HRMS (ESI^+^) was as follows: Found [M + H]^+^: 564.27044; cald. for C_31_H_38_N_3_O_7_: 564.27098.

Ethyl 4-(3-ethoxy-4-(2-(2-(3-methoxybenzylidene)hydrazinyl)-2-oxoethoxy)phen yl)-2,7,7-trimethyl-5-oxo-1,4,5,6,7,8-hexahydroquinoline-3-carboxylate (**22**)

Yellow amorphous powder was as follows: Yield: 69%; M.P: 112–114 °C; ^1^H-NMR (600 MHz, CDCl_3_): *δ* 10.24 (s, 1H, H-10′), 8.51 (s, 1H, H-12′), 8.13 (s, 1H, H-1), 7.31 (s, 1H, Ar-H, H-2″), 7.28 (t, *J* = 7.8 Hz, 1H, Ar-H, H-5″), 7.22 (d, *J* = 7.8 Hz, 1H, Ar-H, H-6″), 6.98 (s, 1H, Ar-H, H-2′), 6.94 (d, *J* = 6.6 Hz, 1H, Ar-H, H-4″), 6.73 (br.s, 2H, Ar-H, H-5′, H-6′), 4.98 (s, 1H, H-4), 4.61 (s, 2H, H-8′), 4.11–4.02 (m, 4H, OCH_2_CH_3_, CH_2_CH_3_), 3.81 (s, 3H, OCH_3_), 2.37 (s, 3H, CH_3_), 2.34–2.20 (m, 4H, H-6, H-8), 1.43 (t, *J* = 7.2 Hz, 3H, OCH_2_CH_3_), 1.17 (t, *J* = 7.2 Hz, 3H, CH_2_CH_3_), 1.03 (s, 3H, CH_3_), and 0.91 (s, 3H, CH_3_). ^13^C-NMR (150 MHz, CDCl_3_) was as follows: *δ* 192.2 (-CO), 167.2 (-CO), 165.6 (-CO), 159.9 (C), 149.3 (CH), 148.4 (C), 145.6 (C), 143.3 (C), 142.9 (C), 134.7 (C), 130.0 (C), 129.7 (CH), 121.1 (CH), 120.3 (CH), 117.5 (CH), 116.1 (CH), 114.2 (CH), 112.0 (C), 111.4 (CH), 106.4 (C), 70.3 (CH_2_), 64.6 (OCH_2_*CH_3_), 59.9 (CH_2_*CH_3_), 55.5 (OCH_3_), 50.1 (CH_2_), 41.0 (CH_2_), 36.3 (CH), 32.7 (C), 29.3 (CH_3_), 27.2 (CH_3_), 19.3 (CH_3_), 15.0 (OCH_2_CH_3_*), and 14.3 (CH_2_CH_3_*). LC-HRMS (ESI^+^) was as follows: Found [M + H]^+^: 590.26255; cald. for C_33_H_40_N_3_O_7_: 590.28663.

Ethyl 4-(3-ethoxy-4-(2-(2-(4-methoxybenzylidene)hydrazinyl)-2-oxoethoxy)phen yl)-2,7,7-trimethyl-5-oxo-1,4,5,6,7,8-hexahydroquinoline-3-carboxylate (**23**)

Yellow amorphous powder was as follows: Yield: 70%; M.P: 119–121 °C; ^1^H-NMR (600 MHz, CDCl_3_): *δ* 10.11 (s, 1H, H-10′), 8.06 (s, 1H, H-12′), 7.67 (d, *J* = 7.2 Hz, 2H, Ar-H, H-2″, H-6″), 7.02 (s, 1H, H-1), 6.88 (d, *J* = 7.8 Hz, 2H, Ar-H, H-3″, H-5″), 6.74 (s, 2H, Ar-H, H-5′, H-6′), 6.63 (br.s, 1H, Ar-H, H-2′), 4.99 (s, 1H, H-4), 4.60 (s, 2H, H-8′), 4.11–4.03 (m, 4H, OCH_2_CH_3_, CH_2_CH_3_), 3.81 (s, 3H, OCH_3_), 2.36 (s, 3H, CH_3_), 2.32–2.14 (m, 4H, H-6, H-8), 1.43 (t, *J* = 6.6 Hz, 3H, OCH_2_CH_3_), 1.17 (t, *J* = 6.6 Hz, 3H, CH_2_CH_3_), 1.03 (s, 3H, CH_3_), and 0.90 (s, 3H, CH_3_). ^13^C-NMR (150 MHz, CDCl_3_) was as follows: *δ* 195.5 (-CO), 167.4 (-CO), 165.4 (-CO), 161.8 (C), 149.1 (CH), 148.3 (C), 145.6 (C), 143.6 (C), 143.0 (C), 132.0 (C), 129.5 (2 × C), 125.9 (C), 120.3 (CH), 116.1 (CH), 114.3 (CH), 114.2 (2 × C), 111.7 (C), 106.0 (C), 70.1 (CH_2_), 64.6 (OCH_2_*CH_3_), 59.9 (CH_2_*CH_3_), 55.4 (OCH_3_), 50.4 (CH_2_), 41.0 (CH_2_), 36.2 (CH), 32.7 (C), 29.3 (CH_3_), 27.2 (CH_3_), 19.4 (CH_3_), 15.1 (OCH_2_*CH_3_), and 14.3 (CH_2_*CH_3_). LC-HRMS (ESI^+^) was as follows: Found [M + H]^+^: 590.27147; cald. for C_33_H_40_N_3_O_7_: 590.28663.

Ethyl 4-(3-ethoxy-4-(2-(2-(4-fluorobenzylidene)hydrazinyl)-2-oxoethoxy)phen yl)-2,7,7-trimethyl-5-oxo-1,4,5,6,7,8-hexahydroquinoline-3-carboxylate (**24**)

Yellow amorphous powder was as follows: Yield: 87%; M.P: 166–168 °C; ^1^H-NMR (600 MHz, CDCl_3_): *δ* 10.22 (br.s, 1H, H-10′), 8.15 (br.s, 1H, H-12′), 7.72–7.70 (m, 2H, Ar-H, H-2″, H-6″), 7.06–7.04 (m, 2H, Ar-H, H-3″, H-5″), 6.99 (s, 1H, H-1), 6.76–6.72 (m, 2H, Ar-H, H-5′, H-6′), 6.56 (br.s, 1H, Ar-H, H-2′), 4.98 (s, 1H, H-4), 4.61 (s, 2H, H-8′), 4.13–4.02 (m, 4H, OCH_2_CH_3_, CH_2_CH_3_), 2.36 (s, 3H, CH_3_), 2.32–2.15 (m, 4H, H-6, H-8), 1.43 (t, *J* = 7.2 Hz, 3H, OCH_2_CH_3_), 1.17 (t, *J* = 7.2 Hz, 3H, CH_2_CH_3_), 1.04 (s, 3H, CH_3_), and 0.91 (s, 3H, CH_3_). ^13^C-NMR (150 MHz, CDCl_3_) was as follows: *δ* 190.5 (-CO), 167.3 (-CO), 165.6 (-CO), 163.4 (C), 148.3 (C), 148.0 (CH), 145.6 (C), 143.3 (C), 143.0 (C), 132.3 (C), 129.8 (CH), 129.6 (C), 129.7 (CH), 120.3 (CH), 116.5 (CH), 116.3 (CH), 115.8 (CH), 114.3 (CH), 111.7 (C), 106.0 (C), 70.1 (CH_2_), 64.6 (OCH_2_*CH_3_), 59.9 (CH_2_*CH_3_), 50.2 (CH_2_), 41.0 (CH_2_), 36.3 (CH), 32.7 (C), 29.3 (CH_3_), 27.2 (CH_3_), 19.3 (CH_3_), 15.0 (OCH_2_CH_3_*), and 14.3 (CH_2_CH_3_*). LC-HRMS (ESI^+^) was as follows: Found [M + H]^+^: 578.26084; cald. for C_32_H_37_FN_3_O_6_: 578.26664.

Ethyl 4-(3-ethoxy-4-(2-(2-(2-hydroxybenzylidene)hydrazinyl)-2-oxoethoxy)phen yl)-2,7,7-trimethyl-5-oxo-1,4,5,6,7,8-hexahydroquinoline-3-carboxylate (**25**)

Brown amorphous powder was as follows: Yield: 73%; M.P: 121–123 °C; ^1^H-NMR (600 MHz, CDCl_3_): *δ* 10.24 (br.s, 1H, H-10′), 8.42 (br.s, 1H, H-12′), 7.29 (s, 1H, H-1), 7.23 (s, 1H, Ar-H, H-3″), 7.02–6.98 (m, 3H, Ar-H, H-4″, H-5″, H-6″), 6.89 (s, 1H, Ar-H, H-2′), 6.79 (br.s, 2H, Ar-H, H-5′, H-6′), 5.00 (s, 1H, H-4), 4.62 (br.s, 2H, H-8′), 4.06 (br.s, 4H, OCH_2_CH_3_, CH_2_CH_3_), 2.39 (br.s, 3H, CH_3_), 2.33–2.20 (m, 4H, H-6, H-8), 1.46 (br.s, 3H, OCH_2_CH_3_), 1.18 (br.s, 3H, CH_2_CH_3_), 1.07 (s, 3H, CH_3_), and 0.94 (s, 3H, CH_3_). ^13^C-NMR (150 MHz, CDCl_3_) was as follows: *δ* 195.4 (-CO), 167.3 (-CO), 165.1 (-CO), 158.7 (C), 151.9 (CH), 148.4 (C), 145.7 (C), 143.3 (C), 137.0 (C), 133.7 (C), 132.1 (CH), 131.1 (CH), 120.7 (C), 119.4 (CH), 117.3 (2×CH), 117.2 (CH), 114.4 (CH), 112.1 (C), 106.1 (C), 70.3 (CH_2_), 65.0 (OCH_2_*CH_3_), 59.9 (CH_2_*CH_3_), 50.7 (CH_2_), 41.2 (CH_2_), 36.3 (CH), 32.7 (C), 29.5 (CH_3_), 29.7 (CH_3_), 19.3 (CH_3_), 15.2 (OCH_2_CH_3_), 14.3 (CH_2_CH_3_*). LC-HRMS (ESI^+^) was as follows: Found [M + H]^+^: 576.28016; cald. for C_32_H_38_N_3_O_7_: 576.27098.

Ethyl 4-(3-ethoxy-4-(2-(2-(naphthalen-1-ylmethylene)hydrazinyl)-2-oxoethoxy)phenyl)-2,7,7-trimethyl-5-oxo-1,4,5,6,7,8-hexahydroquinoline-3-carboxylate (**26**)

Dark yellow amorphous powder was as follows: Yield: 80%; M.P: 98–100 °C; ^1^H-NMR (600 MHz, CDCl_3_): *δ* 10.38 (s, 1H, H-10′), 9.24 (d, *J* = 8.4 Hz, 1H, Ar-H, H-2″), 8.85 (s, 1H, H-1), 8.59 (d, *J* = 8.4 Hz, 1H, Ar-H, H-8″), 8.09 (d, *J* = 8.4 Hz, 1H, Ar-H, H-7″), 7.98 (t, *J* = 8.4 Hz, 2H, Ar-H, H-4″, H-5″), 7.62–7.43 (m, 3H, Ar-H, H-2′, H-3″, H-6″), 7.01 (s, 1H, H-12′), 6.79–6.76 (m, 2H, Ar-H, H-5′, H-6′), 5.00 (s, 1H, H-4), 4.65 (s, 2H, H-8′), 4.12–4.03 (m, 4H, OCH_2_CH_3_, CH_2_CH_3_), 2.37 (s, 3H, CH_3_), 2.33–2.14 (m, 4H, H-6, H-8), 1.46 (t, *J* = 7.2 Hz, 3H, OCH_2_CH_3_), 1.17 (t, *J* = 7.2 Hz, 3H, CH_2_CH_3_), 1.00 (s, 3H, CH_3_), and 0.89 (s, 3H, CH_3_). ^13^C-NMR (150 MHz, CDCl_3_) was as follows: *δ* 193.6 (-CO), 167.4 (-CO), 165.6 (-CO), 148.4 (CH), 145.7 (C), 143.6 (C), 143.1 (C), 133.8 (C), 133.7 (C), 131.4 (C), 130.5 (C), 129.1 (CH), 128.9 (CH), 128.5 (CH), 128.0 (CH), 127.4 (C-), 127.0 (CH), 126.2 (CH), 120.3 (CH), 116.7 (CH), 114.2 (CH), 111.6 (C), 105.7 (C), 70.4 (CH_2_), 64.4 (OCH_2_*CH_3_), 59.9 (CH_2_*CH_3_), 50.3 (CH_2_), 40.9 (CH_2_), 36.3 (CH), 32.7 (C), 29.3 (CH_3_), 27.1 (CH_3_), 19.3 (CH_3_), 15.1 (OCH_2_*CH_3_), and 14.3 (CH_2_*CH_3_). LC-HRMS (ESI^+^) was as follows: Found [M + H]^+^: 610.27554; cald. for C_36_H_40_N_3_O_6_: 610.29171.

Ethyl 4-(4-(2-(2-butylidenehydrazinyl)-2-oxoethoxy)-3-ethoxyphenyl)-2,7,7-trimethyl-5-oxo-1,4,5,6,7,8-hexahydroquinoline-3-carboxylate (**27**)

Brown oil was as follows: Yield: 82%; ^1^H-NMR (600 MHz, CDCl_3_): *δ* 10.04 (br.s, 1H, H-10′), 7.44 (br.s, 1H, H-1), 7.30 (br.s, 1H, H-12′), 6.93 (s, 1H, Ar-H, H-2′), 6.71 (s, 2H, Ar-H, H-5′, H-6′), 4.93 (s, 1H, H-4), 4.50 (s, 2H, H-8′), 4.03–3.98 (m, 4H, OCH_2_CH_3_, CH_2_CH_3_), 2.29 (s, 3H, CH_3_), 2.23–1.45 (m, 8H, H-6, H-8, H-13′, H-14′), 1.39 (t, *J* = 7.2 Hz, 3H, OCH_2_CH_3_), 1.15 (t, *J* = 7.2 Hz, 3H, CH_2_CH_3_), 0.98 (s, 3H, CH_3_), 0.89–0.85 (m, 3H, H-15′), and 0.83 (s, 3H, CH_3_). ^13^C-NMR (150 MHz, CDCl_3_) was as follows: *δ* 195.9 (-CO), 167.6 (-C), 165.4 (-CO), 153.3 (C-12′), 149.5 (C-2), 148.3 (C), 145.5 (C), 143.4 (C), 134.2 (C), 120.2 (CH), 116.3 (CH), 114.1 (CH), 111.4 (C), 105.4 (C), 70.1 (C-), 64.4 (OCH_2_*CH_3_), 59.8 (CH*_2_CH_3_), 50.7 (CH), 40.6 (CH_2_), 36.2 (CH), 32.5 (C), 29.5 (CH_3_), 27.0 (CH_3_), 23.0 (C-13′), 19.9 (CH_3_), 19.4 (C-14′), 15.0 (OCH_2_CH_3_*), 14.3 (CH_2_CH_3_*), and 13.9 (C-15′). LC-HRMS (ESI^+^) was as follows: Found [M + H]^+^: 526.2812; C_29_H_39_N_3_O_6_.

### 4.8. Antibacterial Bioassay

The agar-well diffusion method was used to check the antibacterial activities of the compounds. Two bacterial strains clinical isolates of Khyber Medical University were used in the study, i.e., *Escherichia coli* and *Enterococcus faecalis.* First, Mueller-Hinton Broth (MHB) was prepared by dissolving 0.2 g of MHB in 100 mL of distilled water; the pH was adjusted to 7.0 and was autoclaved. Then, Mueller-Hinton agar (MHA) was prepared by dissolving 3.8 g of MHA medium in 100 mL of distilled water; pH was adjusted to 7.0 and was autoclaved at 121 °C. After autoclaving, the medium was poured into petri plates. A 0.5 McFarland standard was prepared by mixing 0.05 mL of 1.175% barium chloride dihydrate (BaCl_2_·2H_2_O) with 9.95 mL of 1% sulfuric acid (H_2_SO_4_). A 0.5 McFarland standard corresponds to approximately 1.5 × 10^8^ cells/mL. The bacteria were cultured one day before the assay in MHB suspension. The bacterial inoculum was adjusted according to 0.5 McFarland standards and added to nutrient agar plates. Then, 4 mm wells were made in the MHA plates. The test compounds were added to the individual wells of the petri plates and incubated at 37 °C for 24 h. The zones of inhibition of the test compounds were measured in millimeter (mm) after 24 h.

### 4.9. In Vitro Calcium-Channel-Blocking Study in Isolated Aorta from SD Rats

As described previously [49], thoracic aorta was isolated from normotensive and hypertensive SD rats and transferred to a petri dish containing normal Kreb’s solution. Rings were made 2–3 mm in width. Each ring was placed in a tissue bath filled with normal Kreb’s solution. The composition of the Kreb’s solution was (mM), as follows: NaCl 118.2, NaHCO_3_ 25.0, CaCl_2_ 2.5, KCl 4.7, KH_2_PO_4_ 1.3, MgSO_4_ 1.2, and glucose 11.7 (pH 7.4), bubbled with carbogen (95% O_2_ and 5% CO_2_), and attached to a force transducer (MLT 0201) that was coupled with a bridge amplifier (N12128) and a Power Lab (ML 846) Data Acquisition System. The rings were allowed to equilibrate for 60–90 min at a resting tension of 2 g and the bath solution was changed every 15 min. All tissues were stabilized and the administration of phenylephrine (1 μM) was repeated. The sustained contractions were induced with high K^+^ and cumulative addition of different concentrations (0.03–100 µg/mL) of test compounds (**1**–**27**) were made to determine the effect on vascular tone. The interval between each concentration was 10–15 min. The effect of the test compounds on vascular tension was calculated as a percentage of the high K^+^ control.

## Figures and Tables

**Table 1:** Different aromatic/aliphatic (R) substituents of PHQ derivatives and their yields (%).

Compounds	R	Yield	Compounds	R	Yield
**3**	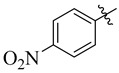	81	**16**	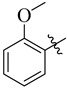	65
**4**	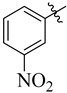	75	**17**	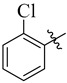	73
**5**	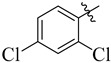	69	**18**	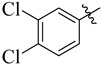	79
**6**	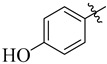	71	**19**	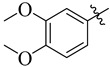	72
**7**	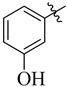	72	**20**	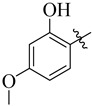	78
**8**	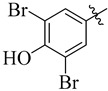	81	**21**	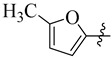	76
**9**	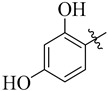	74	**22**	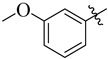	69
**10**	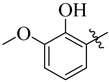	68	**23**	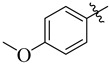	70
**11**	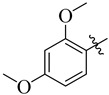	82	**24**	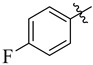	87
**12**	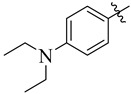	70	**25**	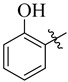	73
**13**	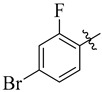	67	**26**	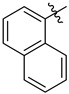	80
**14**	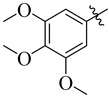	66	**27**	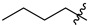	82
**15**	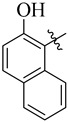	81			

**Table 2:** Antibacterial activities of the synthesized compounds against different bacterial clinical isolates at three different concentrations.

Code	*Escherichia coli* (mm)			*Enterococcus faecalis* (mm)		
	2 µg/mL	1 µg/mL	0.5 µg/mL	2 µg/mL	1 µg/mL	0.5 µg/mL
**17**	18	16	14	18	16	12
**21**	18	16	14	18	16	14
**22**	18	16	14	18	15	13
**1**	18	12	9	18	14	12
**16**	16	14	10	18	12	10
**8**	16	14	12	18	16	14
**2**	16	10	7	16	14	10
**26**	14	12	8	16	14	10
**13**	14	10	6	16	10	8
**3**	12	10	8	14	10	8
**12**	12	10	8	14	12	10
**9**	10	8	4	10	8	4
**18**	10	8	6	10	8	6
**5**	10	6	4	10	6	4
**27**	8	6	4	10	8	4
**Amoxicillin**	30	24	22	32	28	24

*Escherichia coli* (gram-negative bacterium); *Enterococcus faecalis* (gram-positive bacterium). Above 18 mm (significant activity), 16–18 mm (good activity), 13–15 mm (low activity), 9–12 mm (non-significant), below 9 mm (no activity).

## Data Availability

The spectroscopic data presented in this study are available in the supporting information.

## Supplementary Materials

The following supporting information can be downloaded at: https://www.mdpi.com/article/10.3390/antibiotics11111568/s1, Figure S1: The effect of synthesized compounds against high K+ (80 mM) induced contraction (*n* = 3–5), values represented as mean ± SEM using two-way ANOVA; Figure S2: 1H-NMR and 13C-NMR spectra of ethyl-2-(2-ethoxy-4-formylphenoxy)acetate; Figure S3: ^1^H-NMR and ^13^C-NMR spectra of compound **1**; Figure S4: ^1^H-NMR and ^13^C-NMR spectra of compound **2**; Figure S5: Mass, ^1^H-NMR, and ^13^C-NMR spectra of compound **3**; Figure S6: Mass, ^1^H-NMR, and ^13^C-NMR spectra of compound **4**; Figure S7: Mass, ^1^H- and ^13^C-NMR spectra of compound **5**; Figure S8: Mass, ^1^H- and ^13^C-NMR spectra of compound **6**; Figure S9: Mass, ^1^H- and ^13^C-NMR spectra of compound **7**; Figure S10: Mass, ^1^H- and ^13^C-NMR spectra of compound **8**; Figure S11: Mass, ^1^H- and ^13^C-NMR spectra of compound **9**; Figure S12: Mass, ^1^H- and ^13^C-NMR spectra of compound **10**; Figure S13: Mass, ^1^H- and ^13^C-NMR spectra of compound **11**; Figure S14: Mass, ^1^H- and ^13^C-NMR spectra of compound **12**; Figure S15: Mass, ^1^H- and ^13^C-NMR spectra of compound **13**; Figure S16: Mass, ^1^H- and ^13^C-NMR spectra of compound **14**; Figure S17: Mass, ^1^H- and ^13^C-NMR spectra of compound **15**; Figure S18: Mass, ^1^H- and ^13^C-NMR spectra of compound **16**; Figure S19: Mass, ^1^H- and ^13^C-NMR spectra of compound **17**; Figure S20: Mass, ^1^H- and ^13^C-NMR spectra of compound **18**; Figure S21: Mass, ^1^H- and ^13^C-NMR spectra of compound **19**; Figure S22: Mass, ^1^H-NMR and ^13^C-NMR spectra of compound **20**; Figure S23: Mass and ^1^H-NMR spectra of compound **21**; Figure S24: Mass, ^1^H- and ^13^C-NMR spectra of compound **22**; Figure S25: Mass, ^1^H- and ^13^C-NMR spectra of compound **23**; Figure S26: Mass, ^1^H- and ^13^C-NMR spectra of compound **24**; Figure S27: Mass, ^1^H- and ^13^C-NMR spectra of compound **25**; Figure S28: Mass, ^1^H- and ^13^C-NMR spectra of compound **26**; Figure S29. HR-ESI-MS, ^1^H- and ^13^C-NMR spectra of compounds **27**.
