# Supplementary material for: Novel Polyhydroquinoline-Hydrazide-Linked Schiff’s Base Derivatives: Multistep Synthesis, Antimicrobial, and Calcium-Channel-Blocking Activities
Source: Antibiotics (Basel). 2022 Nov 7;11(11):1568. doi: 10.3390/antibiotics11111568 (PMC9686546; doi:10.3390/antibiotics11111568)
Supplement: Supplementary file 1 [file antibiotics-11-01568-s001.zip › antibiotics-1964888-supplementary.pdf]

Novel Polyhydroquinoline Hydrazide Linked Schiff's Base Derivatives: Multistep Synthesis, Antimicrobial and Calcium Channel Blocking Activities

Zainab[1,2], Yu Haitao[1*], Najeeb Ur Rehman[3*], Mumtaz Ali[2], Aftab Alam[2], Abdul Latif[2], Nazish Shahab[4], Irfan Amir Khan[5], Abdul Jabbar Shah[5], Momin Khan[6], Ahmed Al-Ghafri[3], Ahmed Al-Harrasi[3*], Manzoor Ahmad[2*]

[1]College of Chemistry and Materials Science, Hebei Normal University, Shijiazhuang 050024, China; abidkhan4199@gmail.com (Z.)

[2]Department of Chemistry, University of Malakand, Chakdara, Dir (L), 18800, Khyber Pakhtunkhwa, Pakistan; aftab.alam@uom.edu.pk (A.A.); mumtazali@uom.edu.pk (M.A.); drlatif2016@gmail.com (A.L.).

[3]Natural & Medical Sciences Research Center, University of Nizwa, PC 616, Birkat Al Mauz, Nizwa P.O. Box 33, Oman

[4]State Key Laboratory of Chemical Resource Engineering, Beijing Engineering Center for Hierarchical Catalysis, Beijing Advanced Innovation Center for Soft Matter Science and Engineering, Beijing university of Chemical Technology, Beijing, 100029, China

[5]Department of Pharmacy, COMSATS University Islamabad Abbottabad Campus-22060, Khyber Pakhtunkhwa, Pakistan; jabbarshah@cuiatd.eu.pk (A.J.S); afswat117@yahoo.com (I.A.K).

[6]Institute of Pathology and Diagnostic Medicine, Khyber Medical University, Hayatabad Peshawar, Khyber Pakhtunkhwa, Pakistan; mominkhan.ibms@kmu.pk (M.K).

***Corresponding emails:** haitaoyu@hebtu.edu.cn (Y.H); najeeb@unizwa.edu.om (N.U.R); aharrasi@unizwa.edu.om (A H); manzoorahmad@uom.edu.pk (M.A).

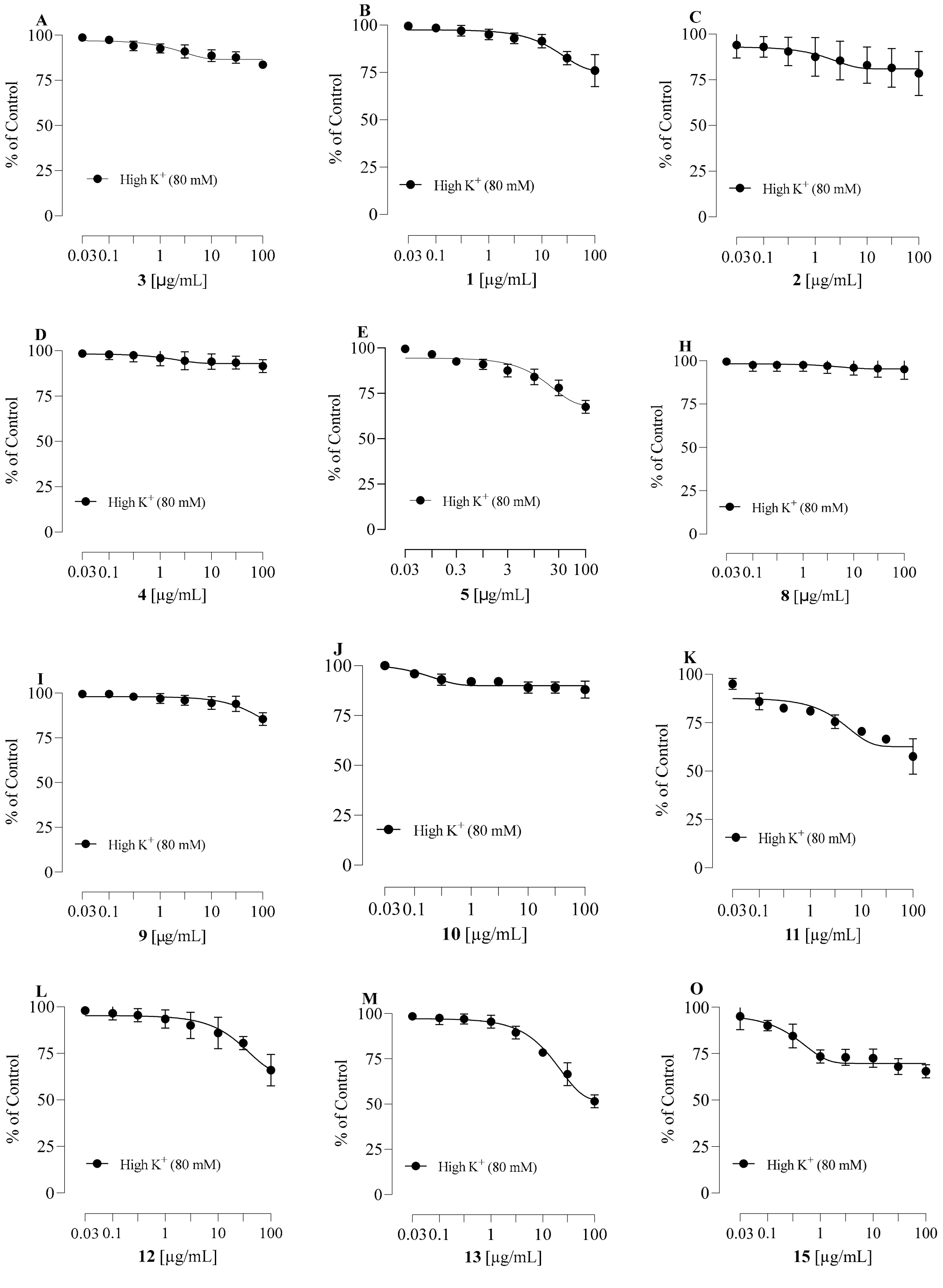
A
B
C
D
E
H
I
J
K
L
M
O
% of Control
High K+ (80 mM)
3 [µg/mL]
1 [µg/mL]
2 [µg/mL]
4 [µg/mL]
5 [µg/mL]
8 [µg/mL]
9 [µg/mL]
10 [µg/mL]
11 [µg/mL]
12 [µg/mL]
13 [µg/mL]
15 [µg/mL]

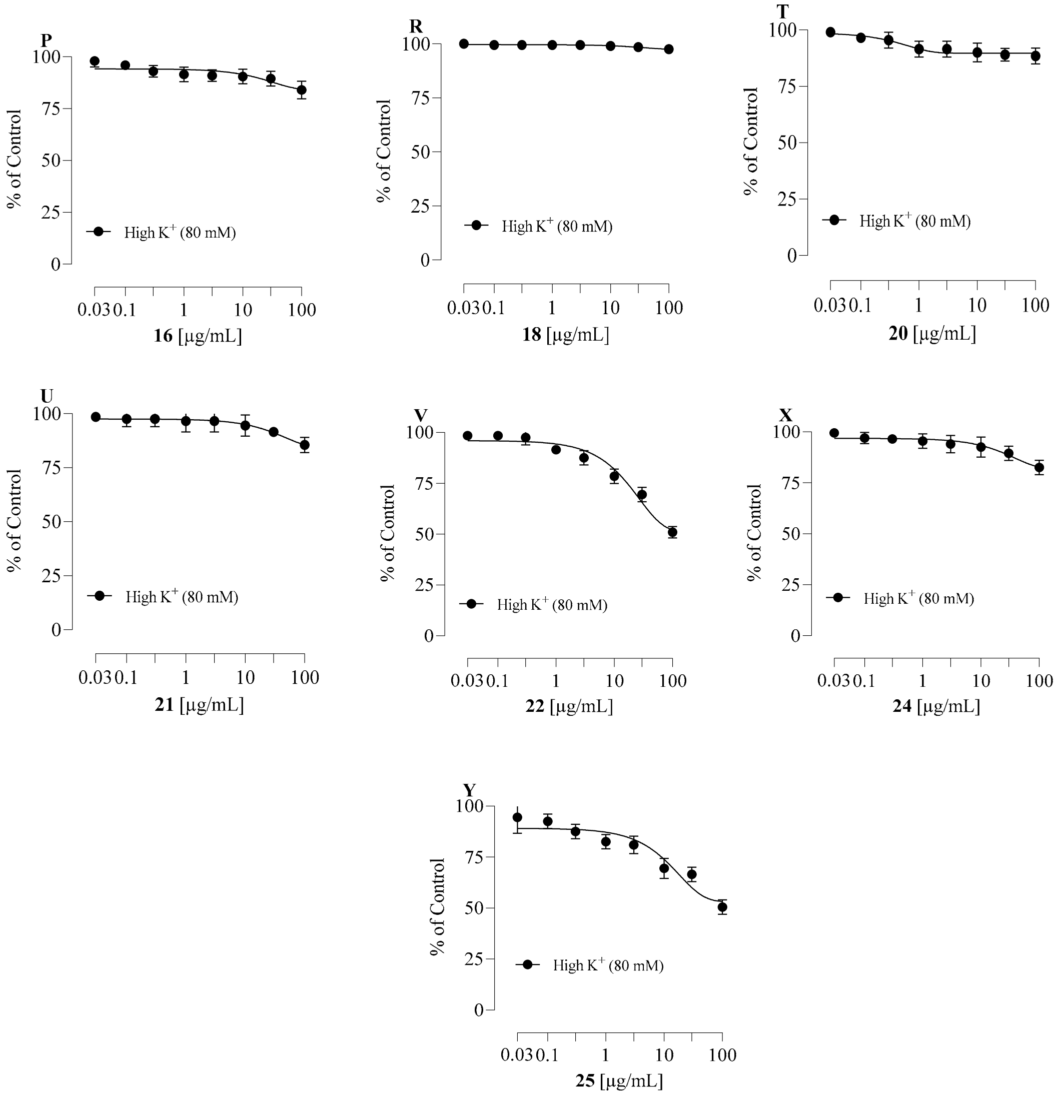

Figure S1: The effect of synthesized compounds against high K^+ (80 mM) induced contraction (n = 3–5), values represented as mean ± SEM using two-way ANOVA.

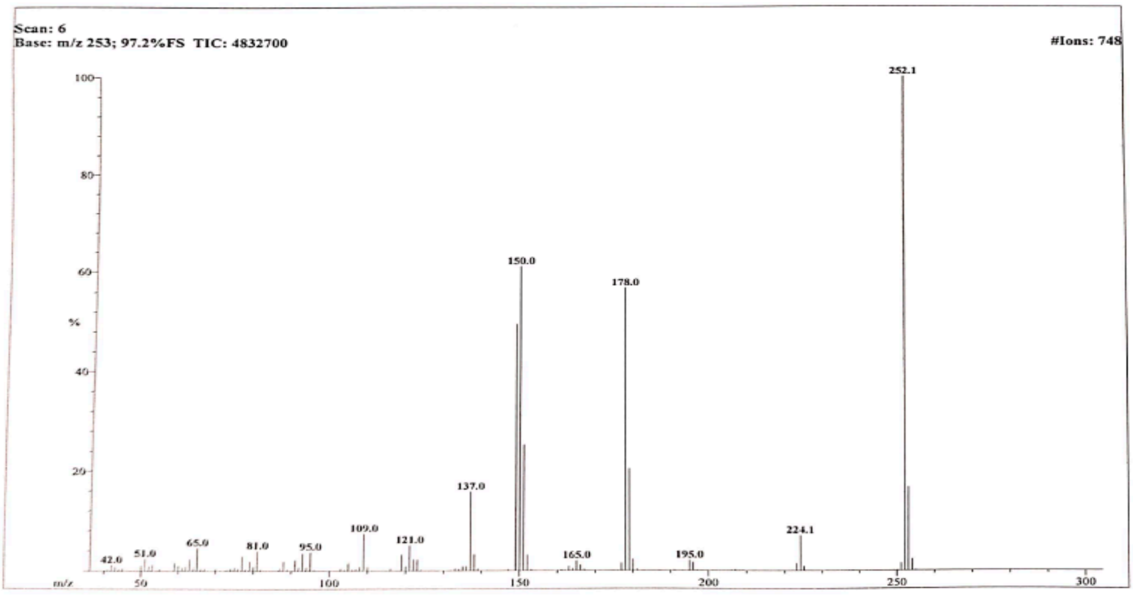

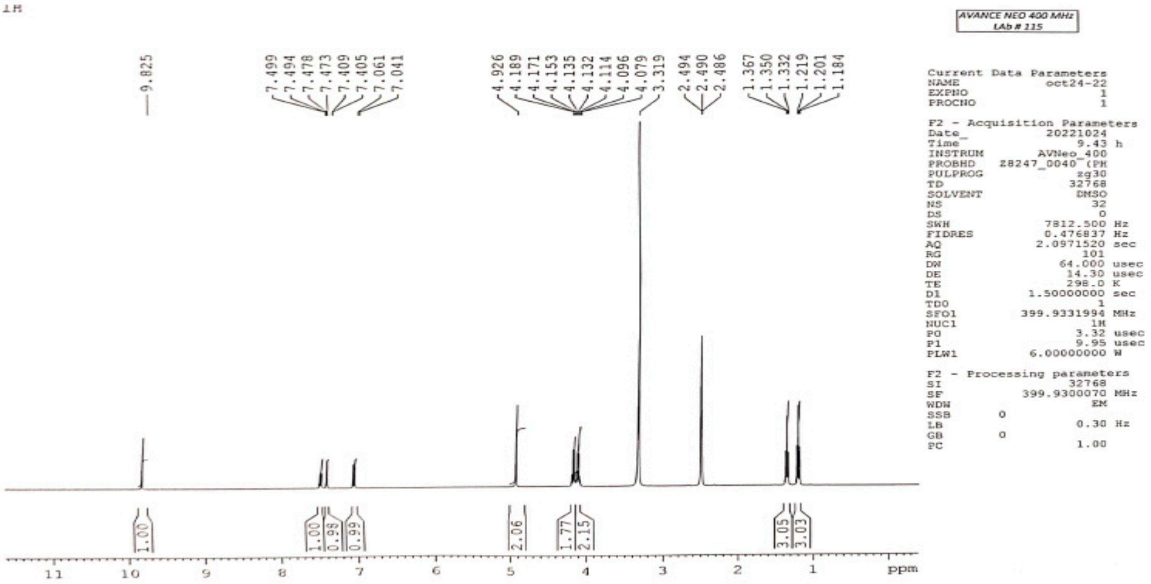

Figure S2: ^{1}H-NMR and ^{13}C-NMR spectra of ethyl-2-(2-ethoxy-4-formylphenoxy)acetate

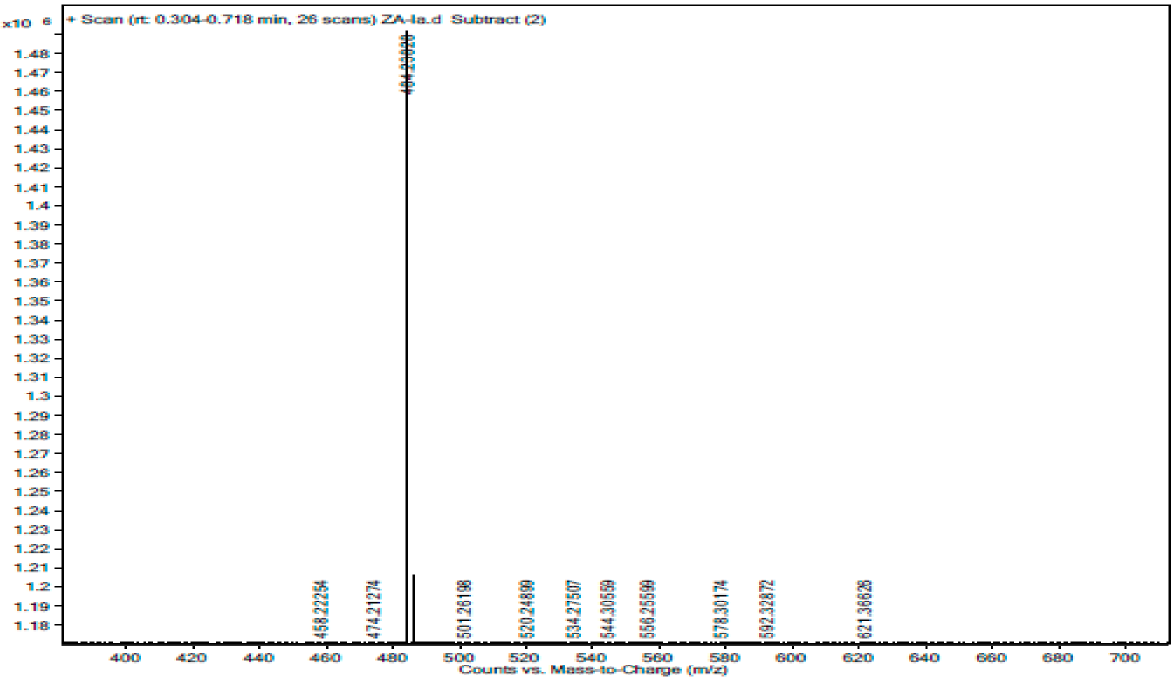
+ Scan (rt: 0.304-0.718 min, 26 scans) ZA-Ia.d Subtract (2)
x10 6
458.22254
474.21274
501.26198
520.24890
534.27507
544.30559
558.25599
578.30174
592.32872
621.36828
Counts vs. Mass-to-Charge (m/z)

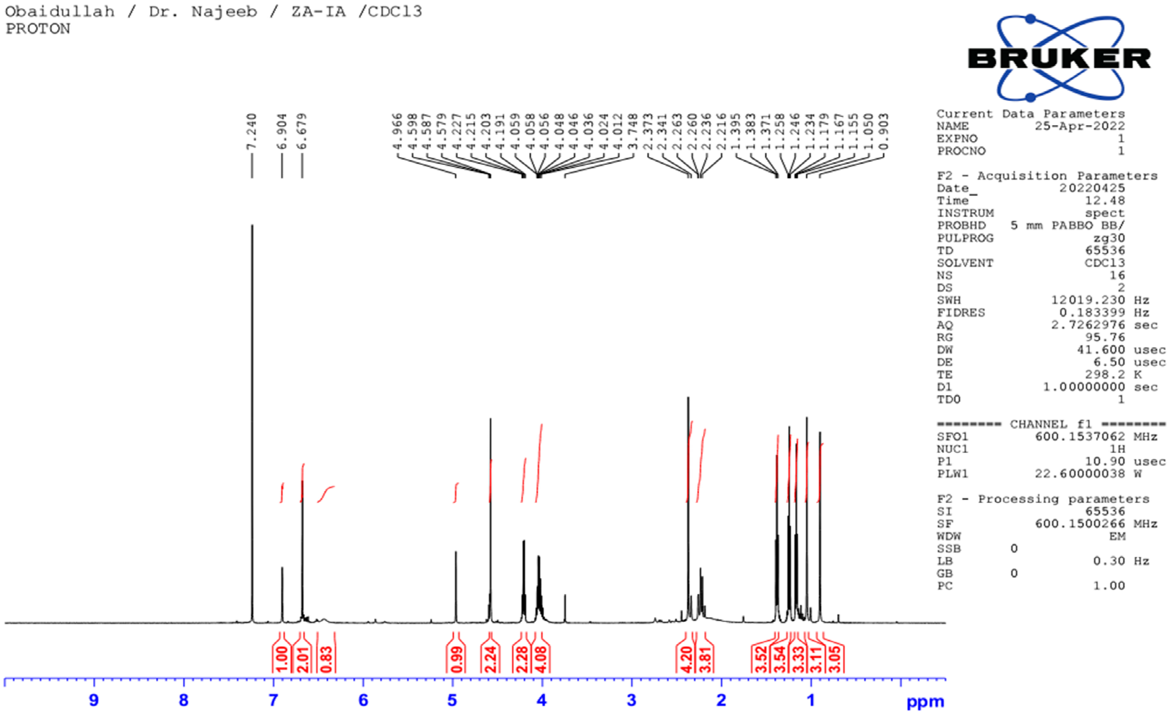
Obaidullah / Dr. Najeeb / ZA-IA /CDCl3
PROTON
Current Data Parameters
NAME 25-Apr-2022
EXPNO 1
PROCNO 1
F2 - Acquisition Parameters
Date_ 20220425
Time 12.48
INSTRUM spect
PROBHD 5 mm PABBO BB/
PULPROG zg30
TD 65536
SOLVENT CDCl3
NS 16
DS 2
SWH 12019.230 Hz
FIDRES 0.183399 Hz
AQ 2.7262976 sec
RG 95.76
DW 41.600 usec
DE 6.50 usec
TE 298.2 K
D1 1.00000000 sec
TD0 1
======== CHANNEL f1 ========
SFO1 600.1537062 MHz
NUC1 1H
P1 10.90 usec
PLW1 22.60000038 W
F2 - Processing parameters
SI 65536
SF 600.1500266 MHz
WDW EM
SSB 0
LB 0.30 Hz
GB 0
PC 1.00
ppm

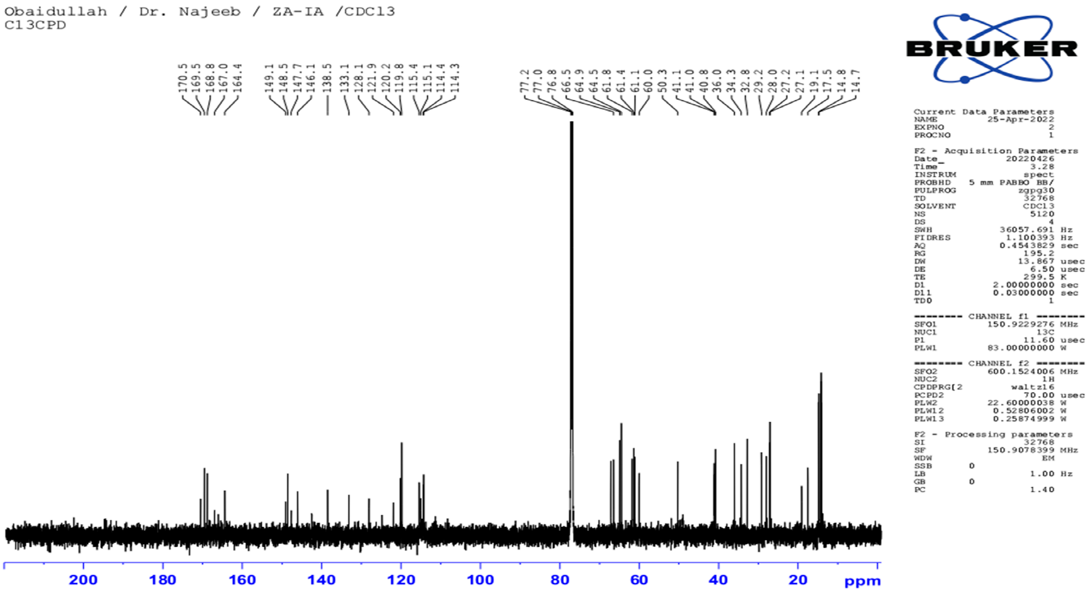

Figure S3: ^{1}H-NMR and ^{13}C-NMR spectra of compound **1**

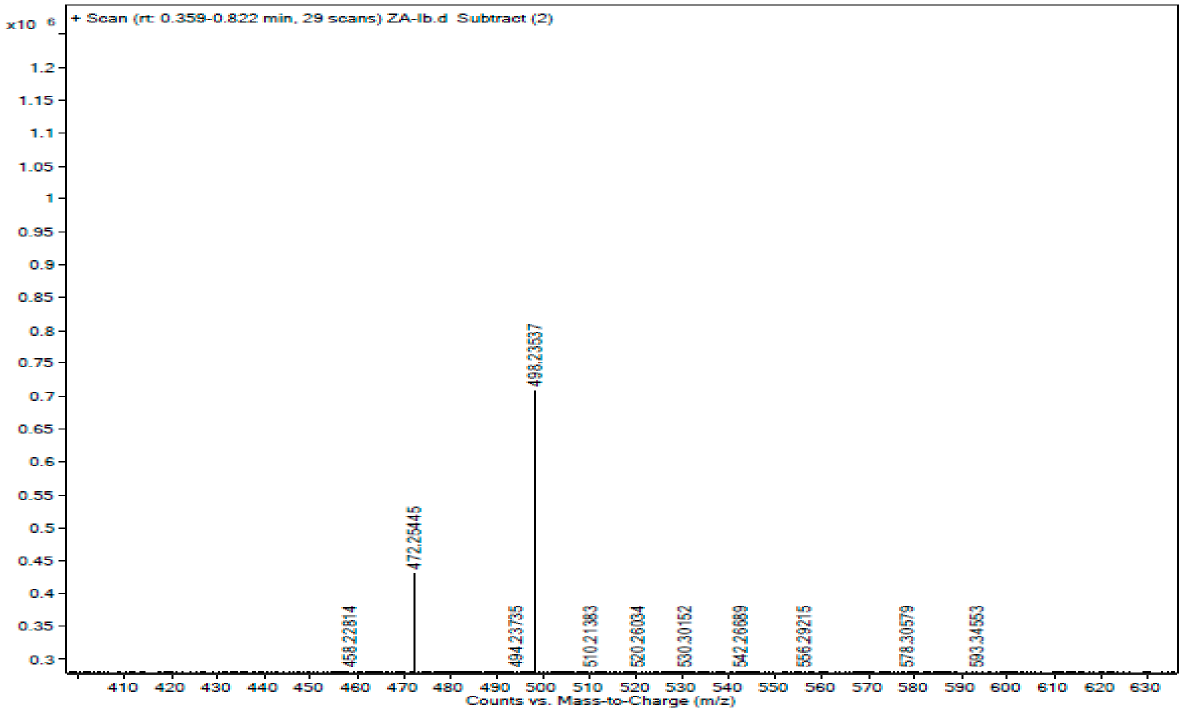

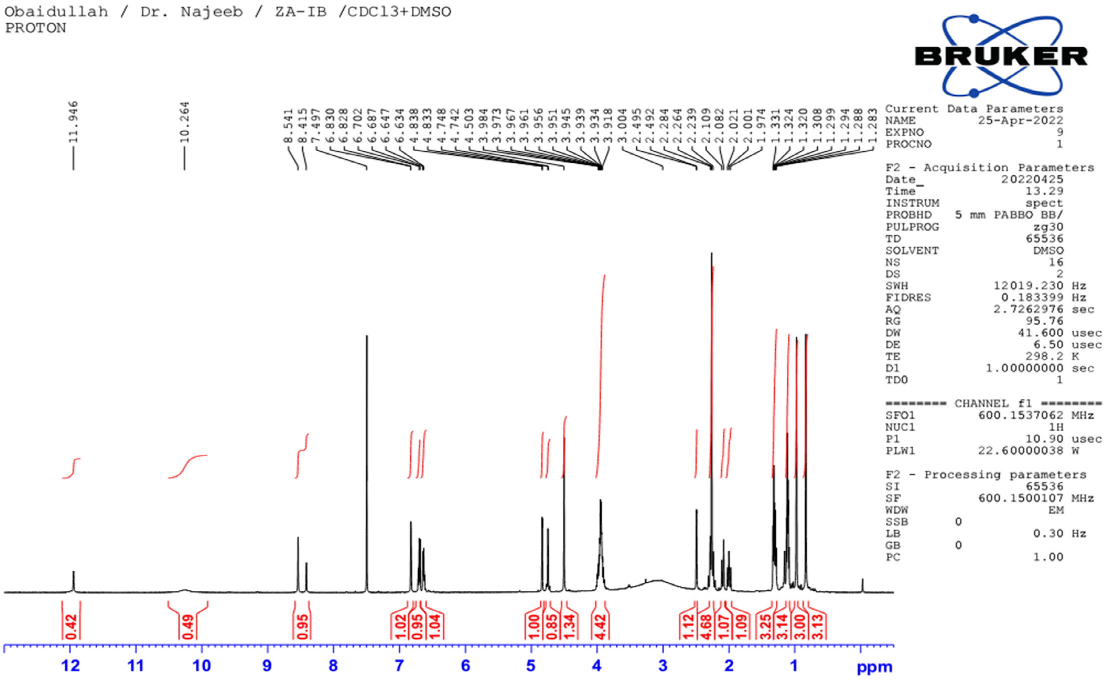

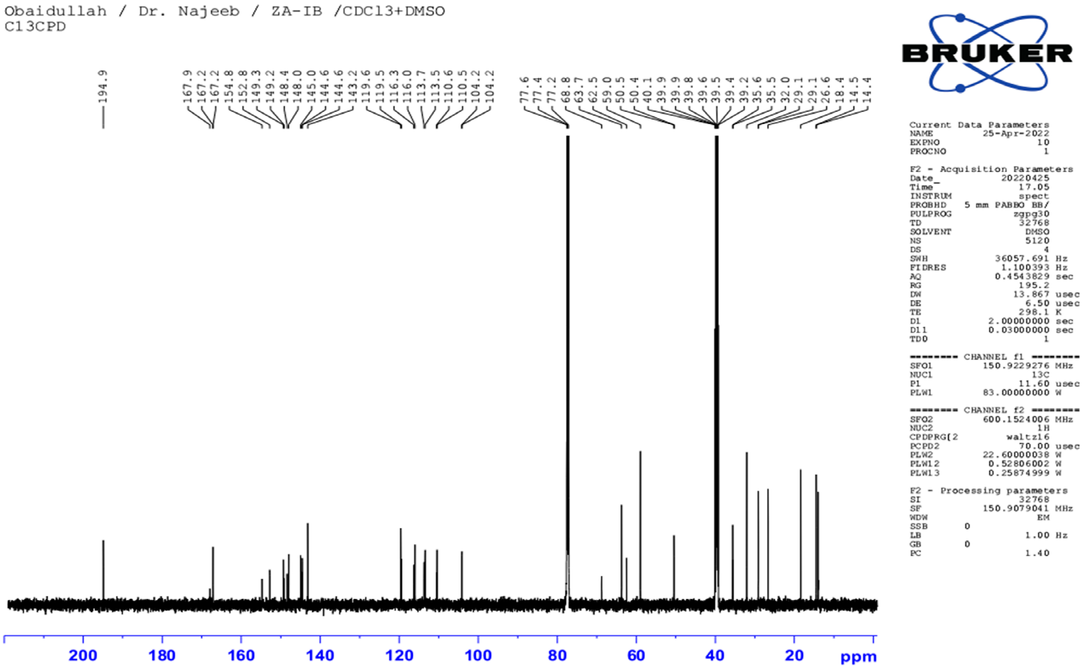

Figure S4: ^{1}H-NMR and ^{13}C-NMR spectra of compound **2**

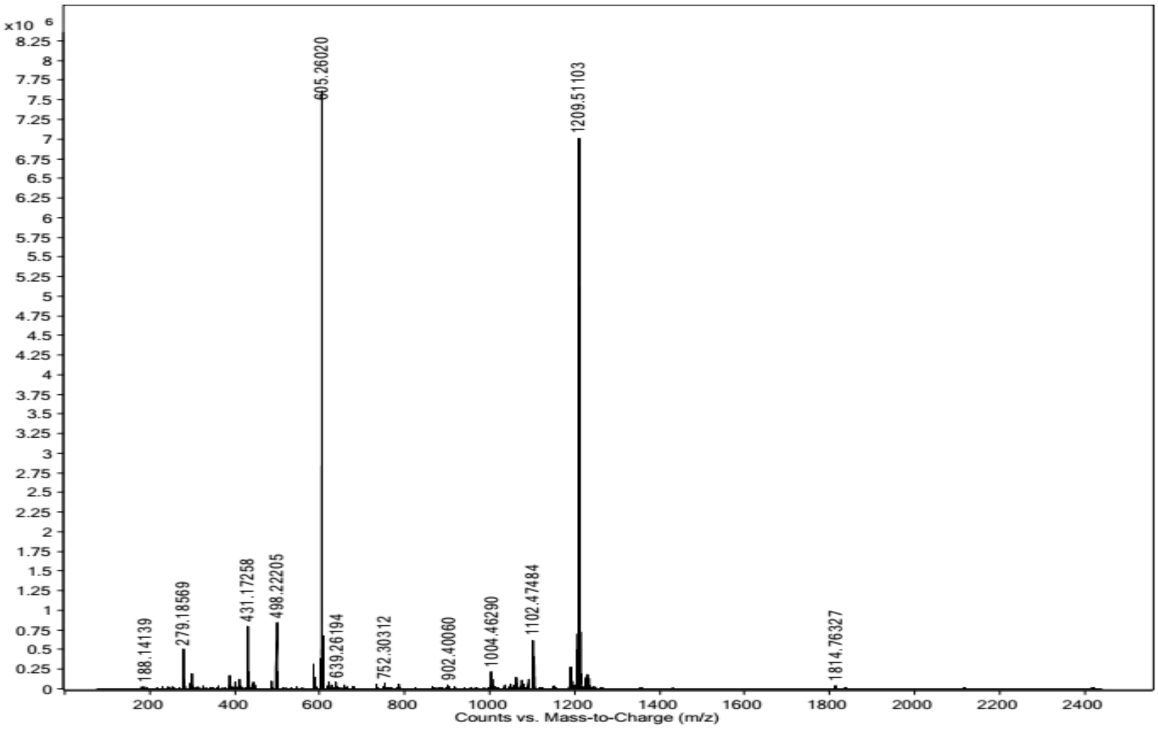
x10 6
605.26020
1209.51103
188.14139
279.18569
431.17258
498.22205
639.26194
752.30312
902.40060
1004.46290
1102.47484
1814.76327
Counts vs. Mass-to-Charge (m/z)

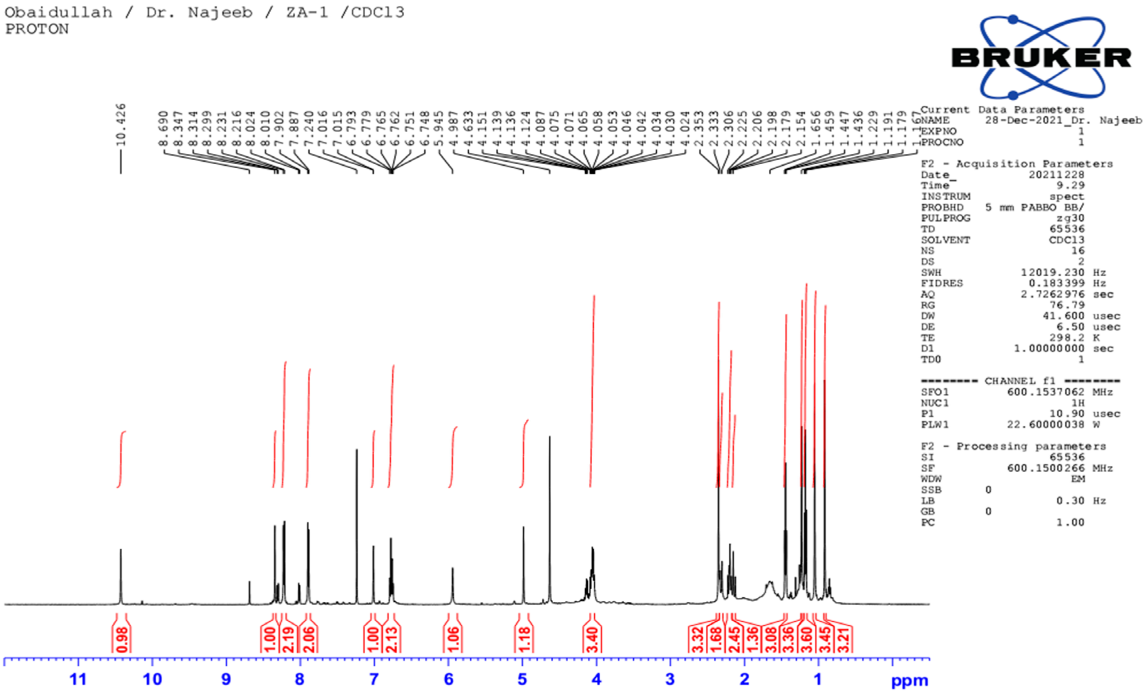
Obaidullah / Dr. Najeeb / ZA-1 /CDCl3
PROTON
BRUKER
Current Data Parameters
NAME 28-Dec-2021_Dr. Najeeb
EXPNO 1
PROCNO 1
F2 - Acquisition Parameters
Date_ 20211228
Time 9.29
INSTRUM spect
PROBHD 5 mm PABBO BB/
PULPROG zg30
TD 65536
SOLVENT CDCl3
NS 16
DS 2
SWH 12019.230 Hz
FIDRES 0.183399 Hz
AQ 2.7262976 sec
RG 76.79
DW 41.600 usec
DE 6.50 usec
TE 298.2 K
D1 1.00000000 sec
TD0 1
======== CHANNEL f1 ========
SFO1 600.1537062 MHz
NUC1 1H
P1 10.90 usec
PLW1 22.60000038 W
F2 - Processing parameters
SI 65536
SF 600.1500266 MHz
WDW EM
SSB 0
LB 0.30 Hz
GB 0
PC 1.00
ppm

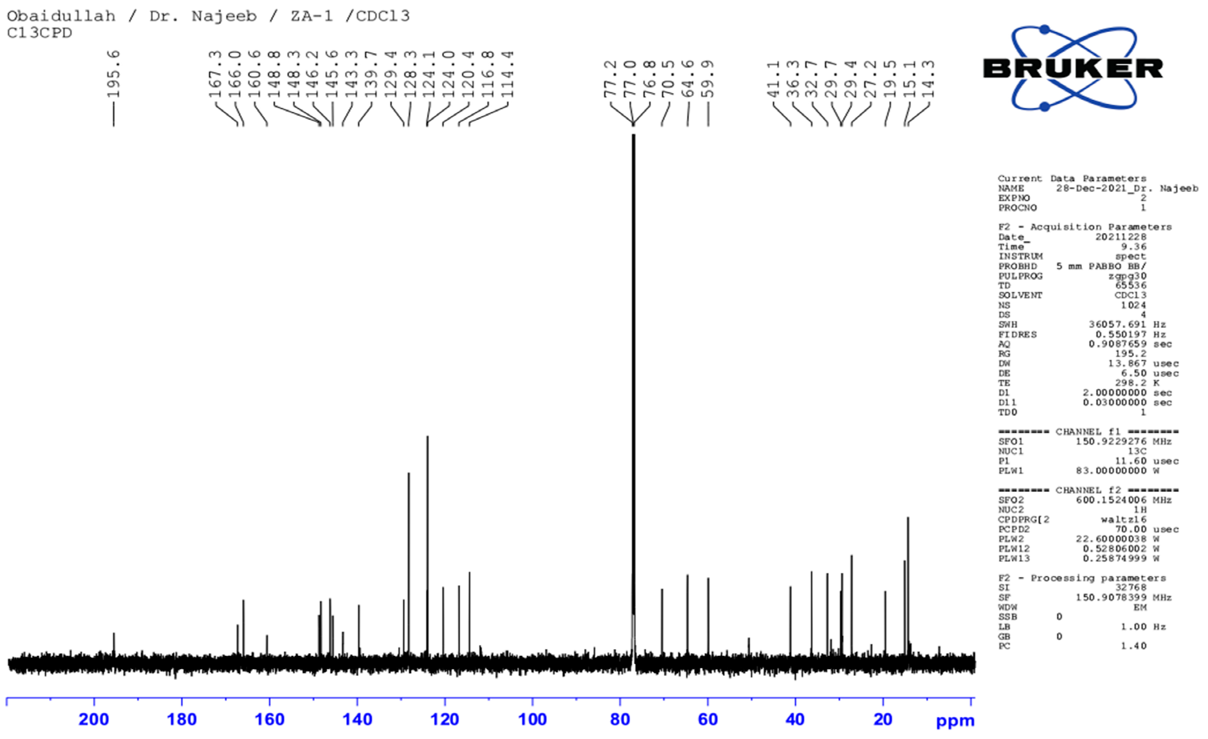

Figure S5: Mass, ^{1}H-NMR, and ^{13}C-NMR spectra of compound **3**

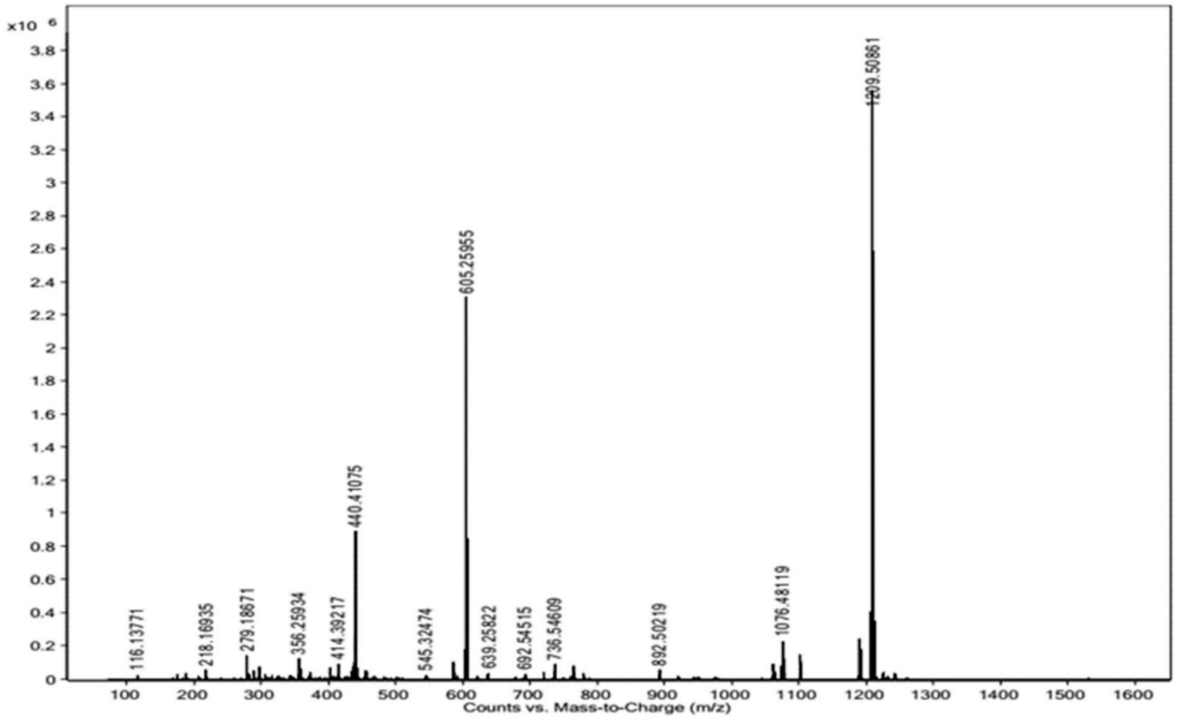

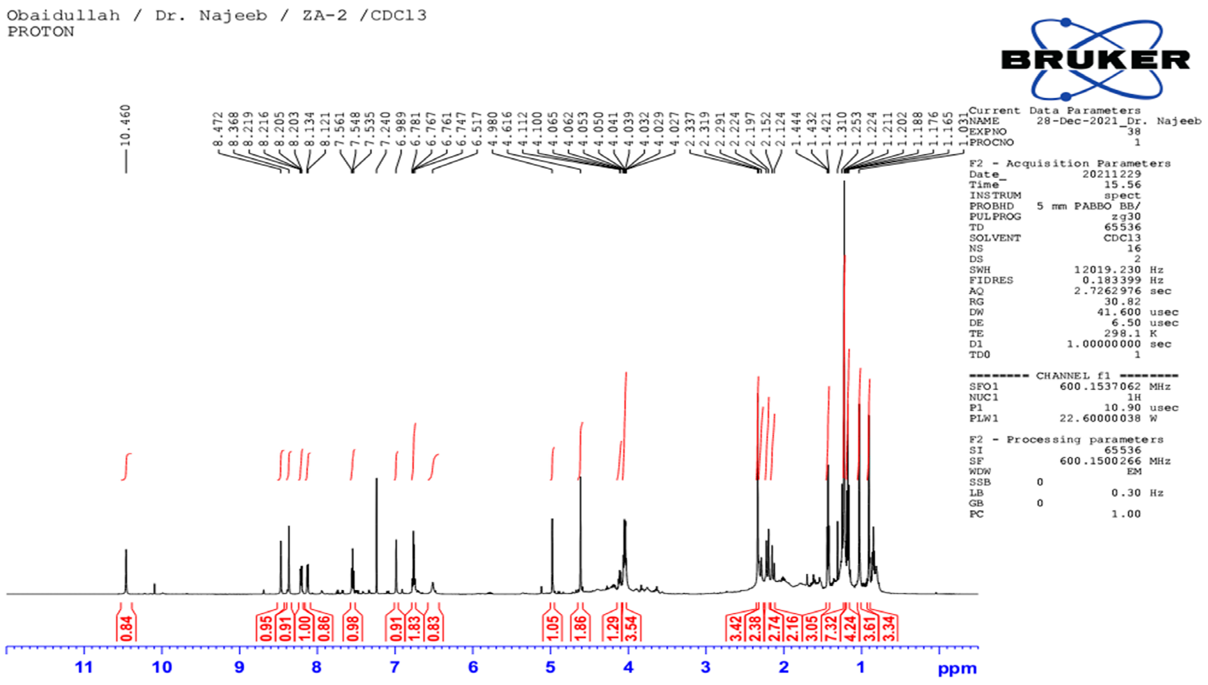

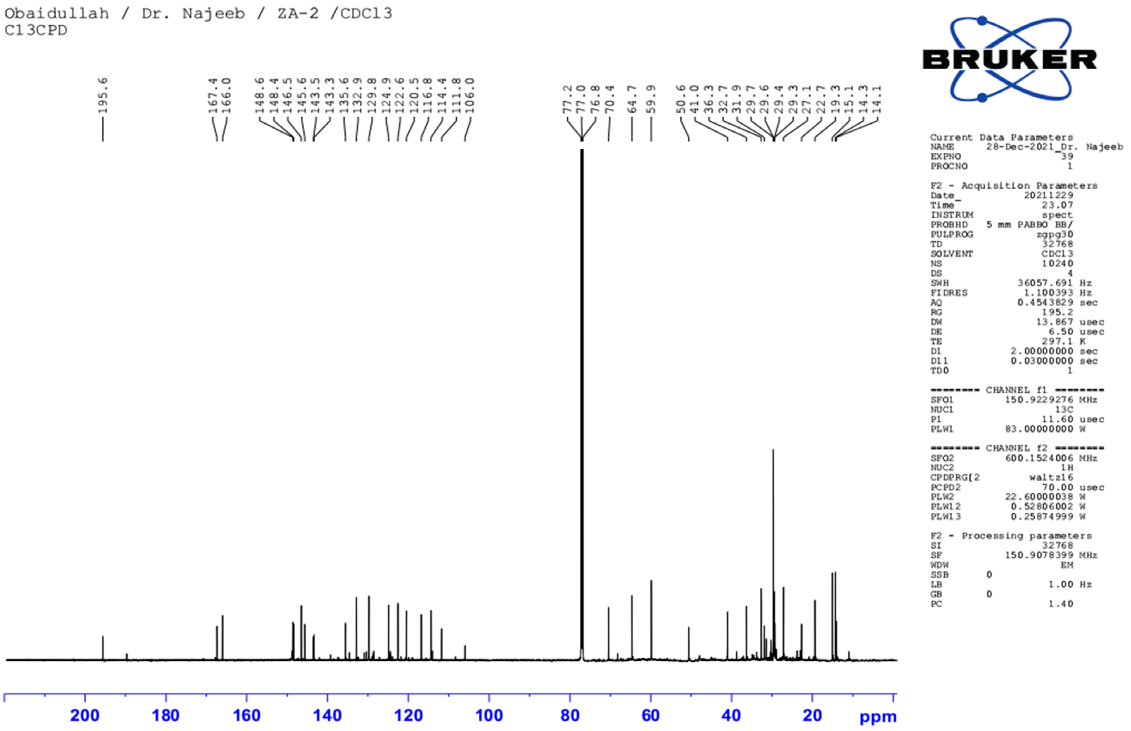

Figure S6: Mass, ^{1}H-NMR, and ^{13}C-NMR spectra of compound **4**

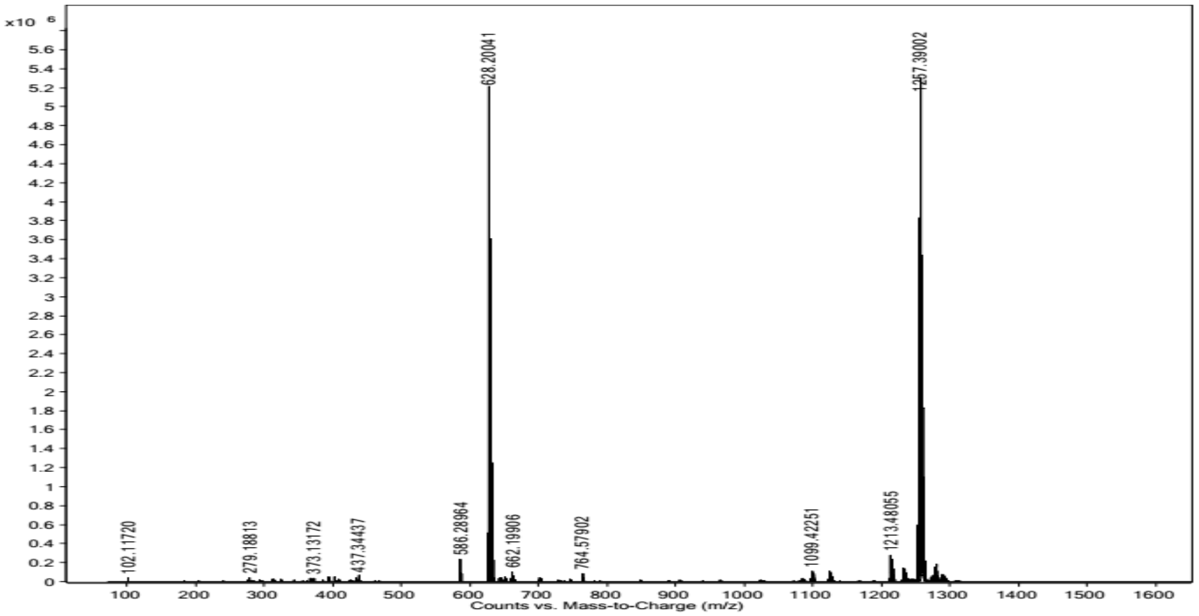
x10 6
628.20041
1257.39002
102.11720
279.18813
373.13172
437.34437
586.28964
662.19906
764.57902
1099.42251
1213.48055
Counts vs. Mass-to-Charge (m/z)

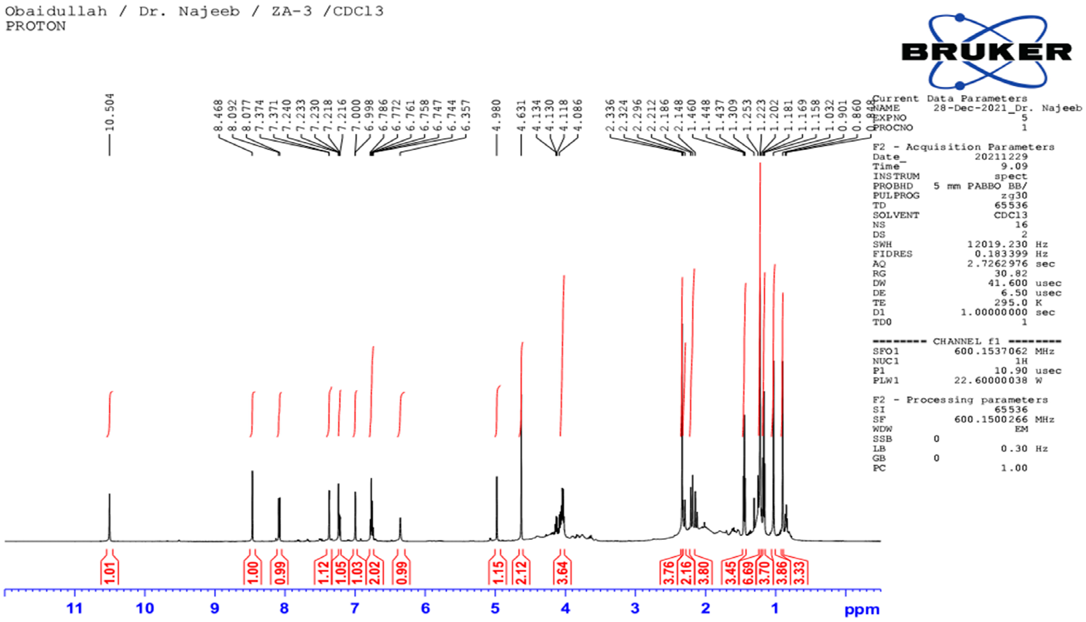
Obaidullah / Dr. Najeeb / ZA-3 /CDCl3
PROTON
10.504
8.468
8.092
8.077
7.374
7.371
7.240
7.233
7.230
7.218
7.216
7.000
6.998
6.786
6.772
6.761
6.758
6.747
6.744
6.357
4.980
4.631
4.134
4.130
4.118
4.086
2.336
2.324
2.296
2.212
2.186
2.148
1.460
1.448
1.437
1.309
1.253
1.223
1.202
1.181
1.169
1.158
1.032
0.901
0.860
Current Data Parameters
NAME 28-Dec-2021_Dr. Najeeb
EXPNO 5
PROCNO 1
F2 - Acquisition Parameters
Date_ 20211229
Time 9.09
INSTRUM spect
PROBHD 5 mm PABBO BB/
PULPROG zg30
TD 65536
SOLVENT CDCl3
NS 16
DS 2
SWH 12019.230 Hz
FIDRES 0.183399 Hz
AQ 2.7262976 sec
RG 30.82
DW 41.600 usec
DE 6.50 usec
TE 295.0 K
D1 1.00000000 sec
TD0 1
======== CHANNEL f1 ========
SFO1 600.1537062 MHz
NUC1 1H
P1 10.90 usec
PLW1 22.60000038 W
F2 - Processing parameters
SI 65536
SF 600.1500266 MHz
WDW EM
SSB 0
LB 0.30 Hz
GB 0
PC 1.00
1.01
1.00
0.99
1.12
1.05
1.03
2.02
0.99
1.15
2.12
3.64
3.76
2.16
3.80
3.45
6.69
3.70
3.86
3.33
ppm

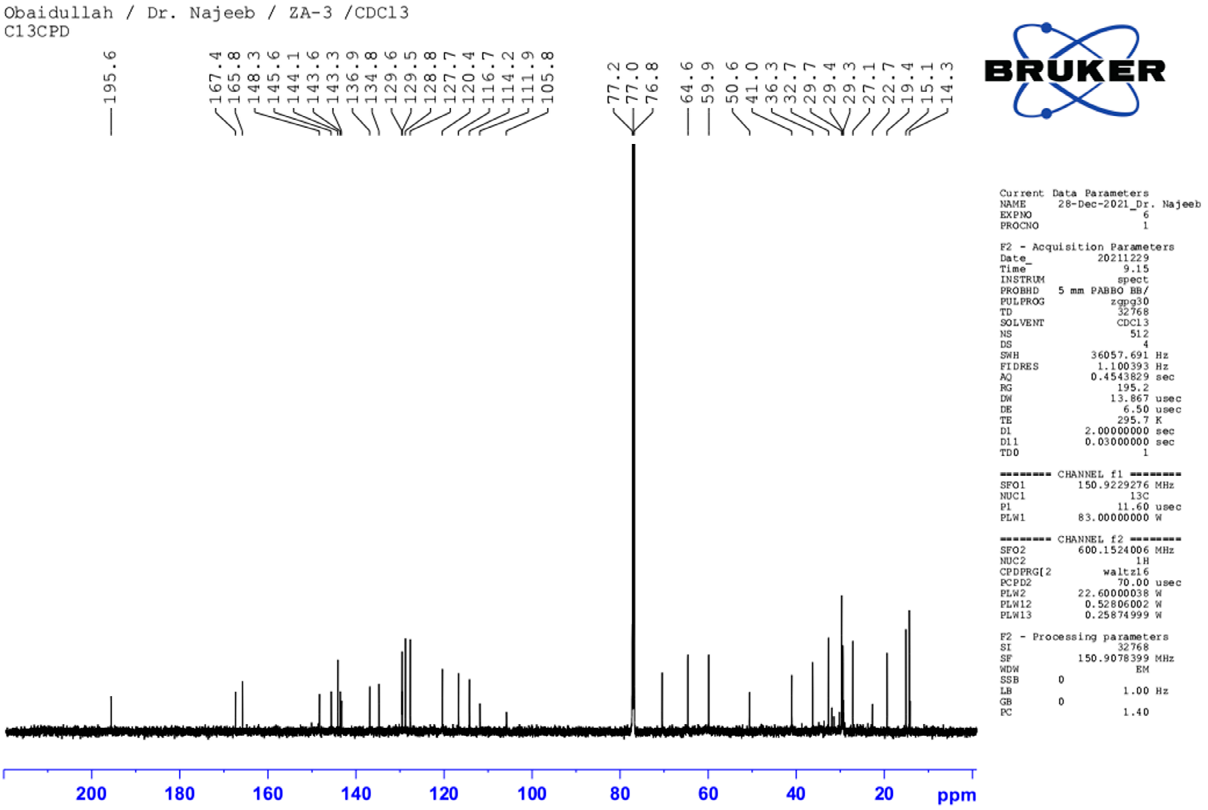

Figure S7: Mass, ^{1}H- and ^{13}C-NMR spectra of compound **5**

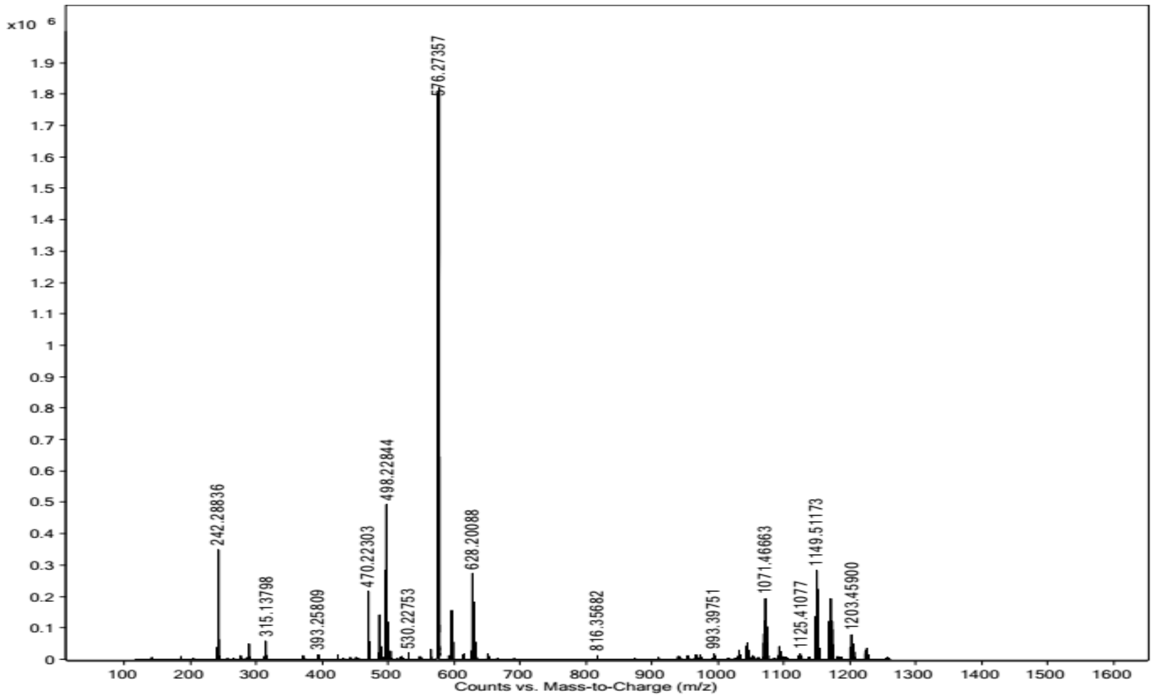

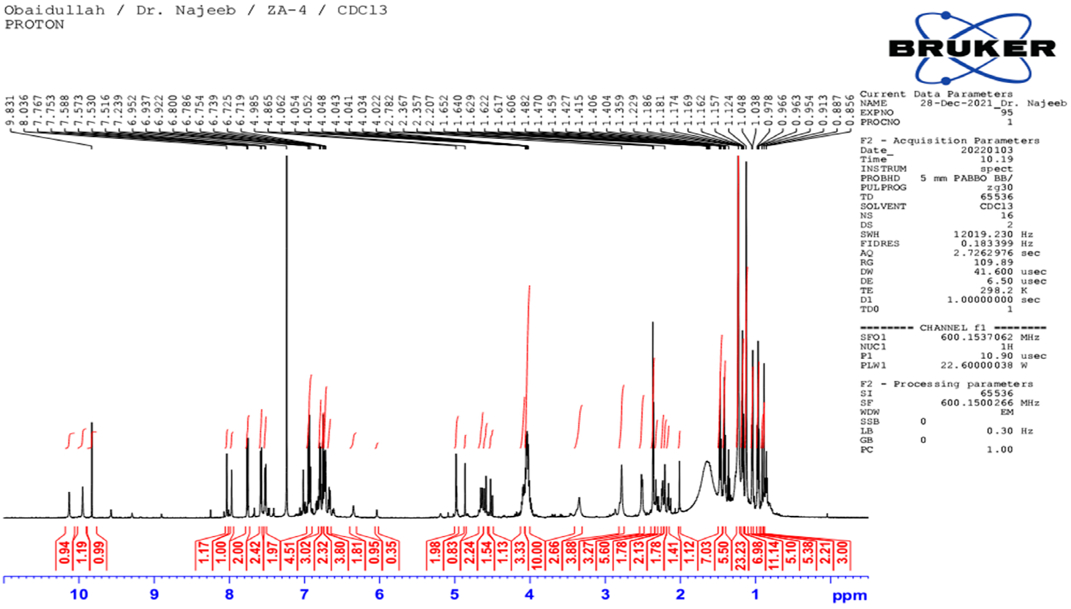

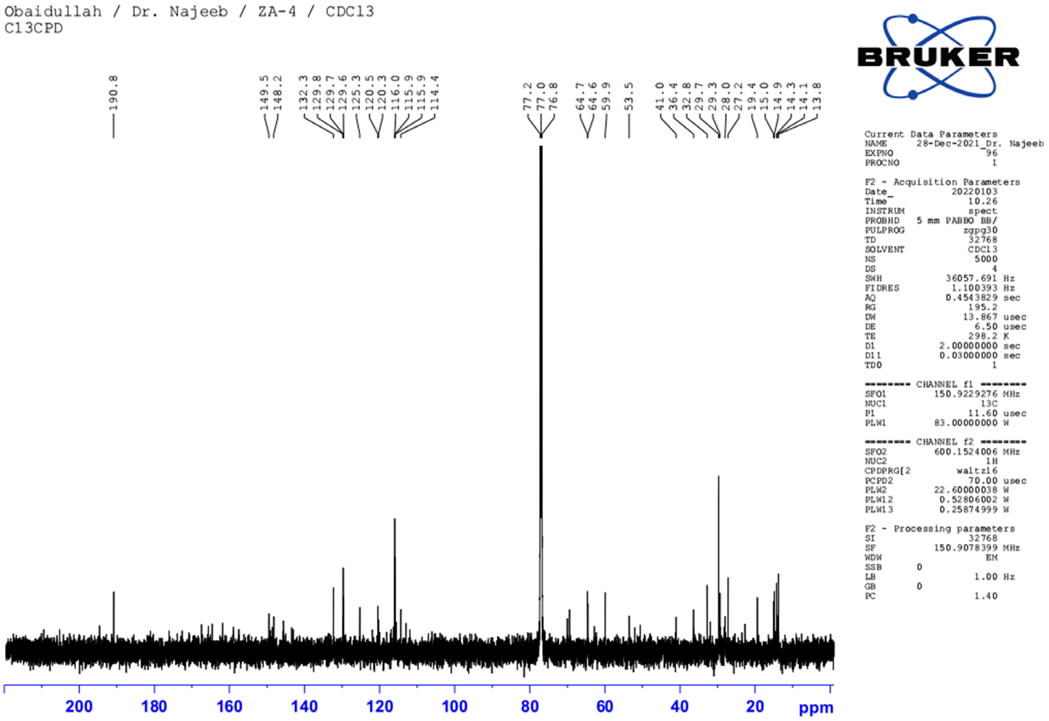

Figure S8: Mass, ^{1}H- and ^{13}C-NMR spectra of compound **6**

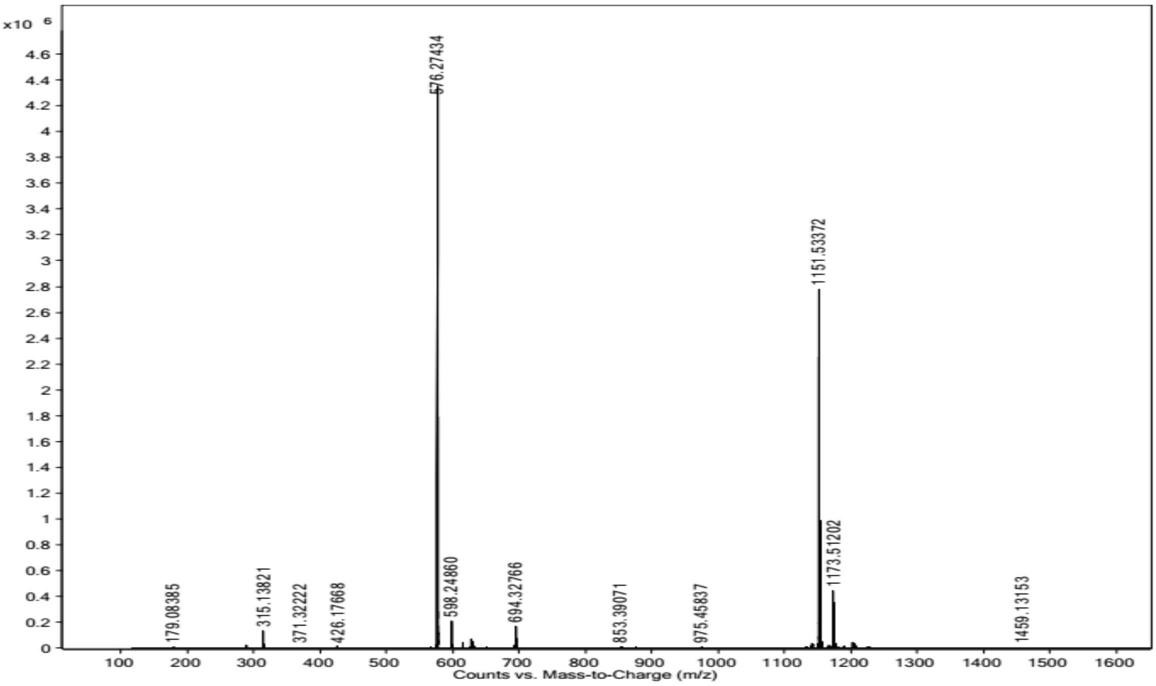
x10 6
576.27434
1151.53372
1173.51202
179.08385
315.13821
371.32222
426.17668
598.24860
694.32766
853.39071
975.45837
1459.13153
Counts vs. Mass-to-Charge (m/z)

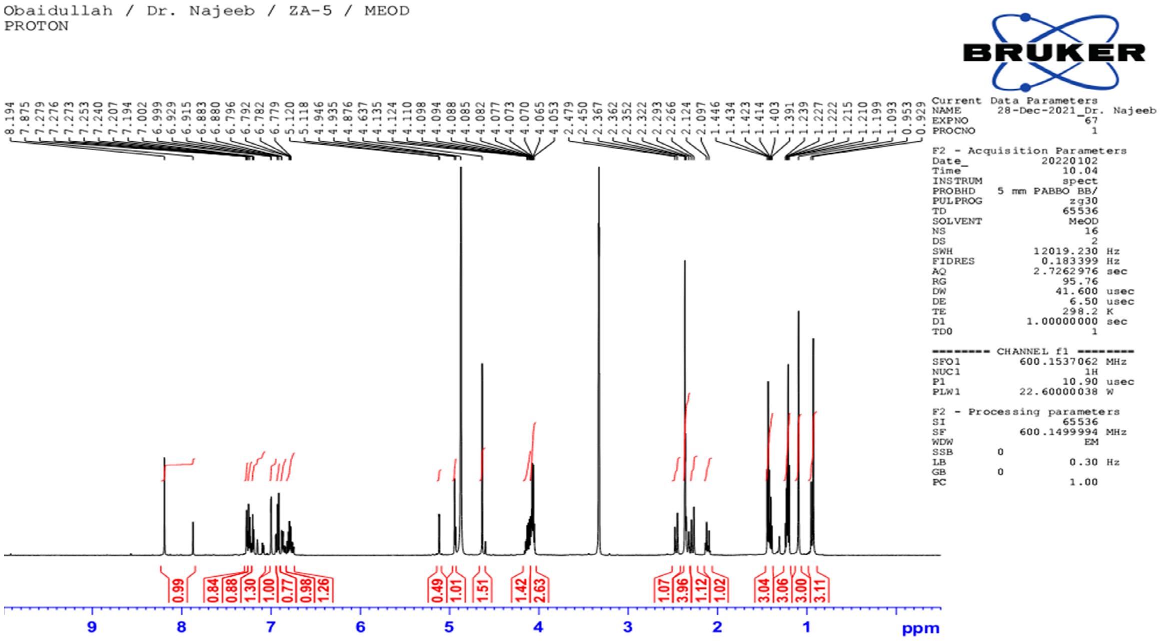
Obaidullah / Dr. Najeeb / ZA-5 / MEOD
PROTON
BRUKER
Current Data Parameters
NAME 28-Dec-2021_Dr. Najeeb
EXPNO 67
PROCNO 1
F2 - Acquisition Parameters
Date_ 20220102
Time 10.04
INSTRUM spect
PROBHD 5 mm PABBO BB/
PULPROG zg30
TD 65536
SOLVENT MeOD
NS 16
DS 2
SWH 12019.230 Hz
FIDRES 0.183399 Hz
AQ 2.7262976 sec
RG 95.76
DW 41.600 usec
DE 6.50 usec
TE 298.2 K
D1 1.00000000 sec
TD0 1
======== CHANNEL f1 ========
SFO1 600.1537062 MHz
NUC1 1H
P1 10.90 usec
PLW1 22.60000038 W
F2 - Processing parameters
SI 65536
SF 600.1499994 MHz
WDW EM
SSB 0
LB 0.30 Hz
GB 0
PC 1.00
ppm

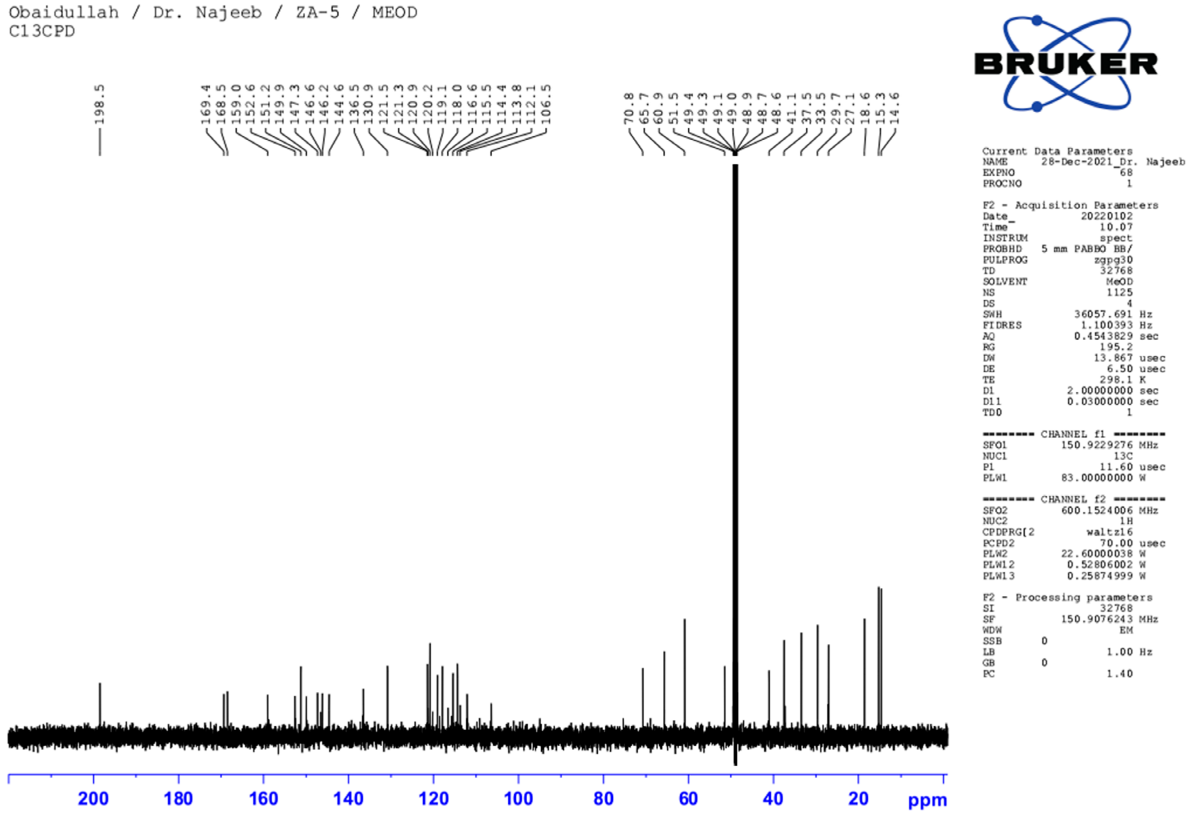

Figure S9: Mass, ^{1}H- and ^{13}C-NMR spectra of compound **7**

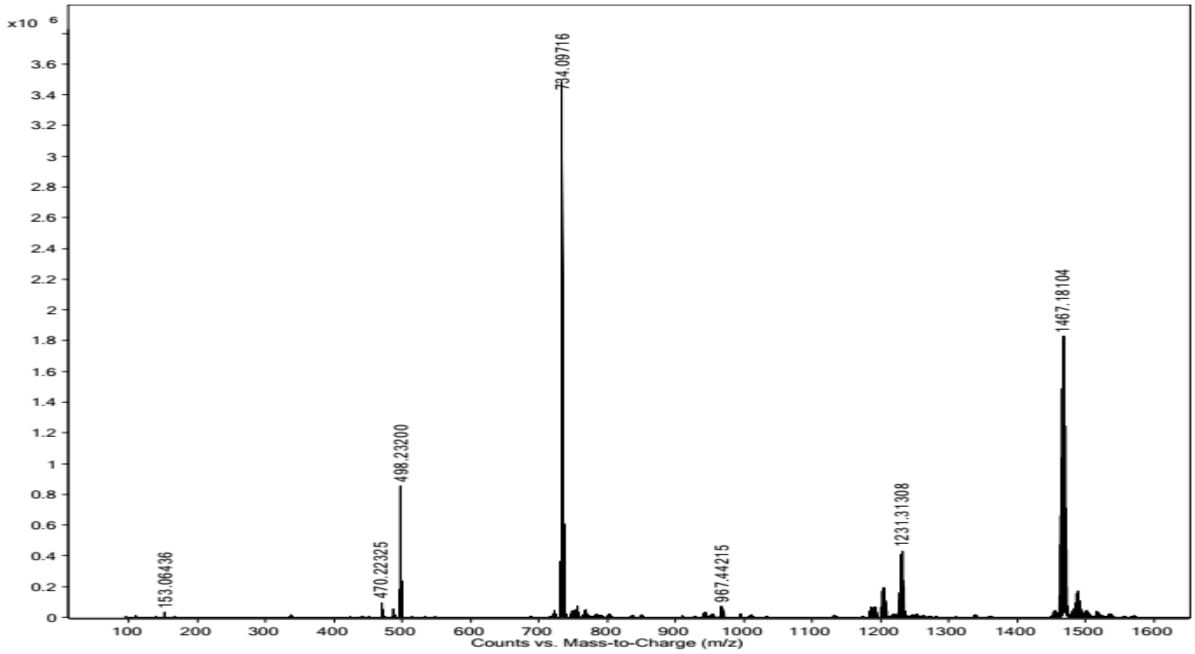

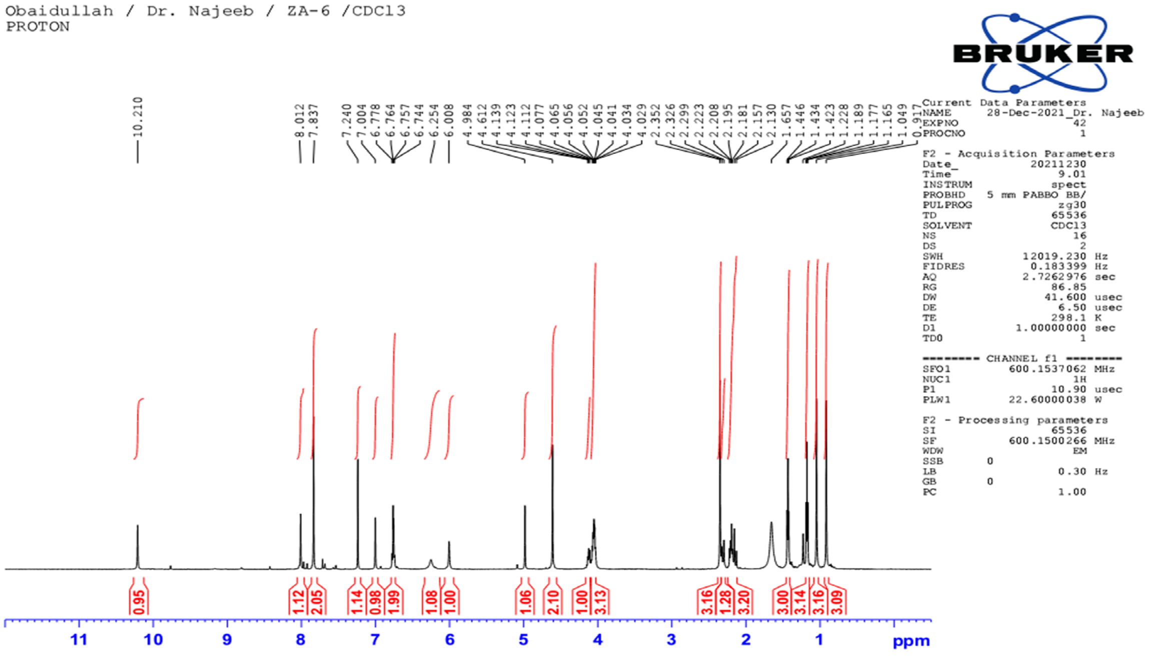

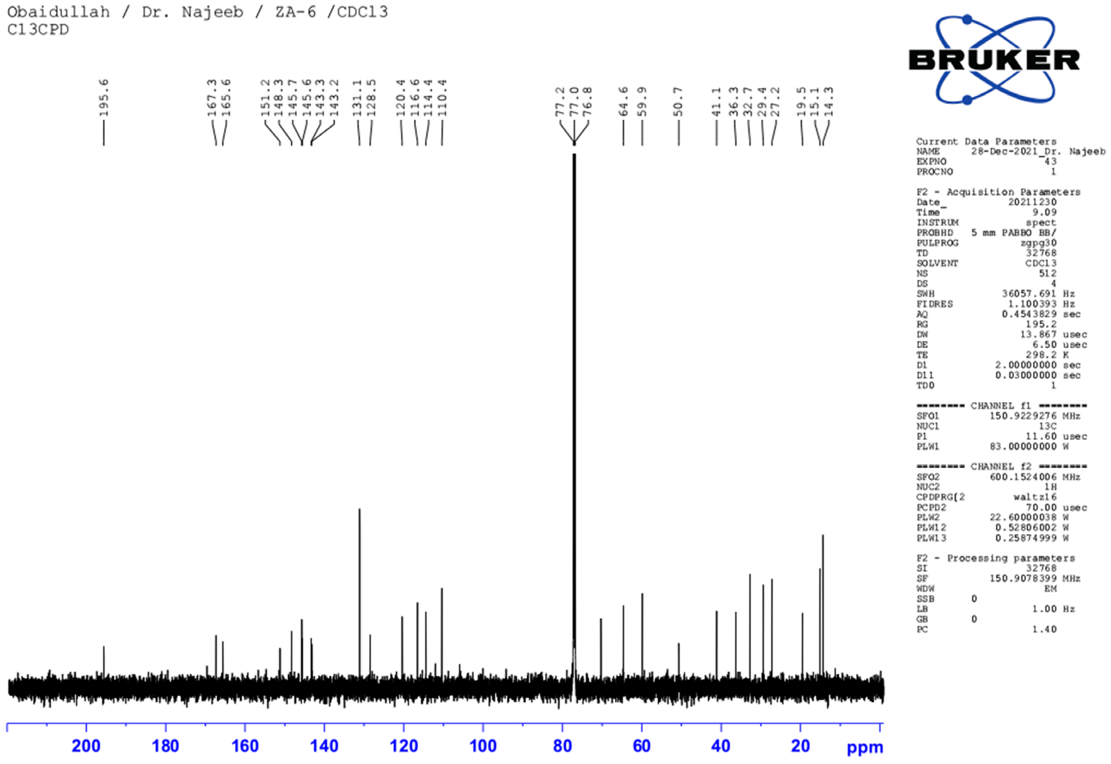

Figure S10: Mass, ^{1}H- and ^{13}C-NMR spectra of compound **8**

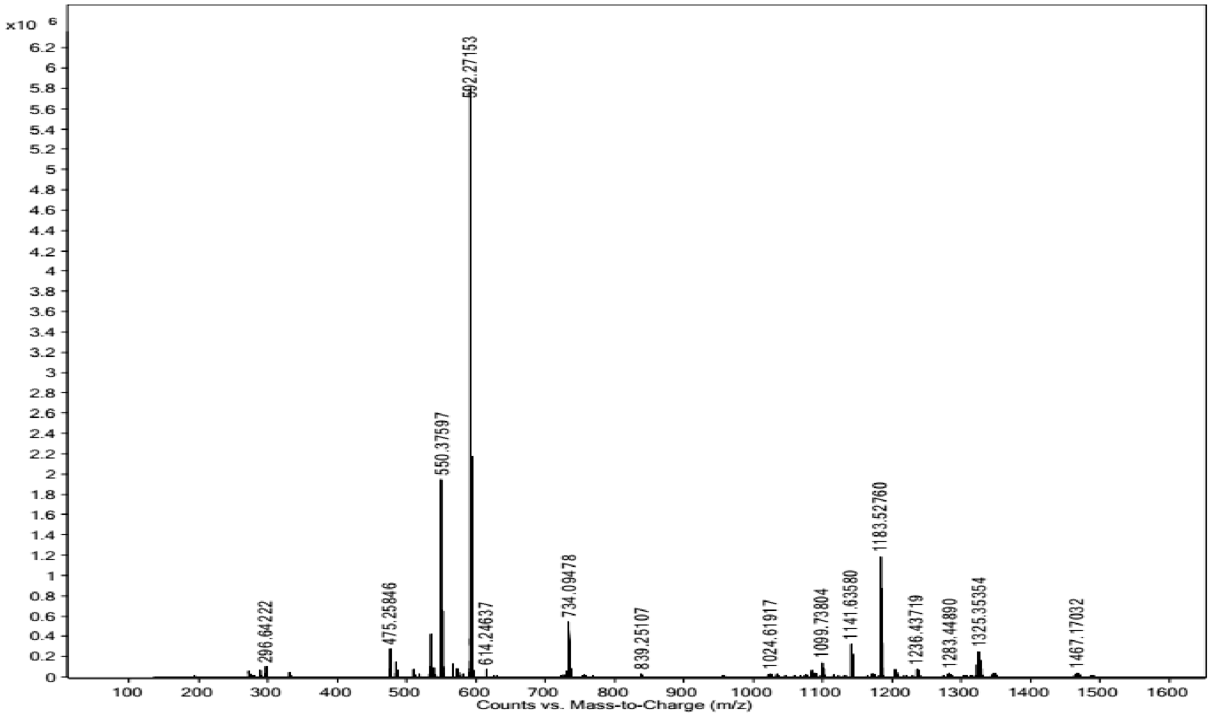
x10 6
592.27153
550.37597
475.25846
296.64222
614.24637
734.09478
839.25107
1024.61917
1099.73804
1141.63580
1183.52760
1236.43719
1283.44890
1325.35354
1467.17032
Counts vs. Mass-to-Charge (m/z)

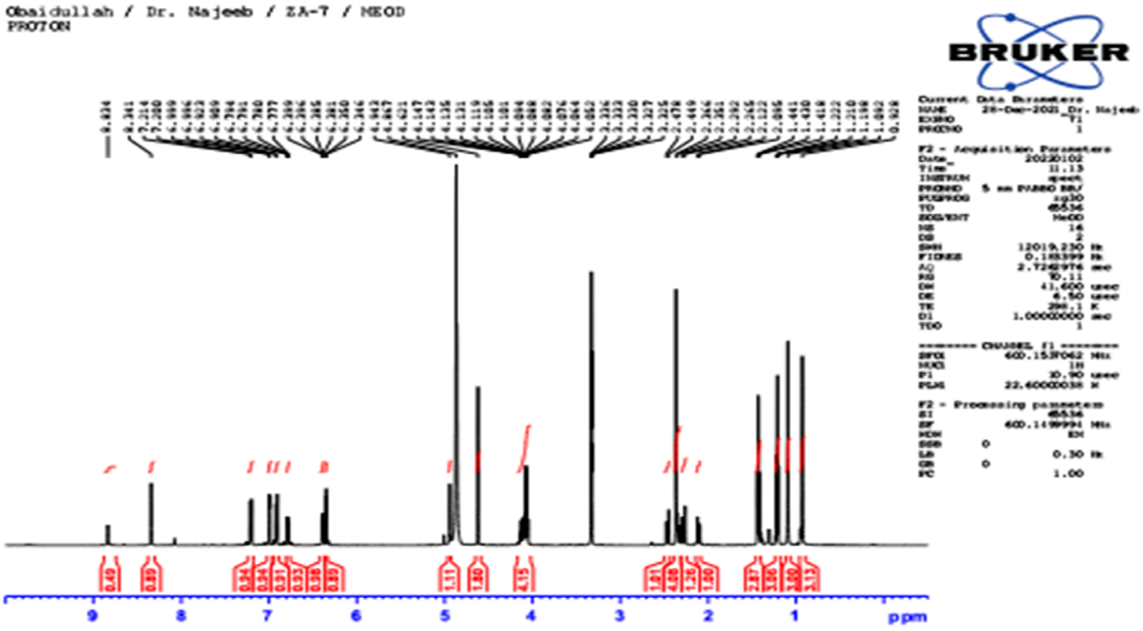
Obaidullah / Dr. Najeeb / ZA-7 / MEOD
PROTON
BRUKER
ppm

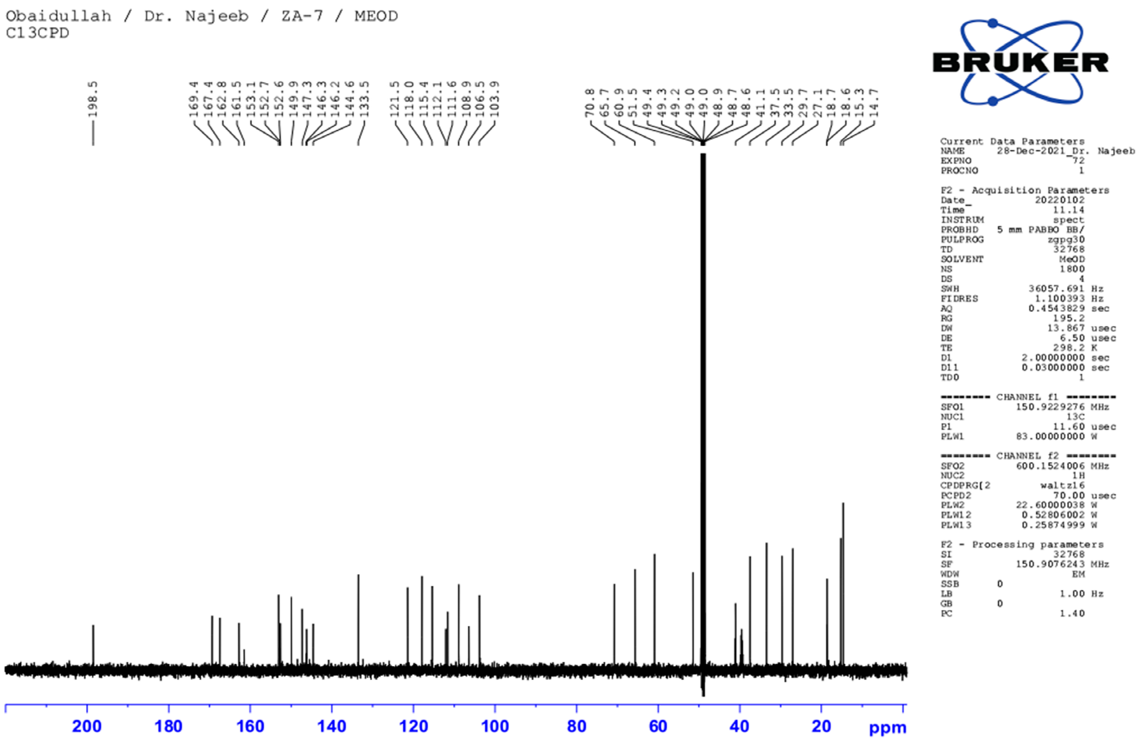

Figure S11: Mass, ^{1}H- and ^{13}C-NMR spectra of compound **9**

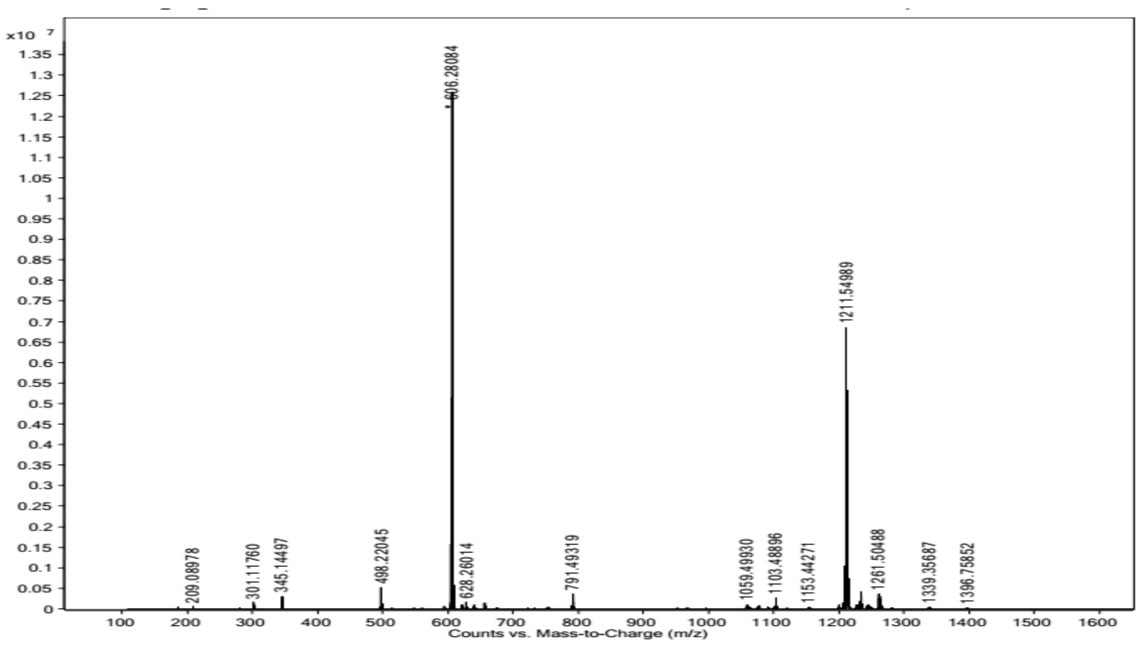

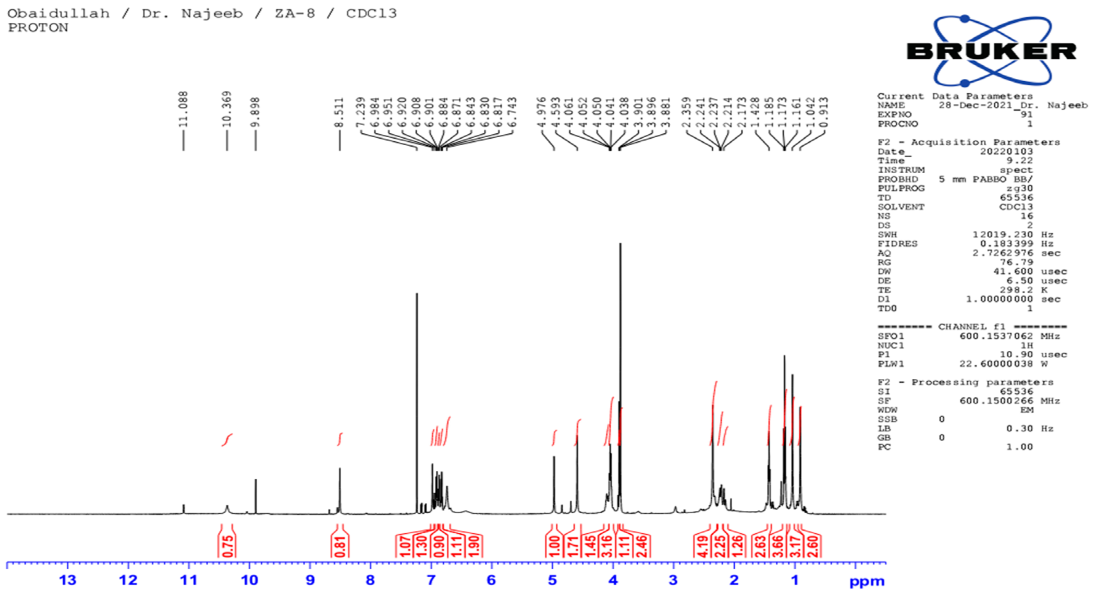

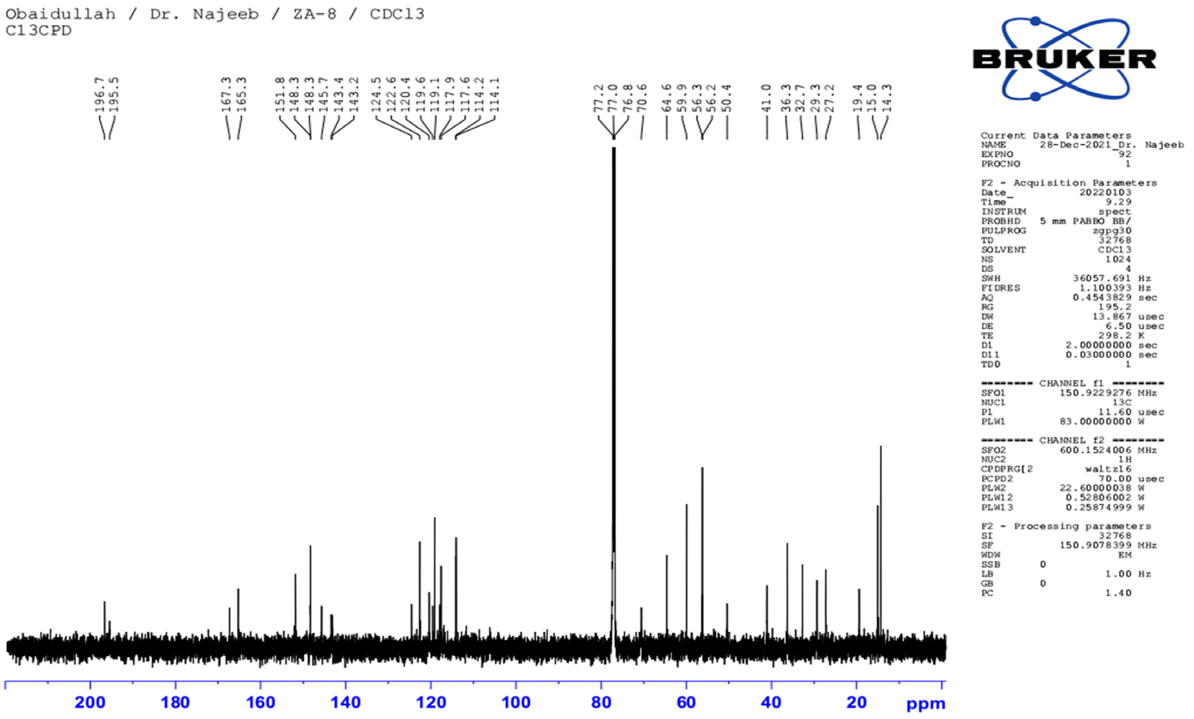

Figure S12: Mass, ^{1}H- and ^{13}C-NMR spectra of compound **10**

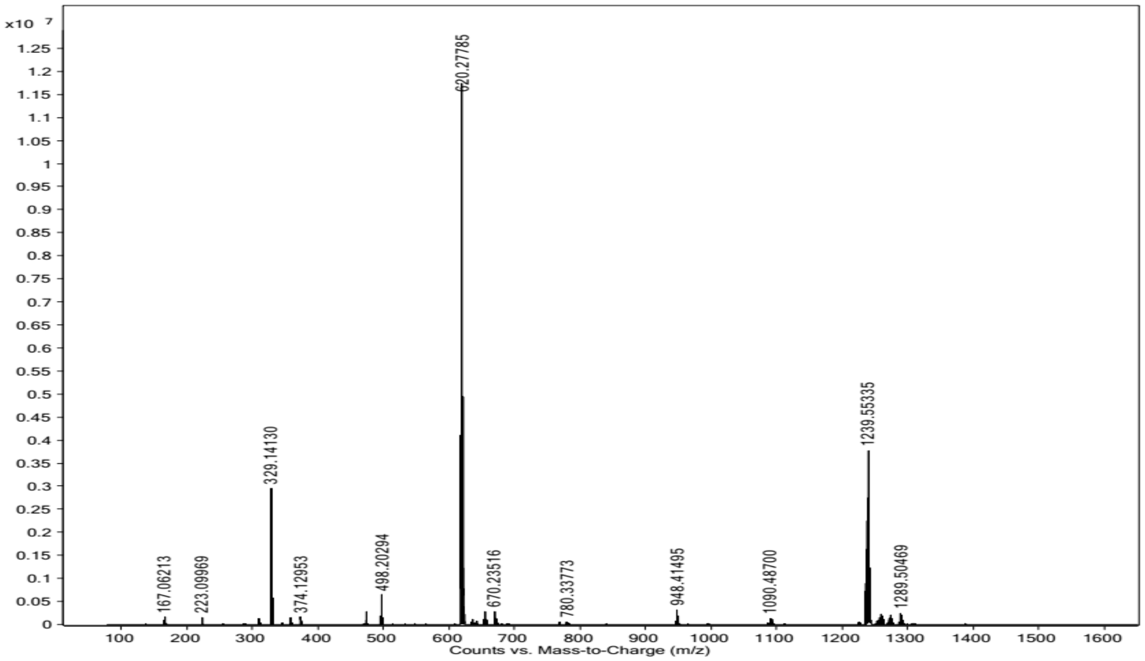
x10 7
620.27785
329.14130
1239.55335
167.06213
223.09969
374.12953
498.20294
670.23516
780.33773
948.41495
1090.48700
1289.50469
Counts vs. Mass-to-Charge (m/z)

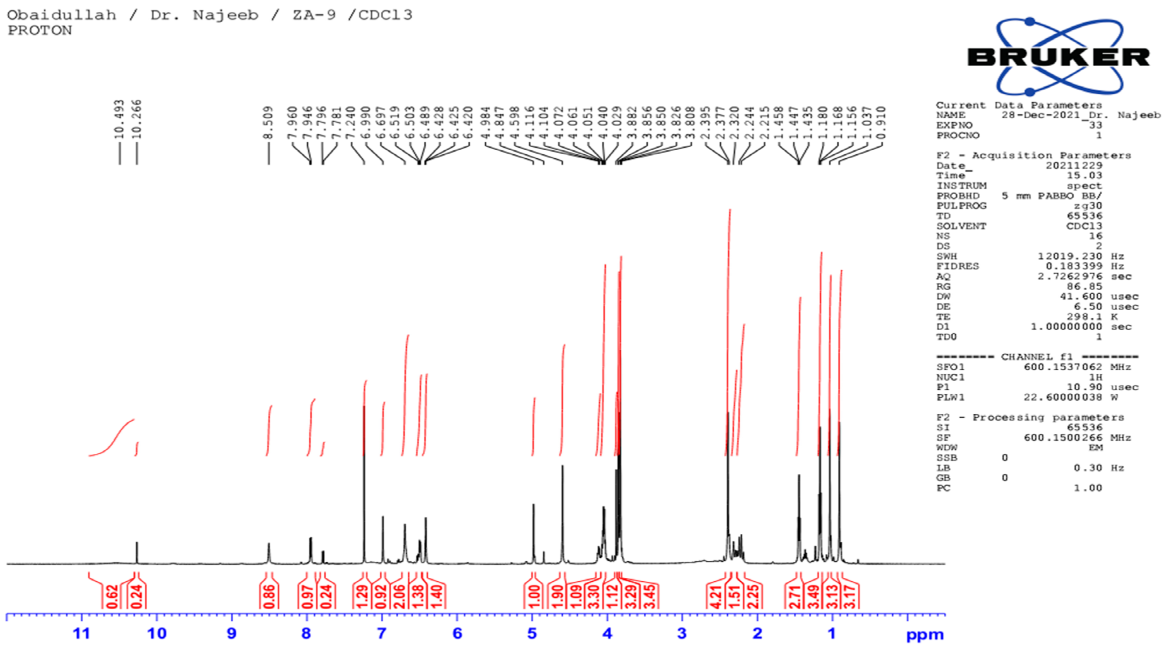
Obaidullah / Dr. Najeeb / ZA-9 /CDCl3
PROTON
BRUKER
ppm

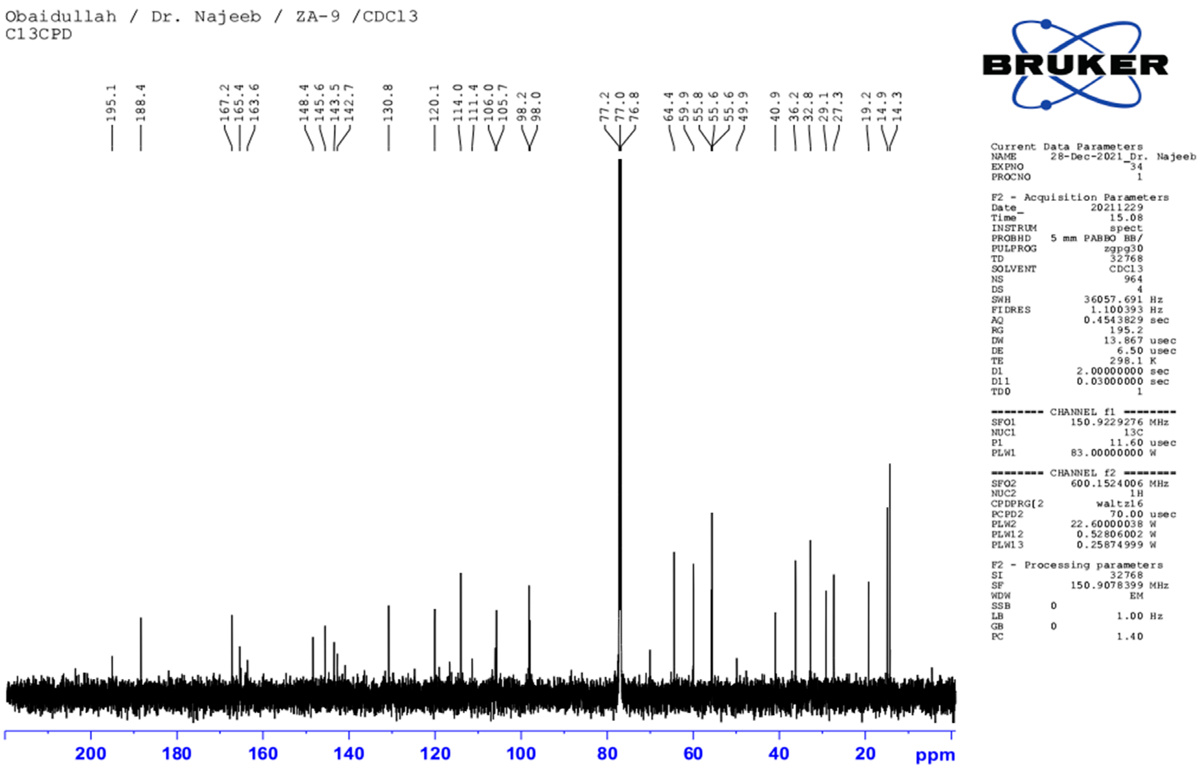

Figure S13: Mass, ^{1}H- and ^{13}C-NMR spectra of compound **11**

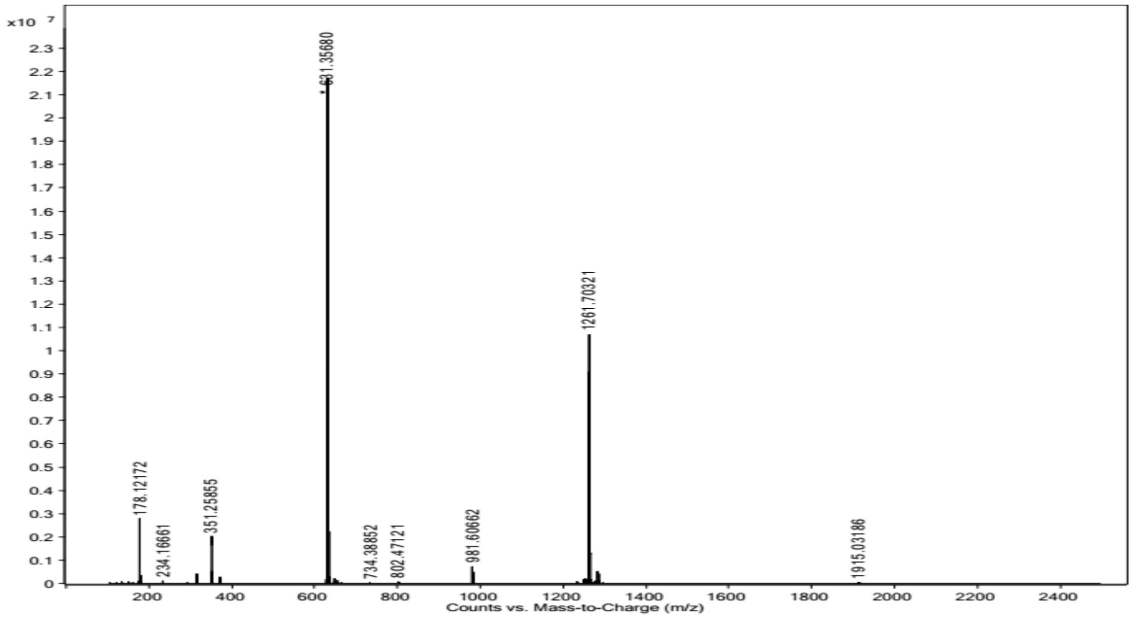

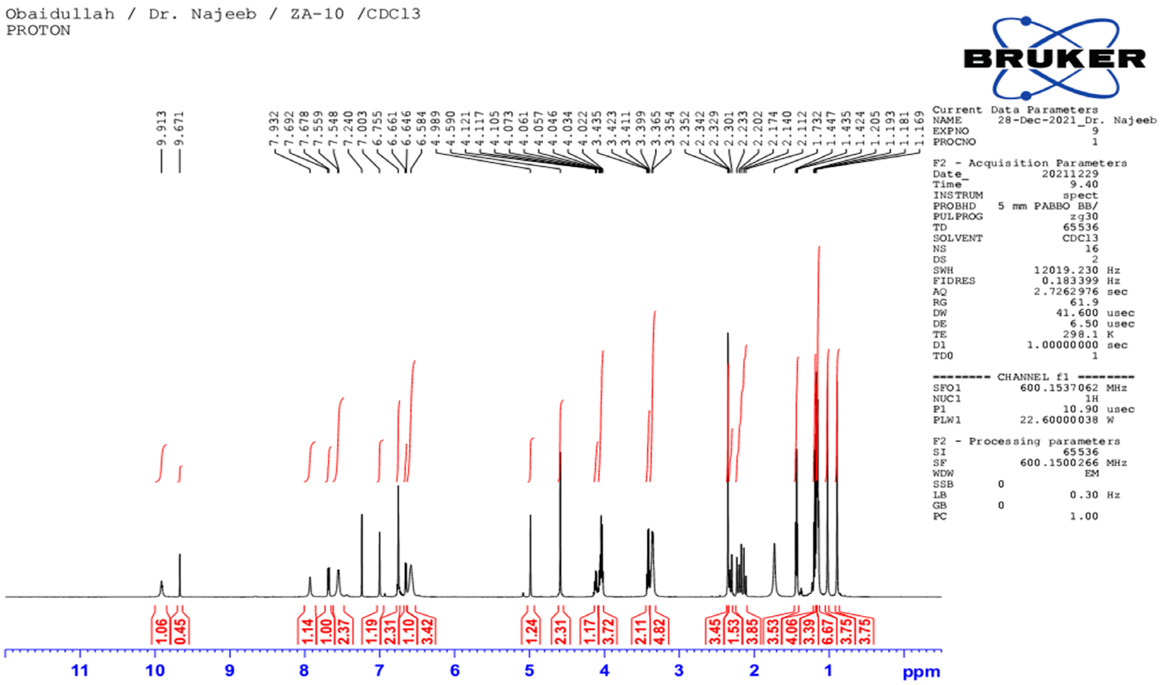

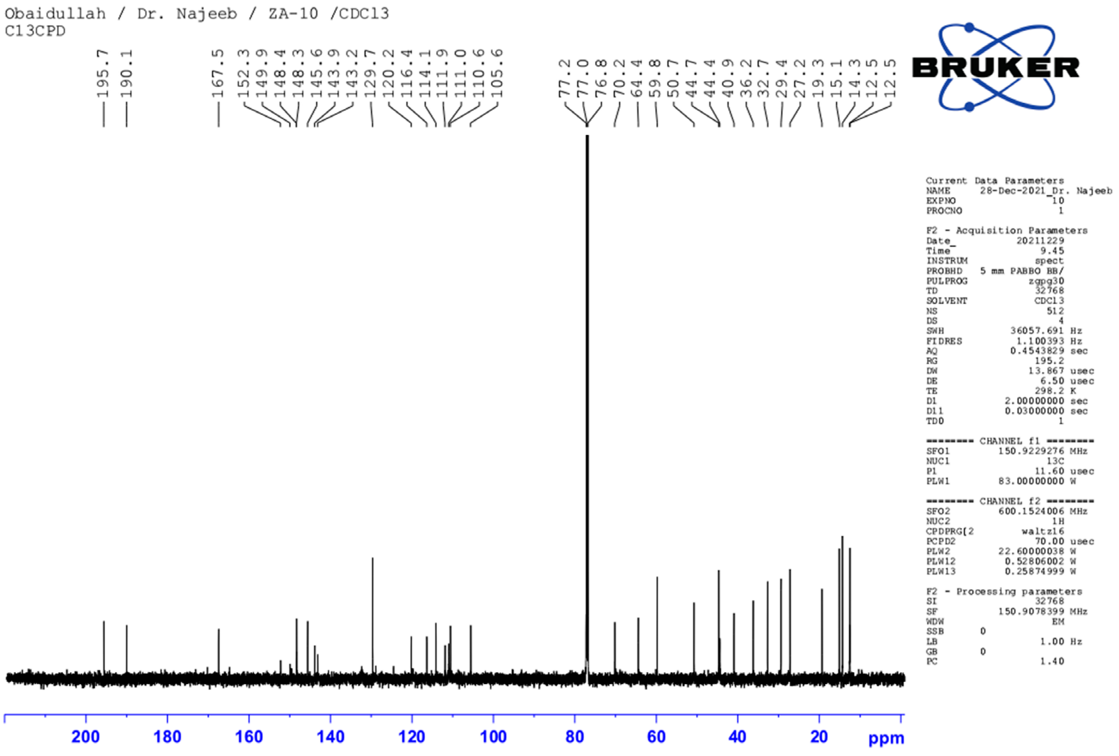

Figure S14: Mass, ^{1}H- and ^{13}C-NMR spectra of compound **12**

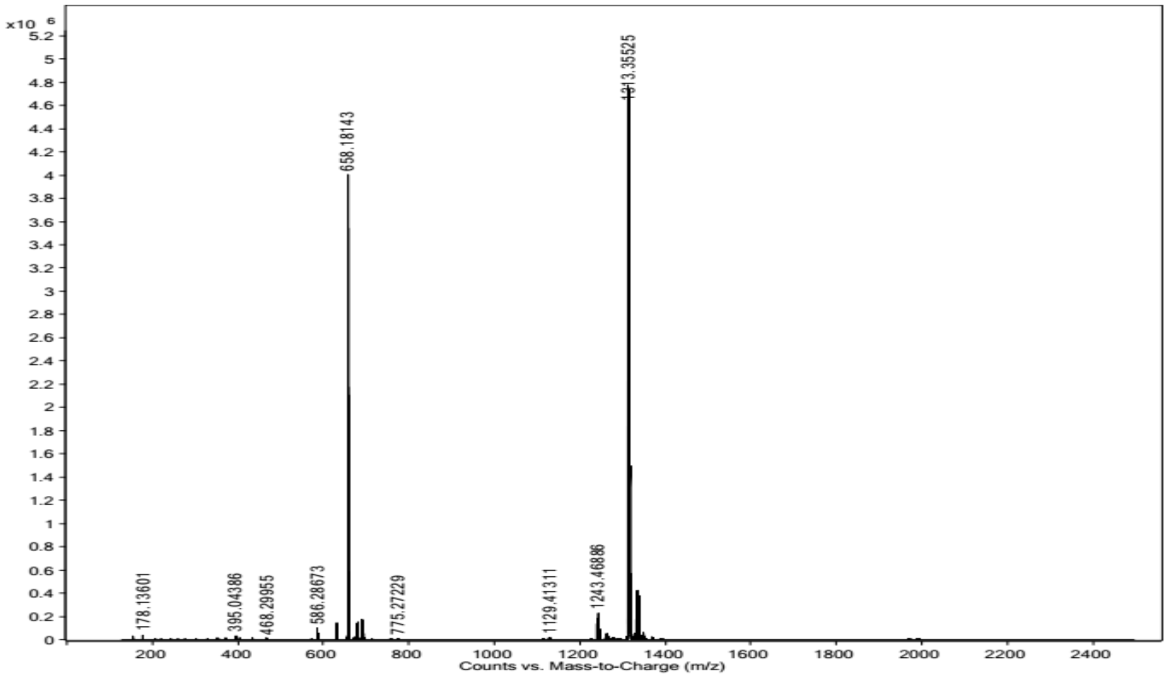
x10 6
1313.35525
658.18143
178.13601
395.04386
468.29955
586.28673
775.27229
1129.41311
1243.46886
Counts vs. Mass-to-Charge (m/z)

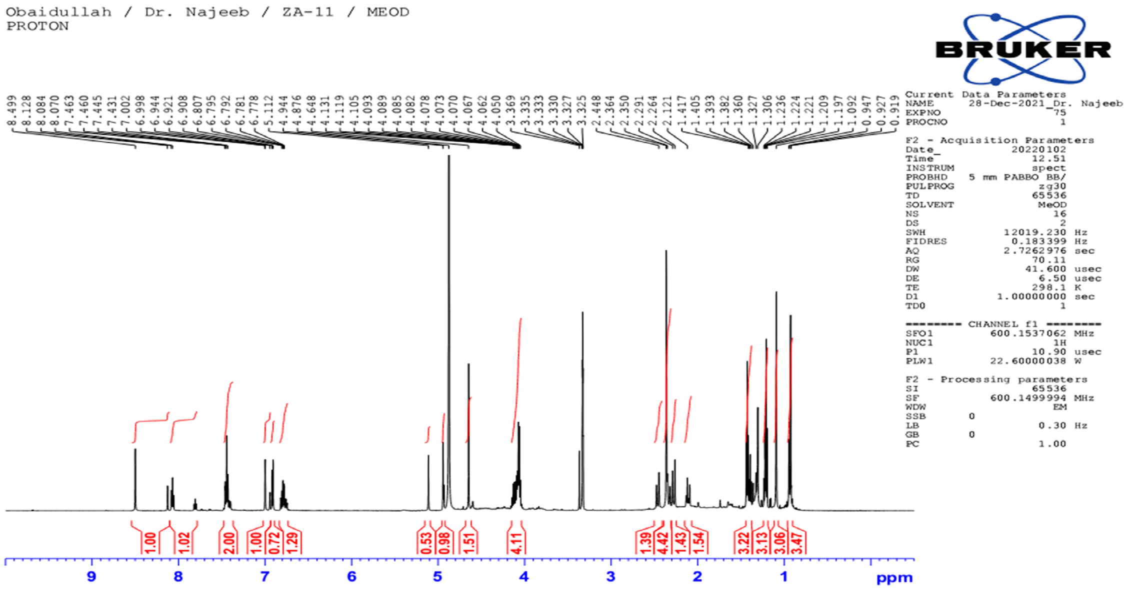
Obaidullah / Dr. Najeeb / ZA-11 / MEOD
PROTON
BRUKER
8.499 8.128 8.084 8.070 7.463 7.460 7.445 7.431 7.002 6.998 6.944 6.921 6.908 6.807 6.795 6.792 6.781 6.778 5.112 4.944 4.876 4.648 4.131 4.119 4.105 4.093 4.089 4.085 4.082 4.078 4.073 4.070 4.067 4.062 4.050 3.369 3.335 3.333 3.330 3.327 3.325 2.448 2.364 2.350 2.291 2.264 2.121 1.417 1.405 1.393 1.382 1.360 1.327 1.306 1.236 1.224 1.221 1.209 1.197 1.092 0.947 0.927 0.919
1.00 1.02 2.00 1.00 0.72 1.29 0.53 0.98 1.51 4.11 1.39 4.42 1.43 1.54 3.22 3.13 3.06 3.47
ppm
Current Data Parameters
NAME 28-Dec-2021_Dr. Najeeb
EXPNO 75
PROCNO 1
F2 - Acquisition Parameters
Date_ 20220102
Time_ 12.51
INSTRUM spect
PROBHD 5 mm PABBO BB/
PULPROG zg30
TD 65536
SOLVENT MeOD
NS 16
DS 2
SWH 12019.230 Hz
FIDRES 0.183399 Hz
AQ 2.7262976 sec
RG 70.11
DW 41.600 usec
DE 6.50 usec
TE 298.1 K
D1 1.00000000 sec
TD0 1
======== CHANNEL f1 ========
SFO1 600.1537062 MHz
NUC1 1H
P1 10.90 usec
PLW1 22.60000038 W
F2 - Processing parameters
SI 65536
SF 600.1499994 MHz
WDW EM
SSB 0
LB 0.30 Hz
GB 0
PC 1.00

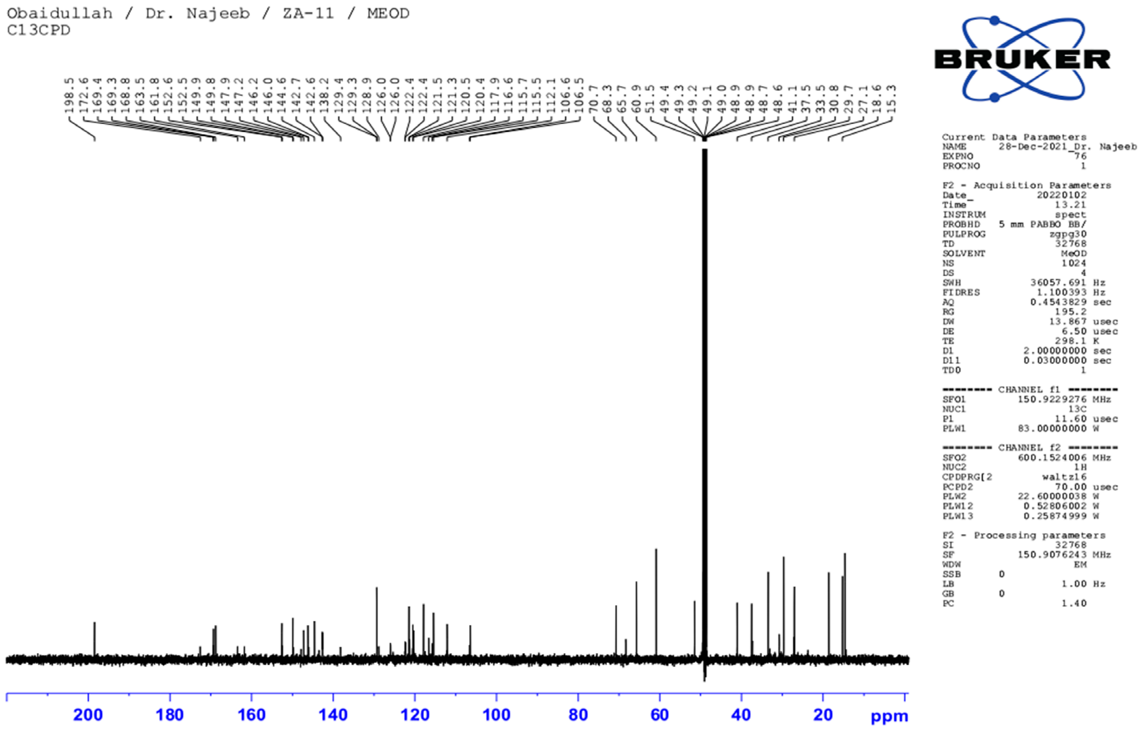

Figure S15: Mass, ^{1}H- and ^{13}C-NMR spectra of compound **13**

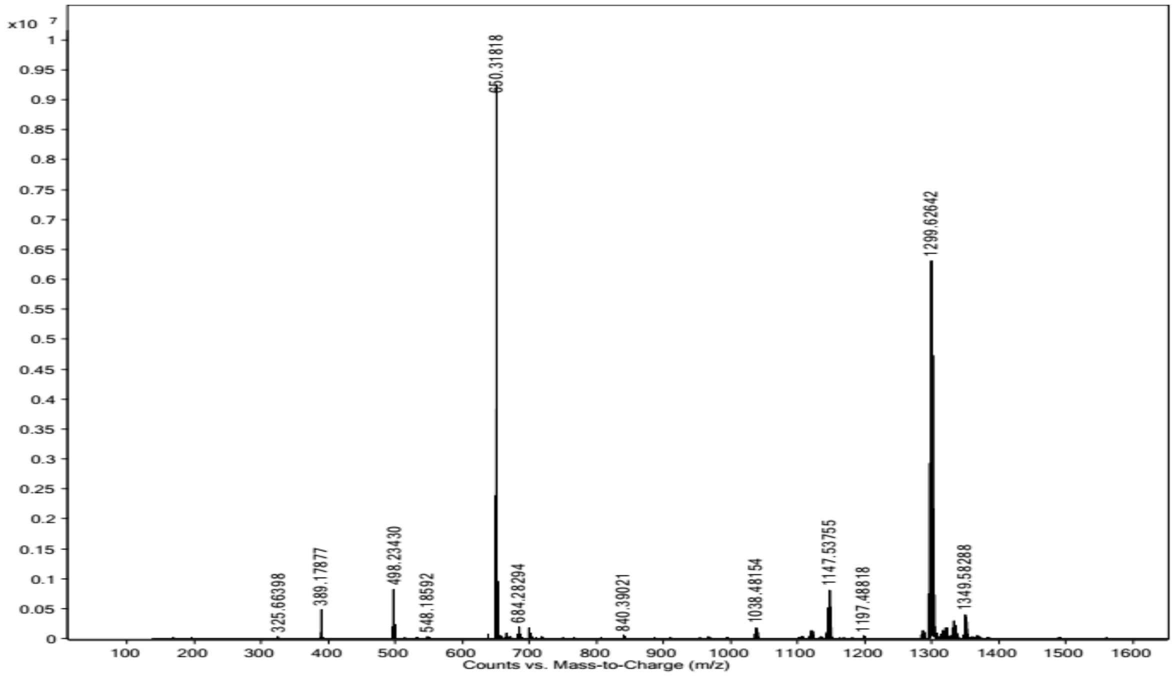

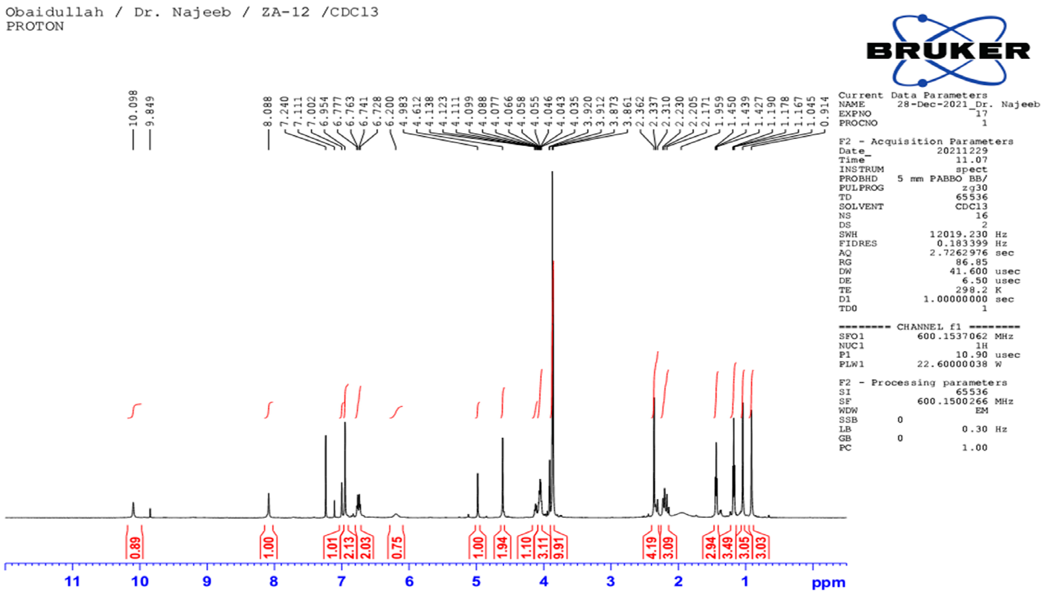

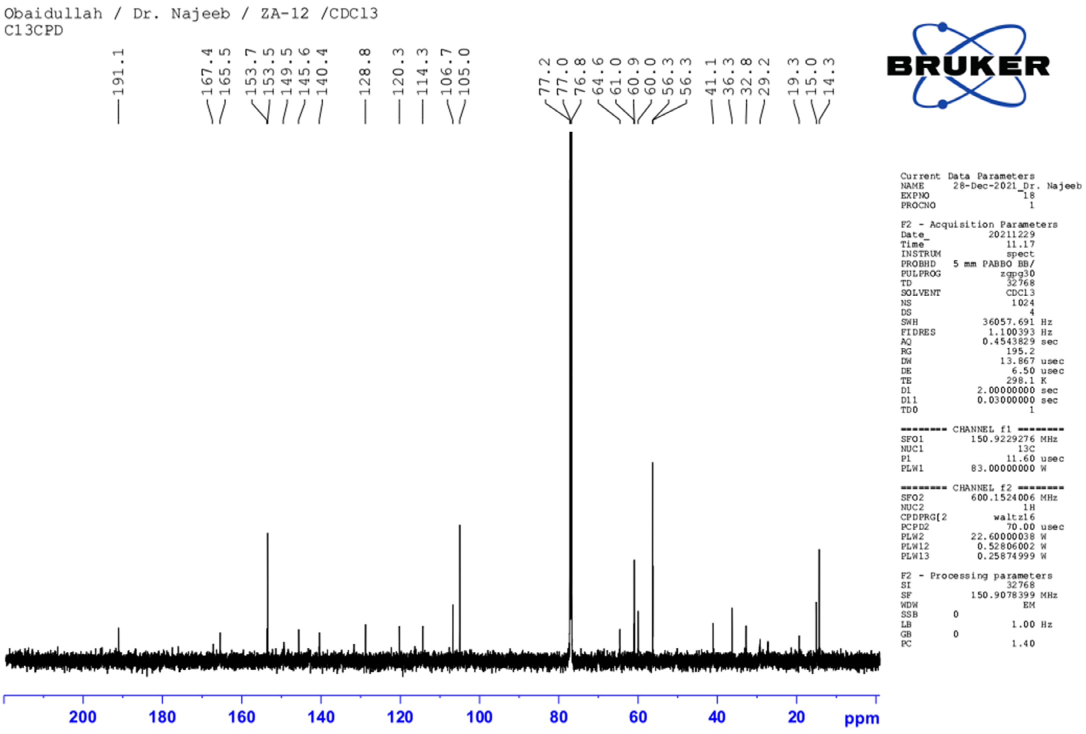

Figure S16: Mass, ^{1}H- and ^{13}C-NMR spectra of compound **14**

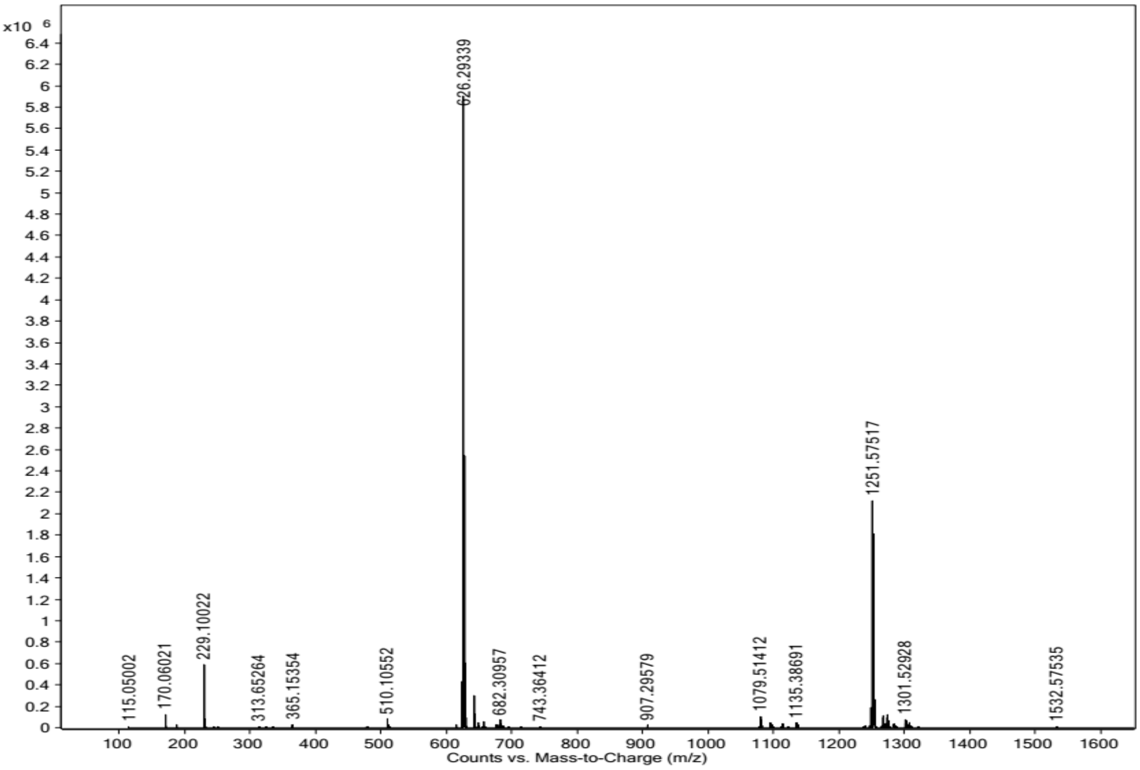
x10 6
626.29339
1251.57517
229.10022
115.05002
170.06021
313.65264
365.15354
510.10552
682.30957
743.36412
907.29579
1079.51412
1135.38691
1301.52928
1532.57535
Counts vs. Mass-to-Charge (m/z)

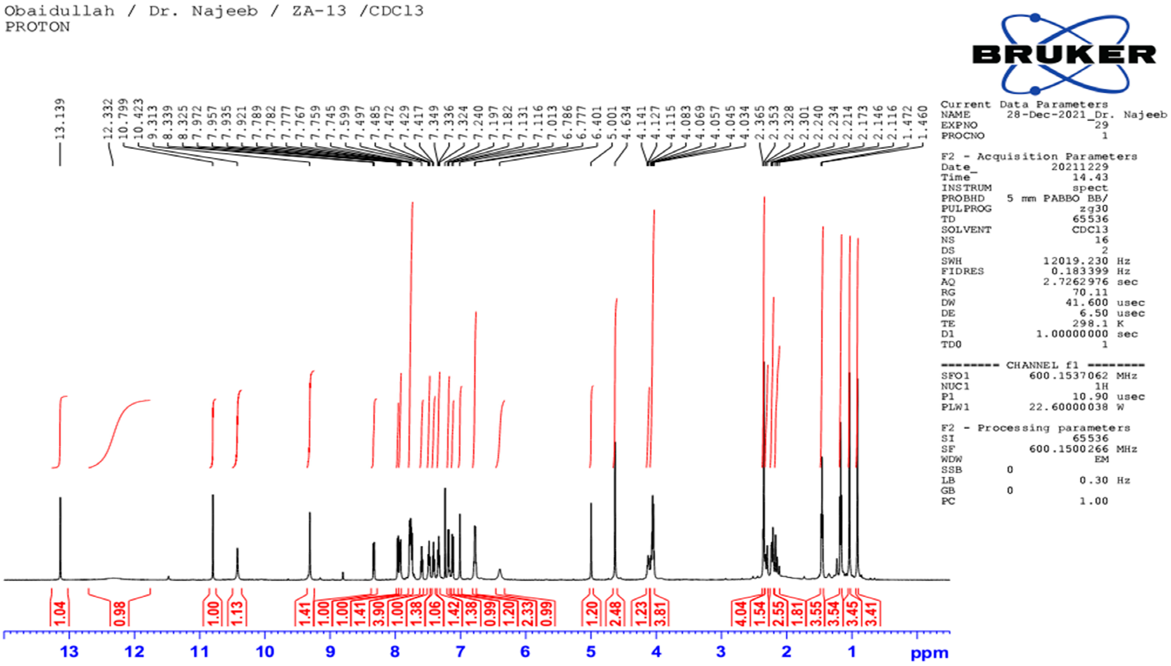
Obaidullah / Dr. Najeeb / ZA-13 /CDCl3
PROTON
13.139
12.332
10.799
10.423
9.313
8.339
8.325
7.972
7.957
7.935
7.921
7.789
7.782
7.777
7.767
7.759
7.745
7.599
7.497
7.485
7.472
7.429
7.417
7.349
7.336
7.324
7.240
7.197
7.182
7.131
7.116
7.013
6.786
6.777
6.401
5.001
4.634
4.141
4.127
4.115
4.083
4.069
4.057
4.045
4.034
2.365
2.353
2.328
2.301
2.240
2.234
2.214
2.173
2.146
2.116
1.472
1.460
BRUKER
Current Data Parameters
NAME 28-Dec-2021_Dr. Najeeb
EXPNO 29
PROCNO 1
F2 - Acquisition Parameters
Date_ 20211229
Time 14.43
INSTRUM spect
PROBHD 5 mm PABBO BB/
PULPROG zg30
TD 65536
SOLVENT CDCl3
NS 16
DS 2
SWH 12019.230 Hz
FIDRES 0.183399 Hz
AQ 2.7262976 sec
RG 70.11
DW 41.600 usec
DE 6.50 usec
TE 298.1 K
D1 1.00000000 sec
TD0 1
======== CHANNEL f1 ========
SFO1 600.1537062 MHz
NUC1 1H
P1 10.90 usec
PLW1 22.60000038 W
F2 - Processing parameters
SI 65536
SF 600.1500266 MHz
WDW EM
SSB 0
LB 0.30 Hz
GB 0
PC 1.00
1.04
0.98
1.00
1.13
1.41
1.00
1.00
1.41
3.90
1.00
1.38
1.06
1.42
1.38
0.99
1.20
2.33
0.99
1.20
2.48
1.23
3.81
4.04
1.54
2.55
1.81
3.55
3.54
3.45
3.41
ppm

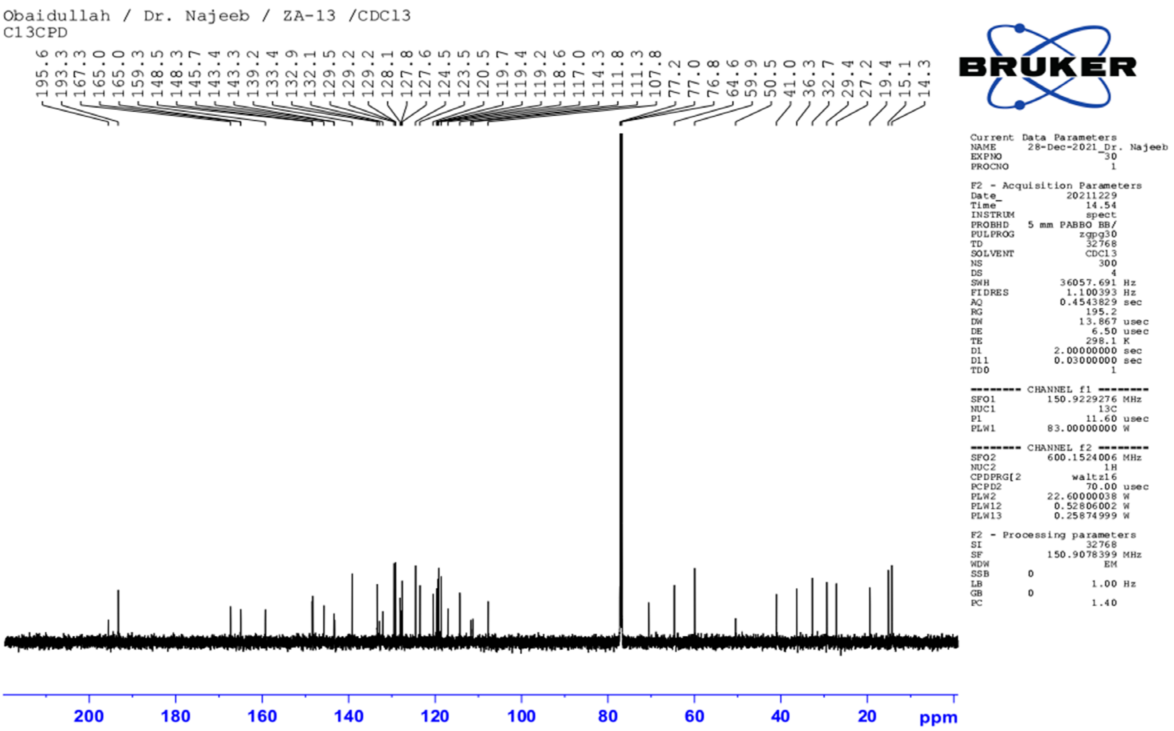

Figure S17: Mass, ^{1}H- and ^{13}C-NMR spectra of compound **15**

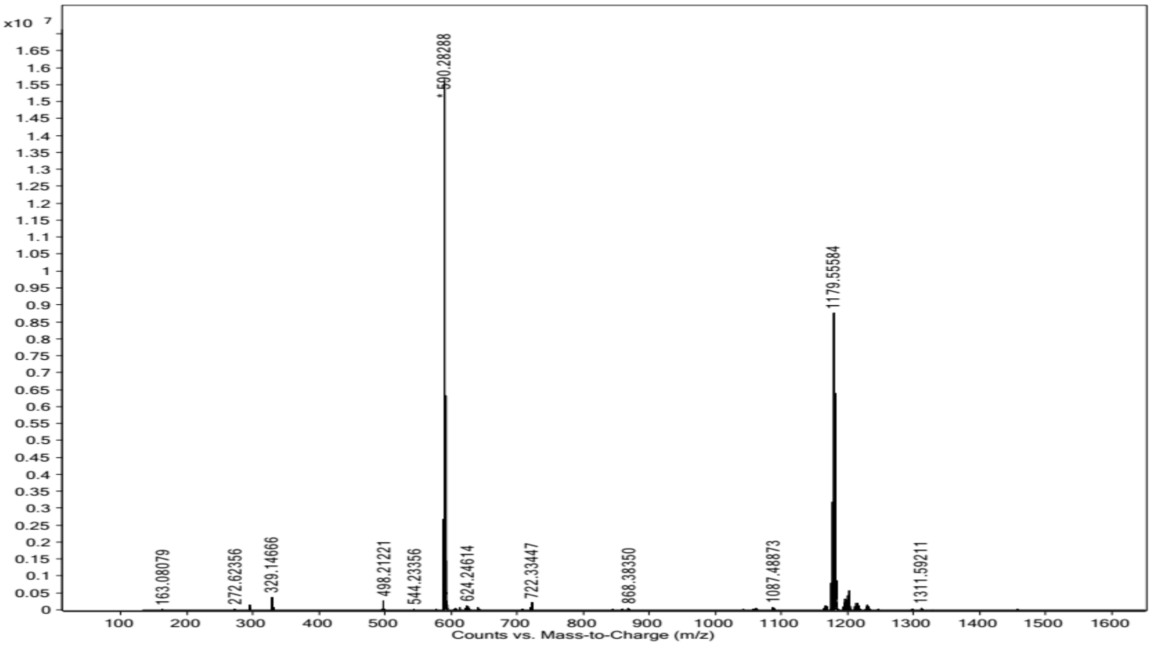

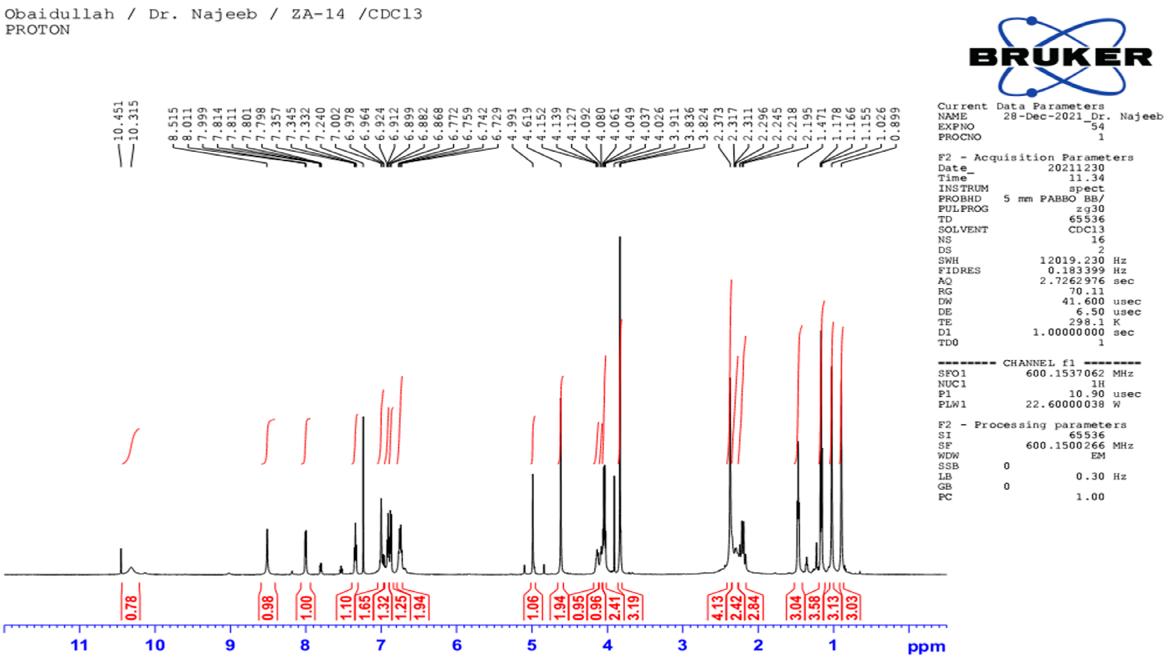

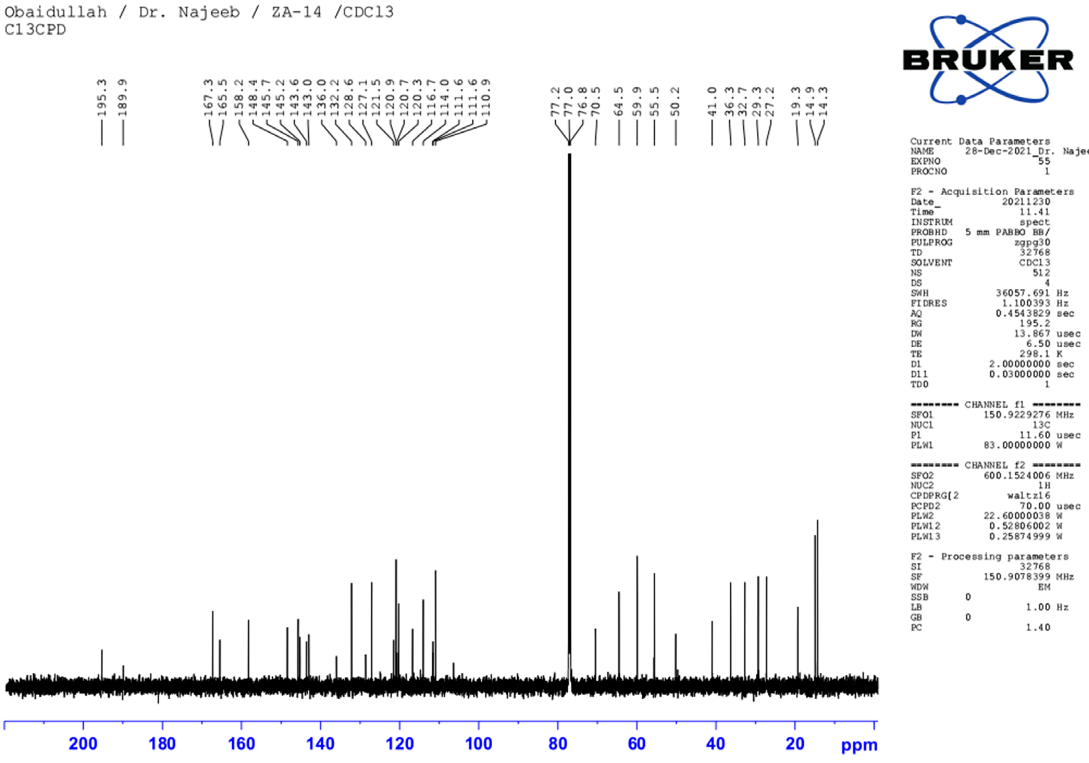

Figure S18: Mass, ^{1}H- and ^{13}C-NMR spectra of compound **16**

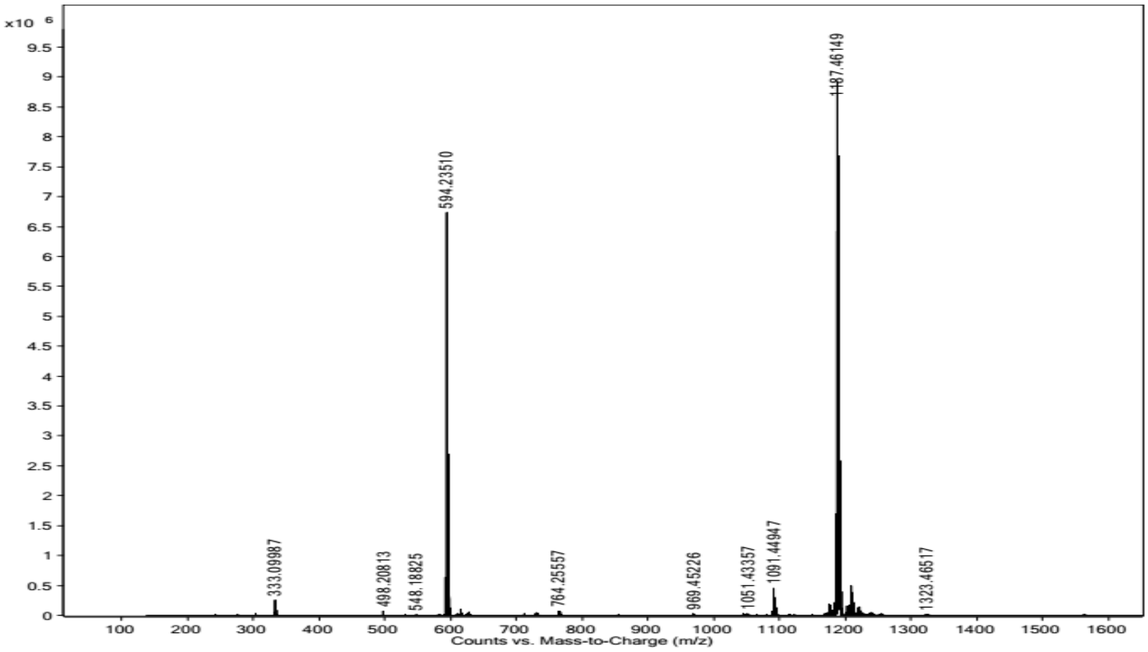
x10 6
333.09987
498.20813
548.18825
594.23510
764.25557
969.45226
1051.43357
1091.44947
1187.46149
1323.46517
Counts vs. Mass-to-Charge (m/z)

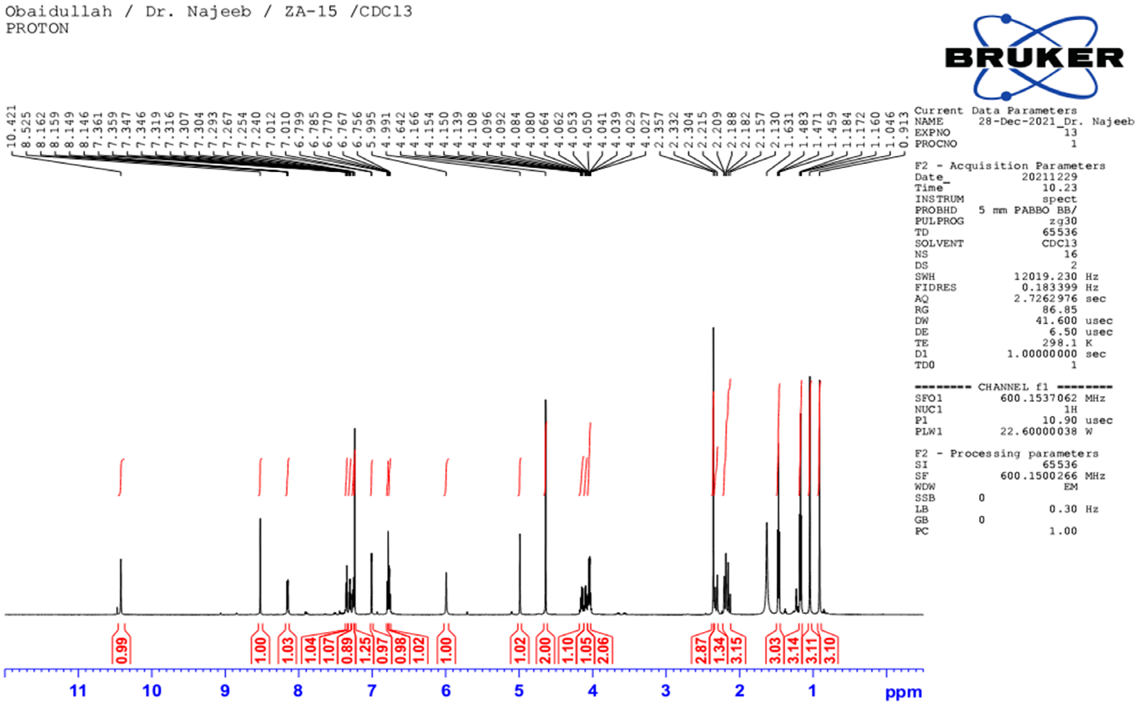
Obaidullah / Dr. Najeeb / ZA-15 /CDCl3
PROTON
BRUKER
ppm

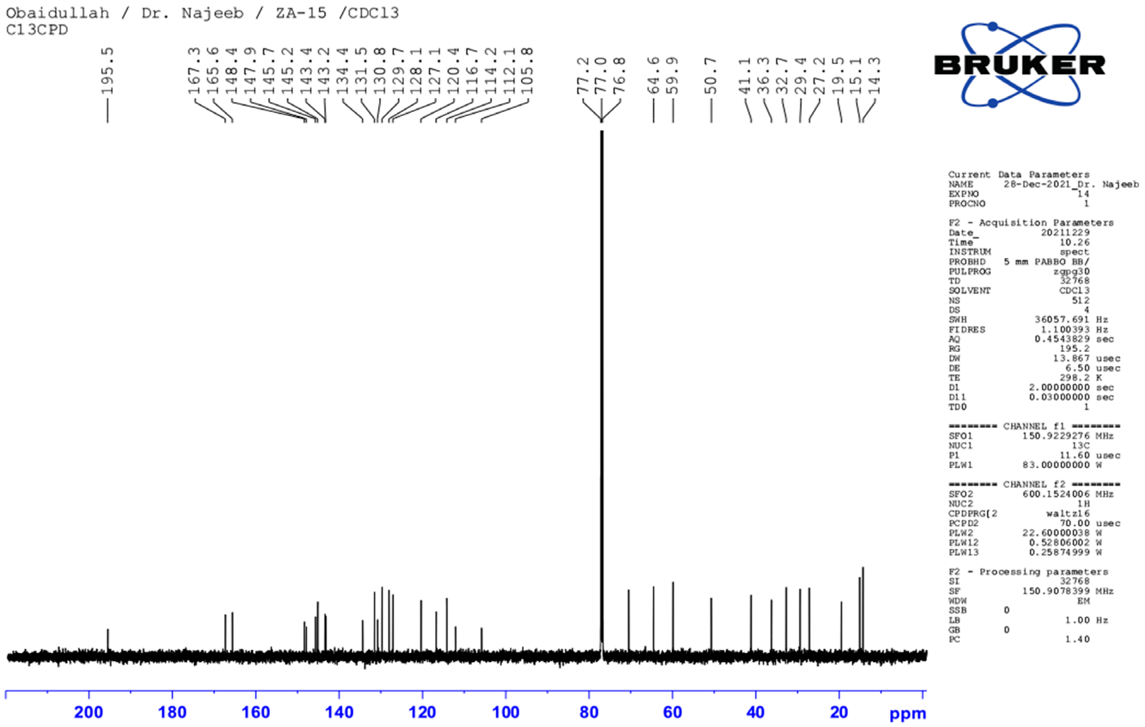

Figure S19: Mass, ^{1}H- and ^{13}C-NMR spectra of compound **17**

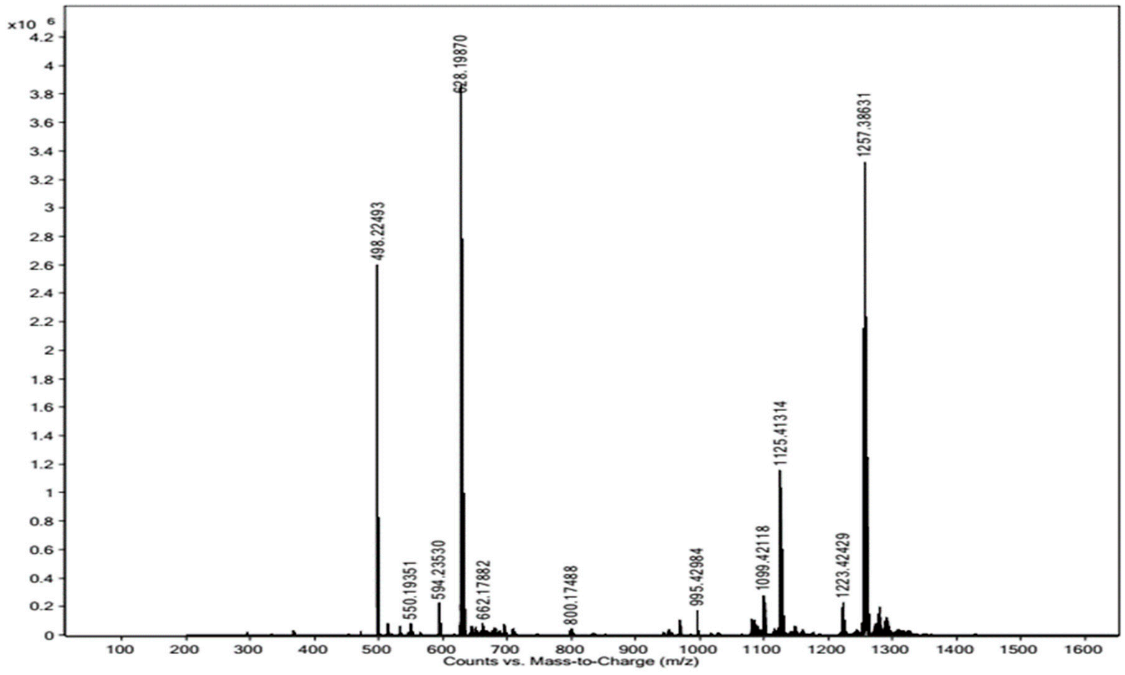

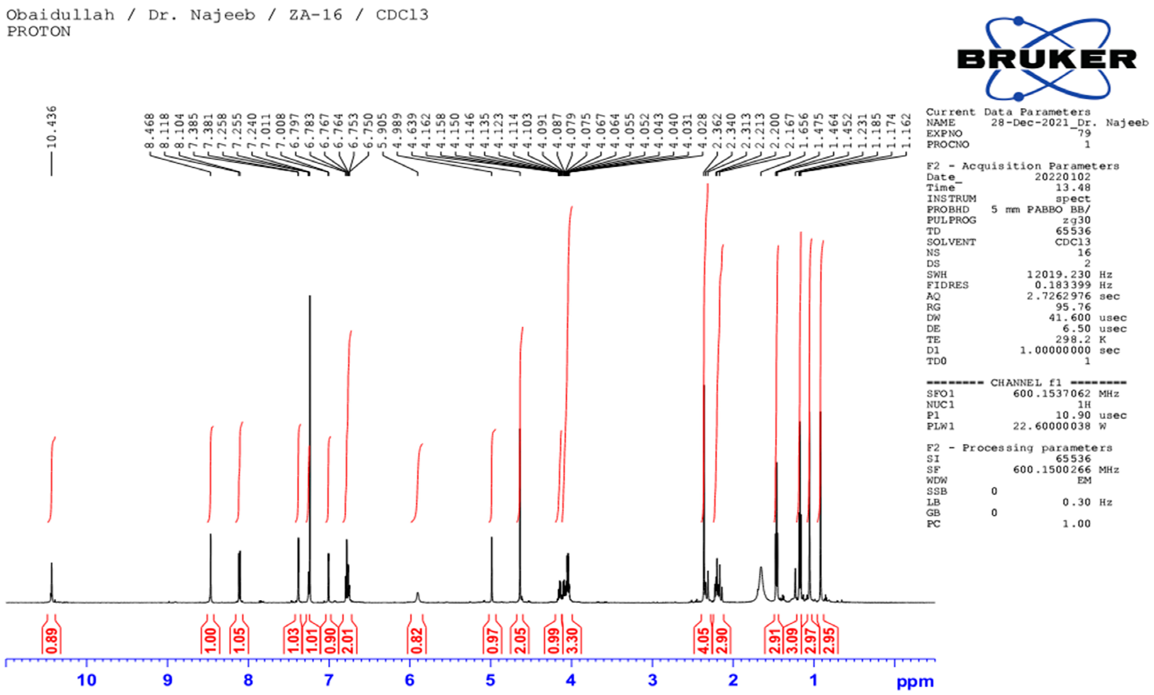

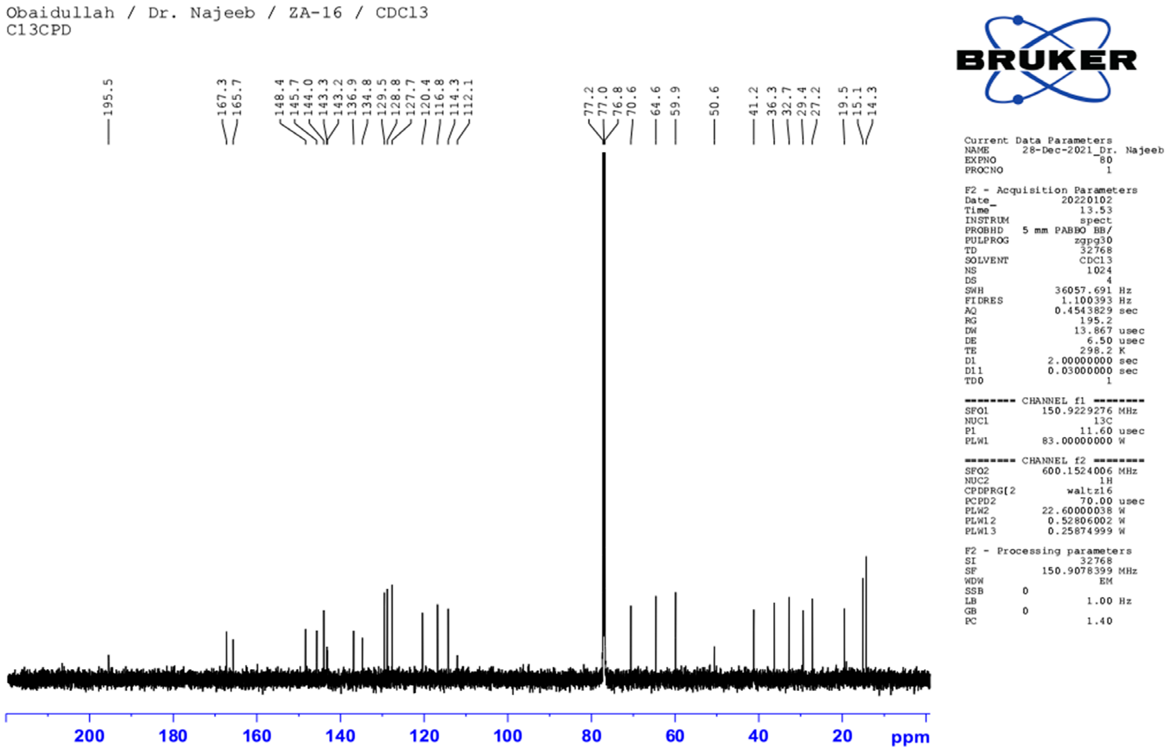

Figure S20: Mass, ^{1}H- and ^{13}C-NMR spectra of compound **18**

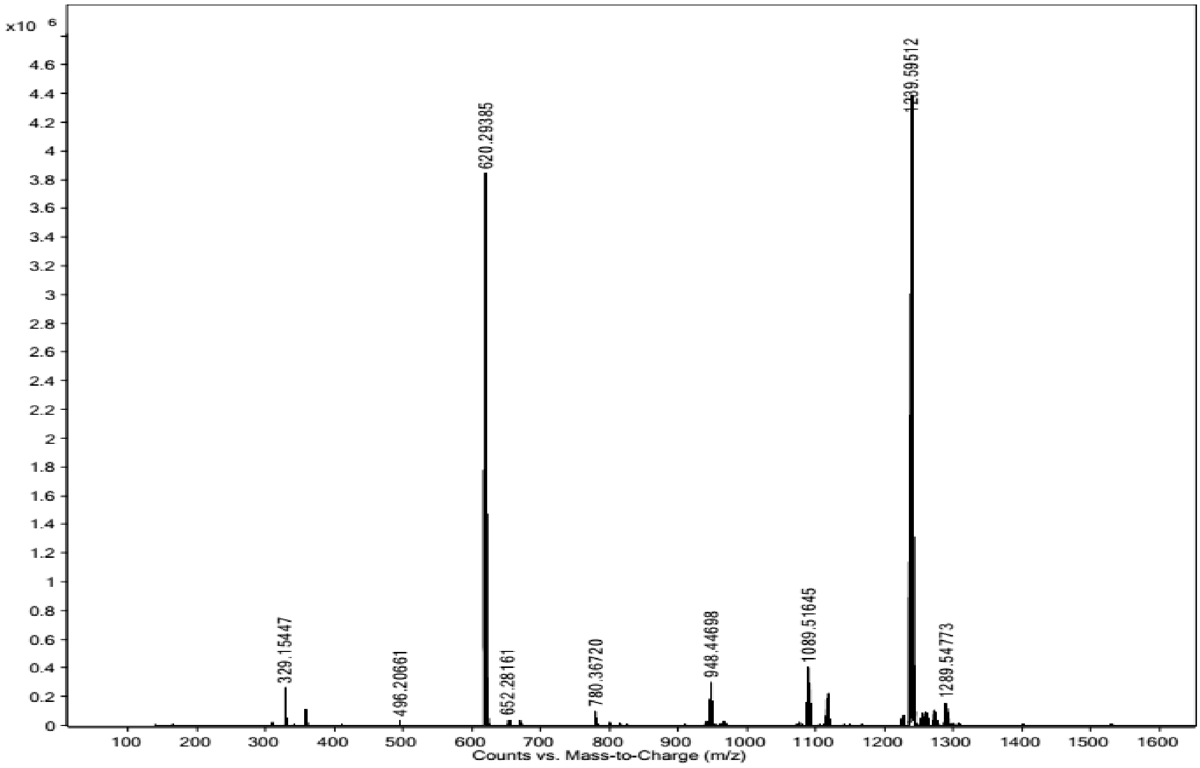
x10 6
620.29385
1239.59512
329.15447
496.20661
652.28161
780.36720
948.44698
1089.51645
1289.54773
Counts vs. Mass-to-Charge (m/z)

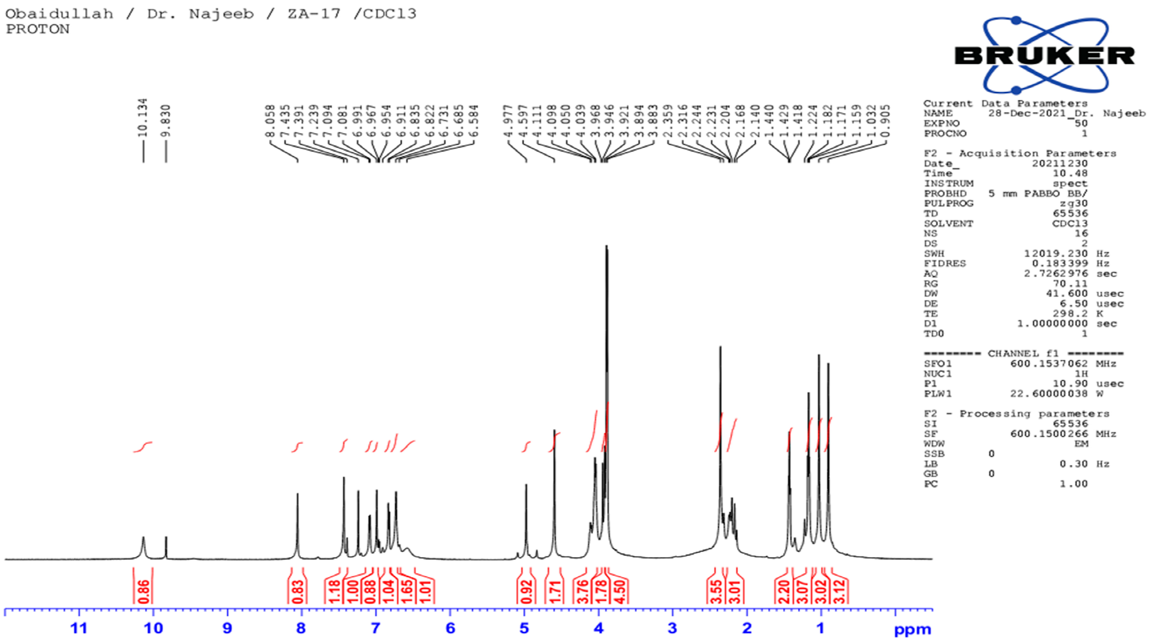
Obaidullah / Dr. Najeeb / ZA-17 /CDCl3
PROTON
BRUKER
10.134
9.830
8.058
7.435
7.391
7.239
7.094
7.081
6.991
6.967
6.954
6.911
6.835
6.822
6.731
6.685
6.584
4.977
4.597
4.111
4.098
4.050
4.039
3.968
3.946
3.921
3.894
3.883
2.359
2.316
2.244
2.231
2.204
2.168
2.140
1.440
1.429
1.418
1.224
1.182
1.171
1.159
1.032
0.905
Current Data Parameters
NAME 28-Dec-2021_Dr. Najeeb
EXPNO 50
PROCNO 1
F2 - Acquisition Parameters
Date_ 20211230
Time 10.48
INSTRUM spect
PROBHD 5 mm PABBO BB/
PULPROG zg30
TD 65536
SOLVENT CDCl3
NS 16
DS 2
SWH 12019.230 Hz
FIDRES 0.183399 Hz
AQ 2.7262976 sec
RG 70.11
DW 41.600 usec
DE 6.50 usec
TE 298.2 K
D1 1.00000000 sec
TD0 1
======== CHANNEL f1 ========
SFO1 600.1537062 MHz
NUC1 1H
P1 10.90 usec
PLW1 22.60000038 W
F2 - Processing parameters
SI 65536
SF 600.1500266 MHz
WDW EM
SSB 0
LB 0.30 Hz
GB 0
PC 1.00
0.86
0.83
1.18
1.00
0.88
1.04
1.65
1.01
0.92
1.71
3.76
1.78
4.50
3.55
3.01
2.20
3.07
3.02
3.12
ppm

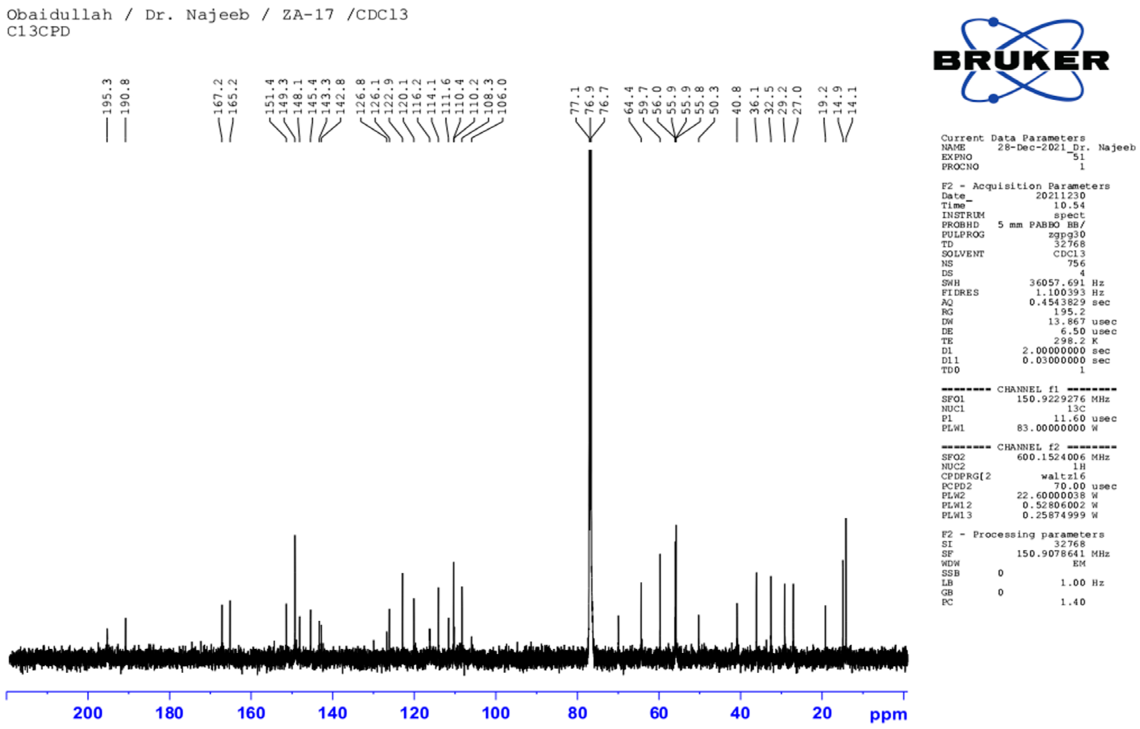

Figure S21: Mass, ^{1}H- and ^{13}C-NMR spectra of compound **19**

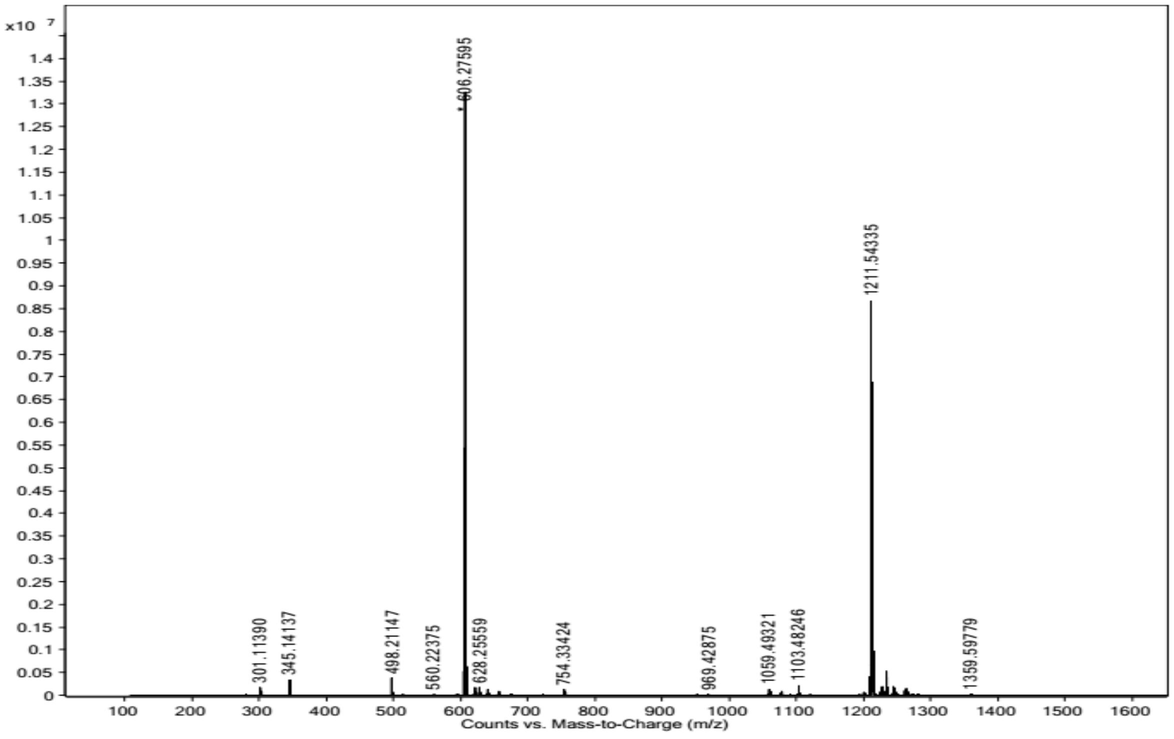

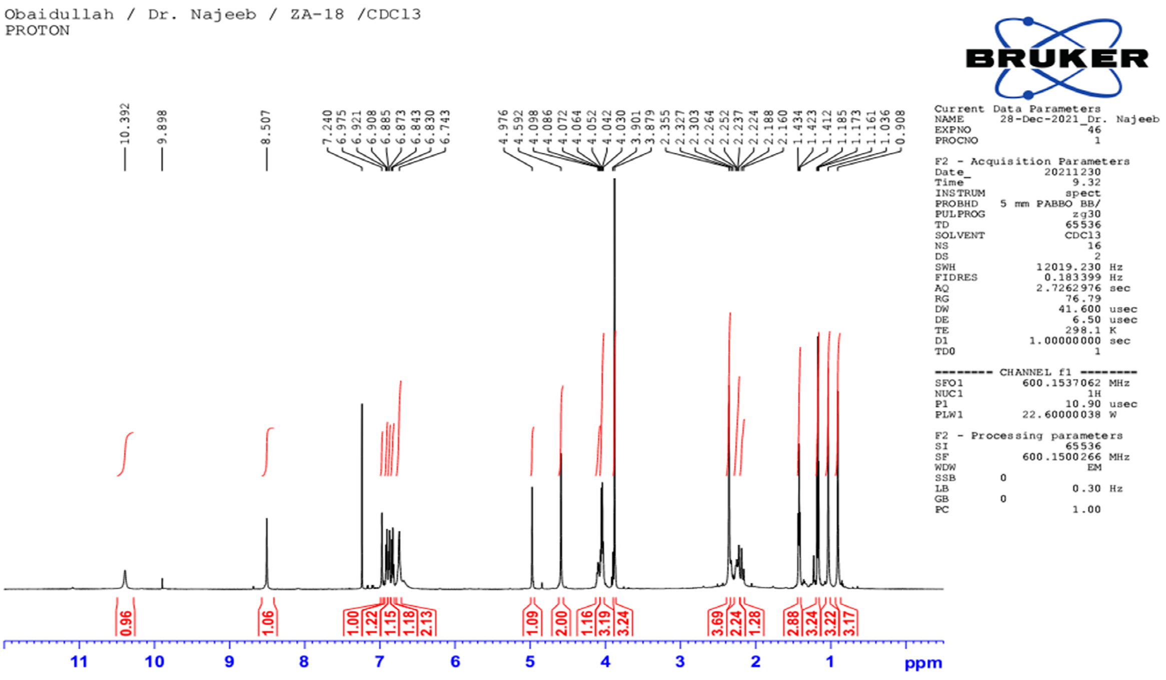

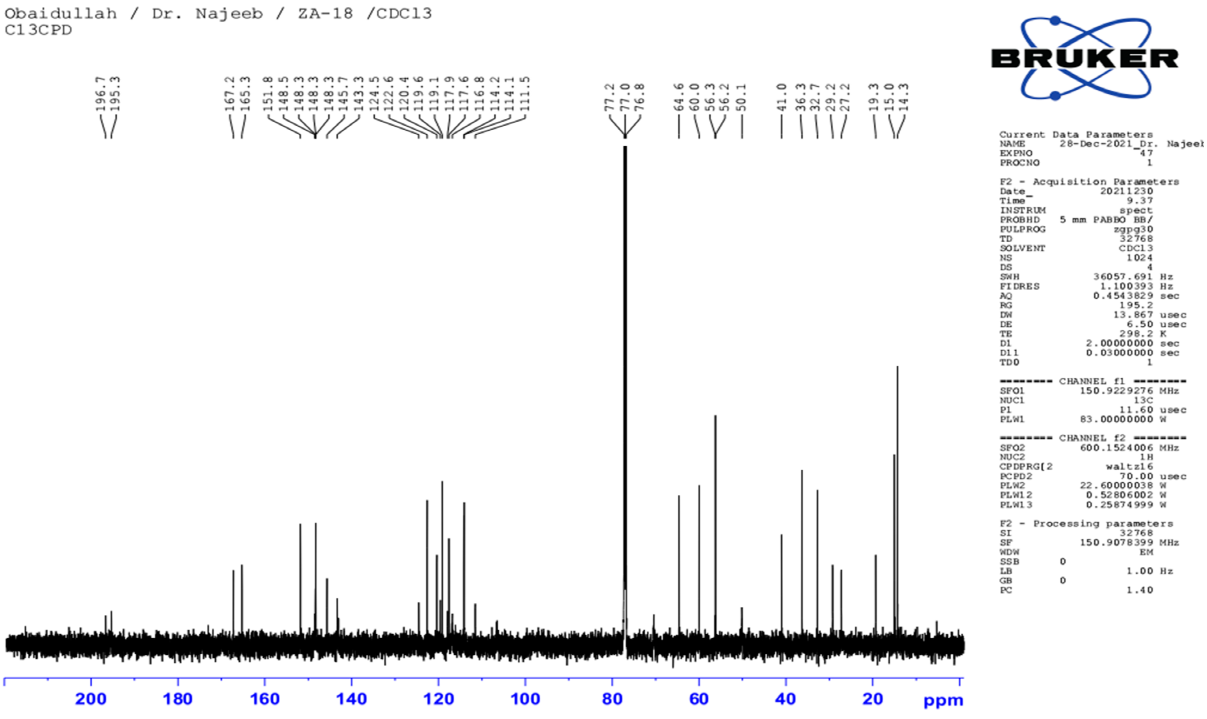

Figure S22: Mass, ^{1}H-NMR and ^{13}C-NMR spectra of compound **20**

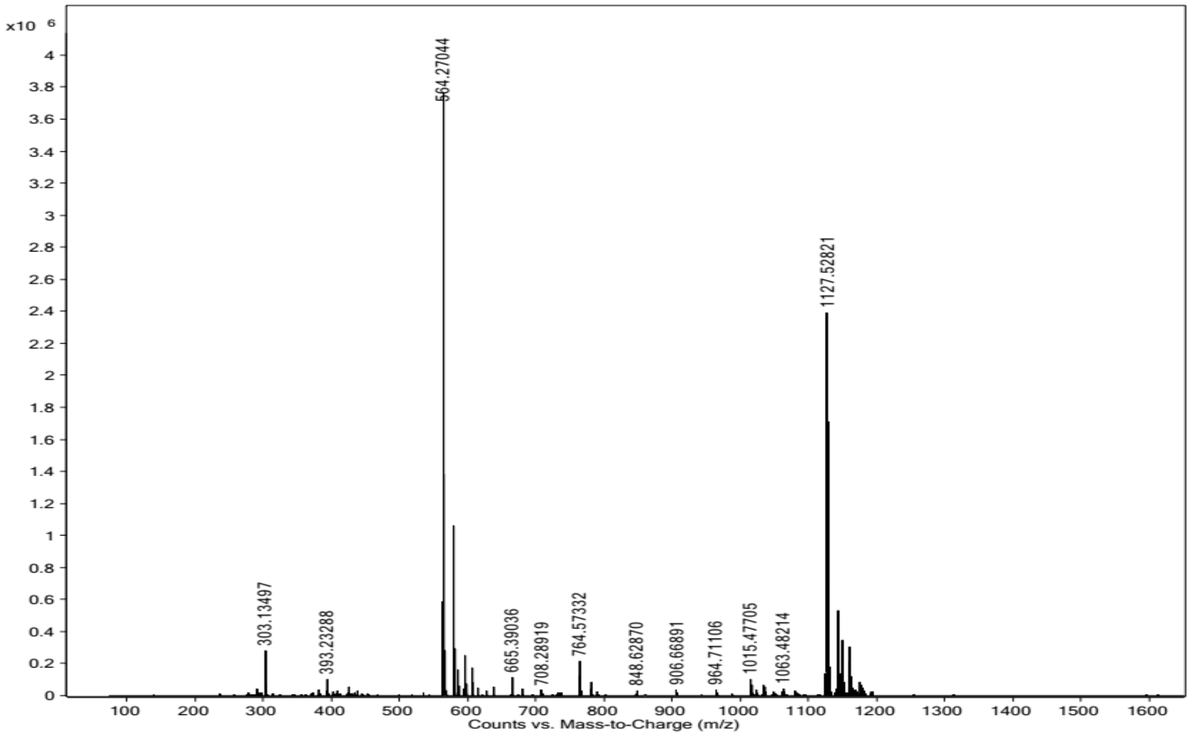

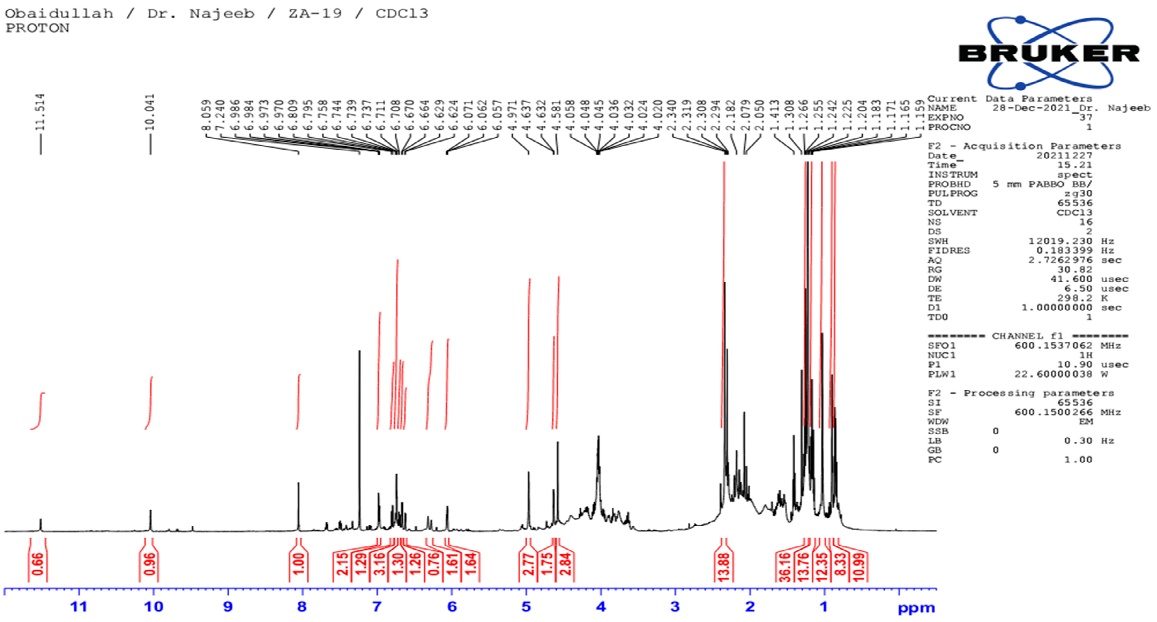

Figure S23: Mass and ^{1}H-NMR spectra of compound **21**

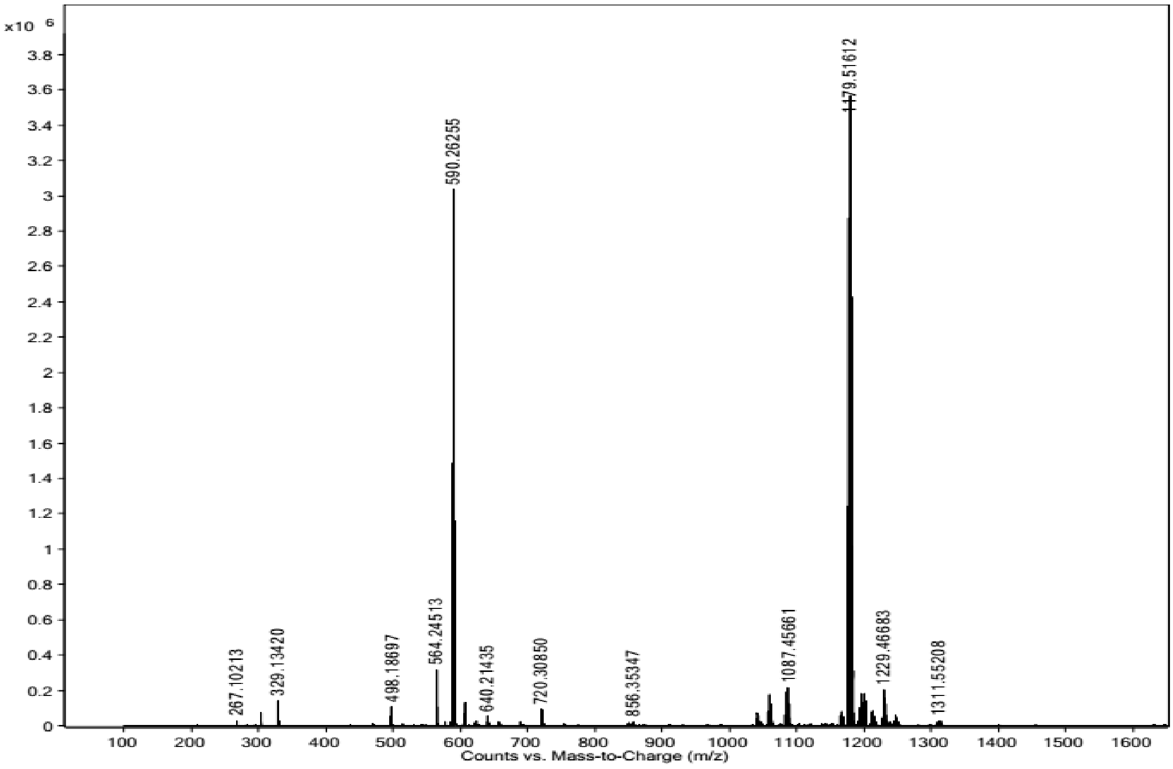
x10 6
267.10213
329.13420
498.18697
564.24513
590.26255
640.21435
720.30850
856.35347
1087.45661
1179.51612
1229.46683
1311.55208
Counts vs. Mass-to-Charge (m/z)

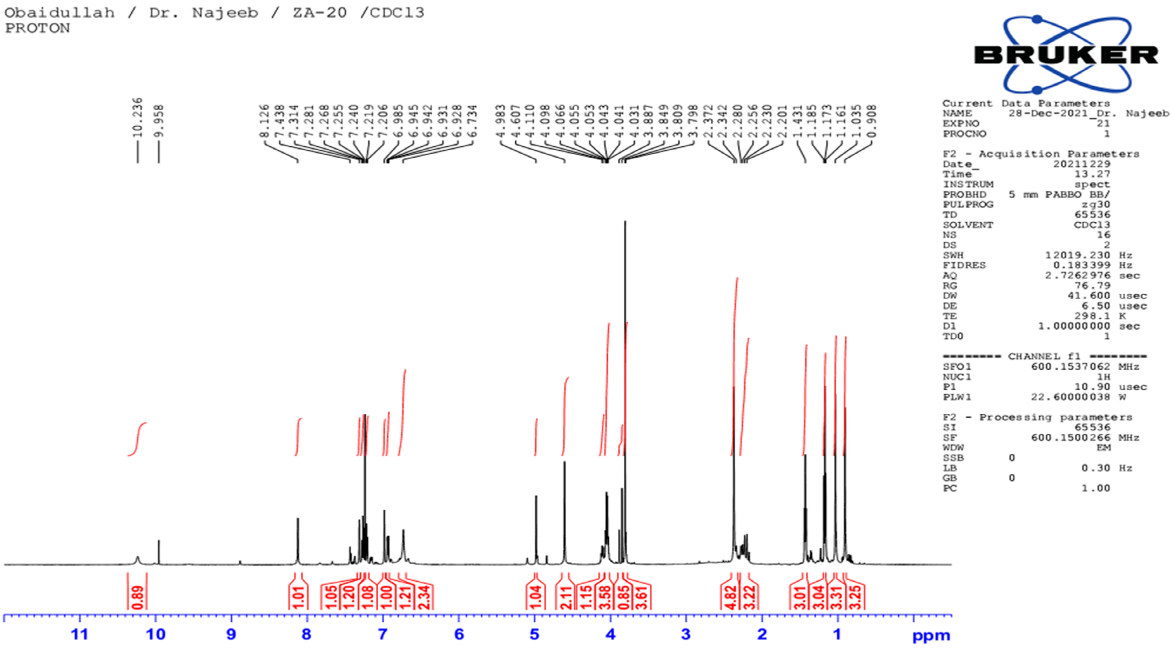
Obaidullah / Dr. Najeeb / ZA-20 /CDCl3
PROTON
10.236
9.958
8.126
7.438
7.314
7.281
7.268
7.255
7.240
7.219
7.206
6.985
6.945
6.942
6.931
6.928
6.734
4.983
4.607
4.110
4.098
4.066
4.055
4.053
4.043
4.041
4.031
3.887
3.849
3.809
3.798
2.372
2.342
2.280
2.256
2.230
2.201
1.431
1.185
1.173
1.161
1.035
0.908
BRUKER
Current Data Parameters
NAME 28-Dec-2021_Dr. Najeeb
EXPNO 21
PROCNO 1
F2 - Acquisition Parameters
Date_ 20211229
Time 13.27
INSTRUM spect
PROBHD 5 mm PABBO BB/
PULPROG zg30
TD 65536
SOLVENT CDCl3
NS 16
DS 2
SWH 12019.230 Hz
FIDRES 0.183399 Hz
AQ 2.7262976 sec
RG 76.79
DW 41.600 usec
DE 6.50 usec
TE 298.1 K
D1 1.00000000 sec
TD0 1
======== CHANNEL f1 ========
SFO1 600.1537062 MHz
NUC1 1H
P1 10.90 usec
PLW1 22.60000038 W
F2 - Processing parameters
SI 65536
SF 600.1500266 MHz
WDW EM
SSB 0
LB 0.30 Hz
GB 0
PC 1.00
0.89
1.01
1.05
1.20
1.08
1.00
1.21
2.34
1.04
2.11
1.15
3.58
0.85
3.61
4.82
3.22
3.01
3.04
3.31
3.25
ppm

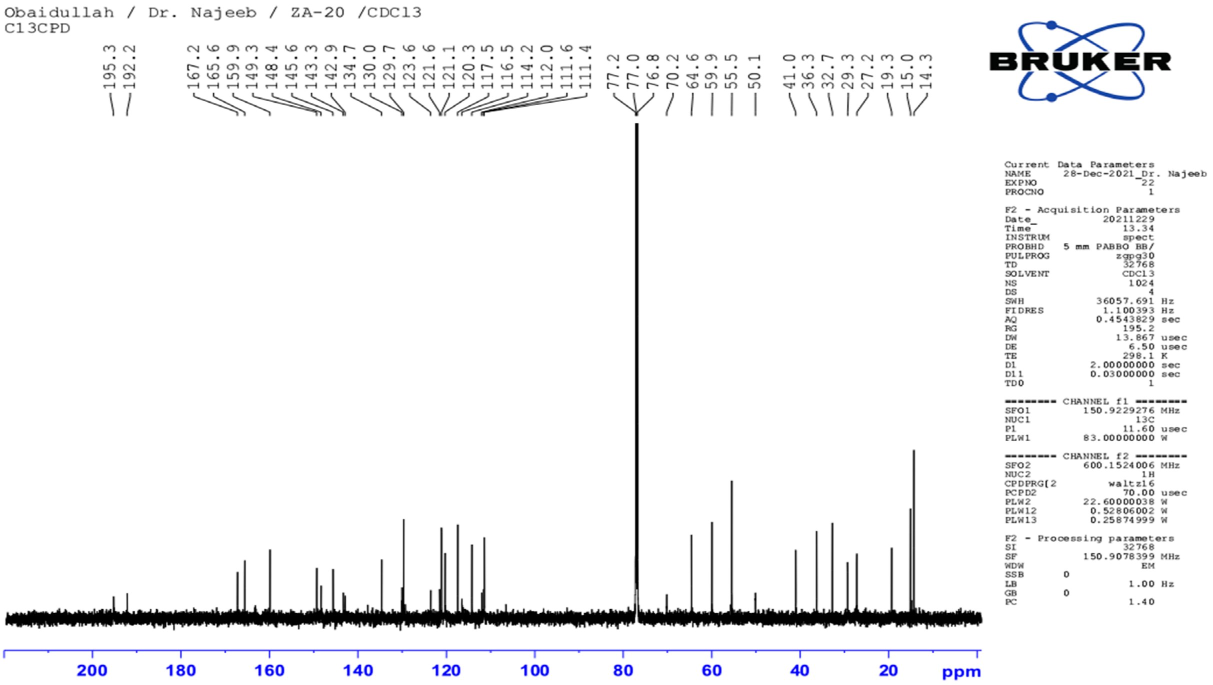

Figure S24: Mass, ^{1}H- and ^{13}C-NMR spectra of compound **22**

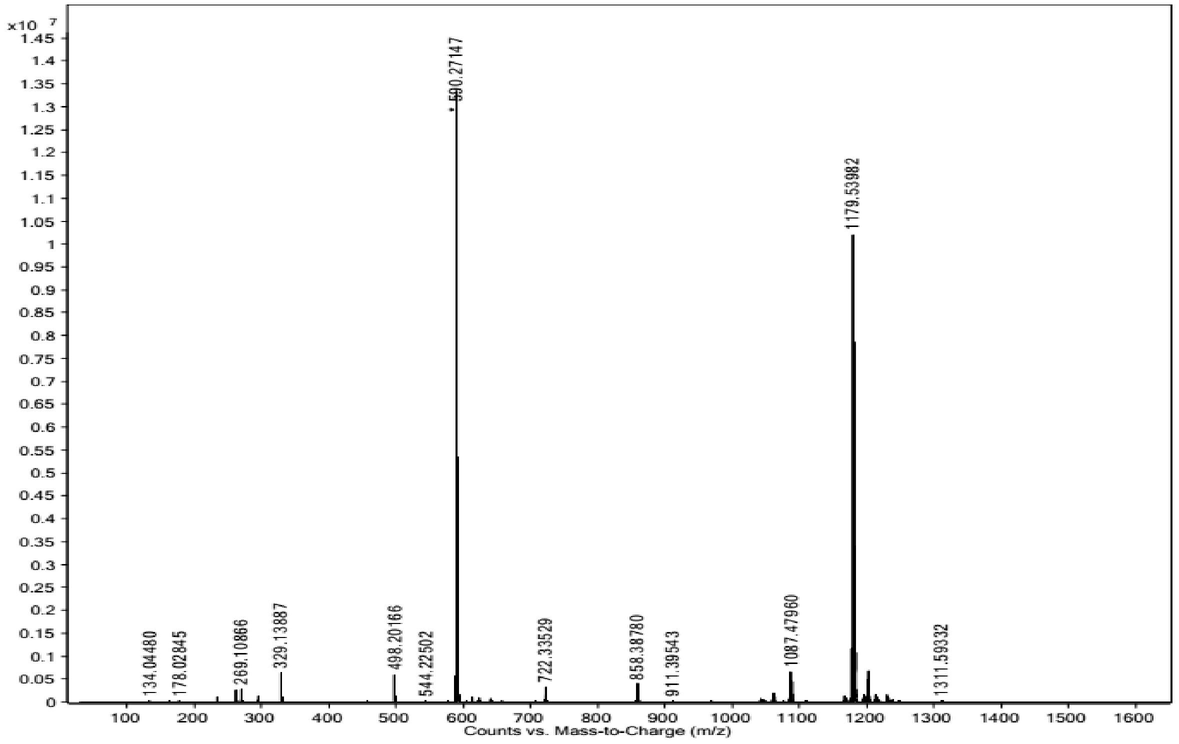

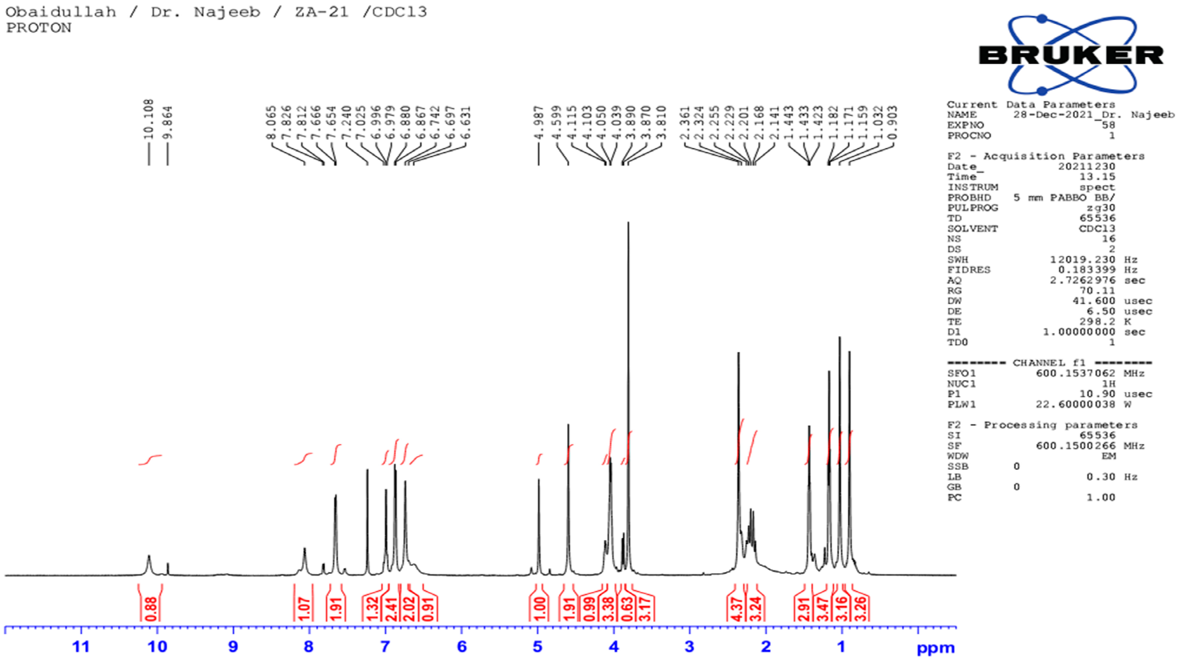

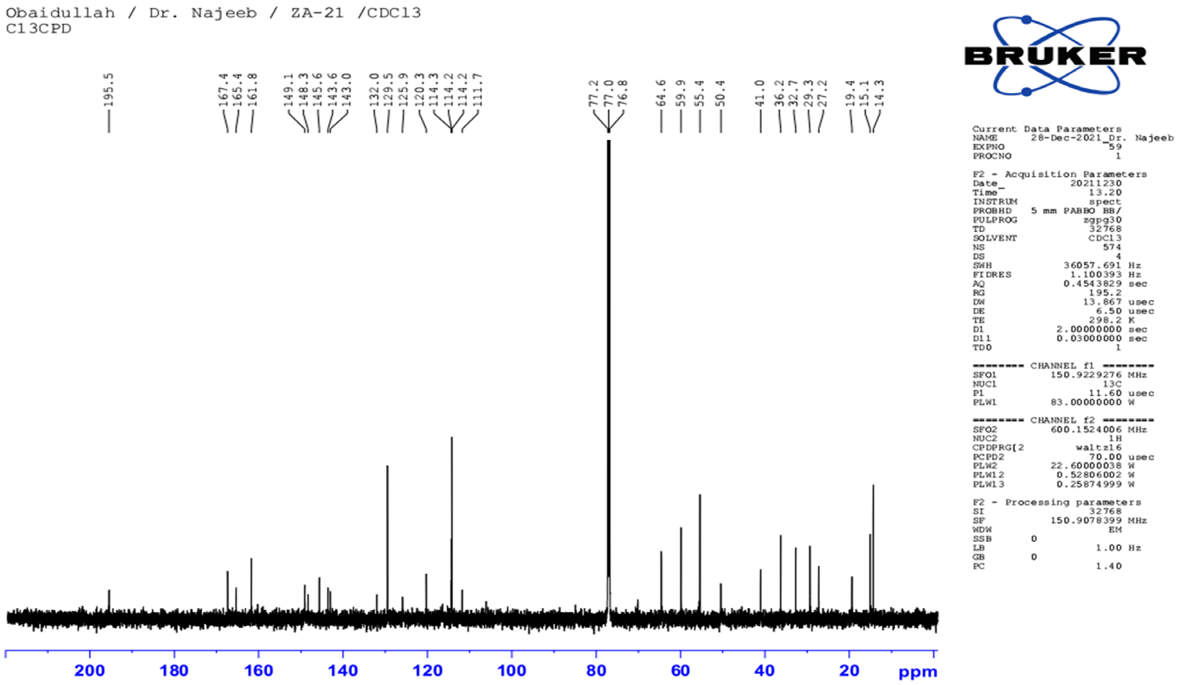

Figure S25: Mass, ^{1}H- and ^{13}C-NMR spectra of compound **23**

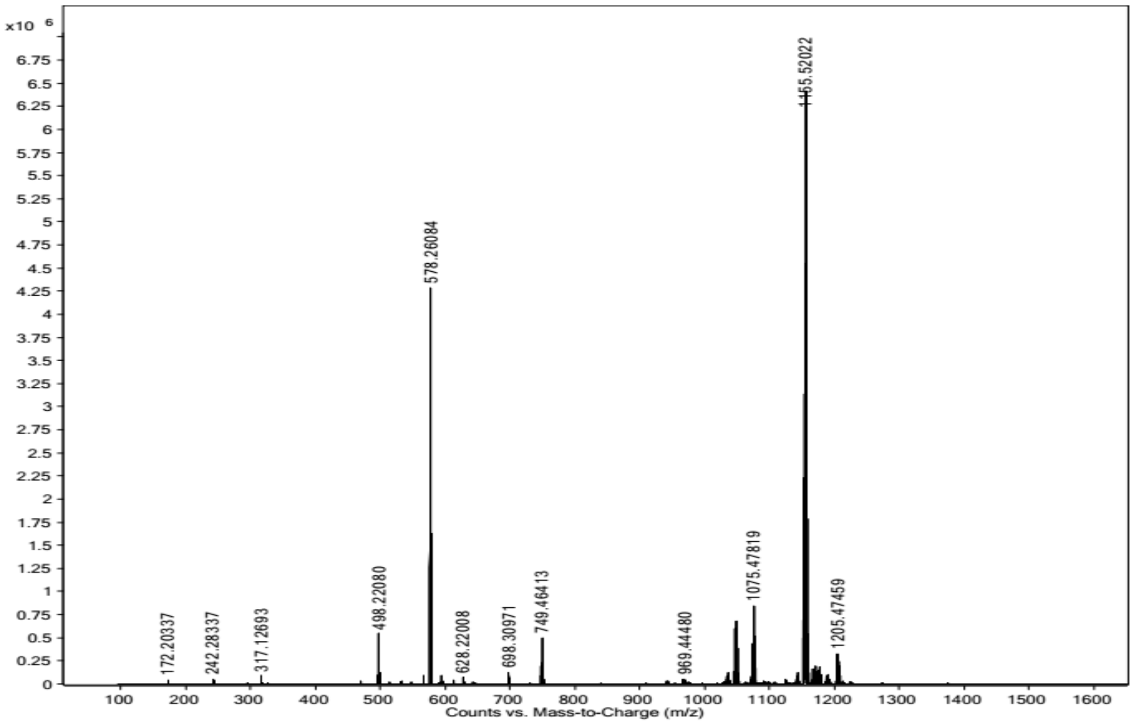
x10 6
1155.52022
578.26084
498.22080
749.46413
1075.47819
1205.47459
172.20337
242.28337
317.12693
628.22008
698.30971
969.44480
Counts vs. Mass-to-Charge (m/z)

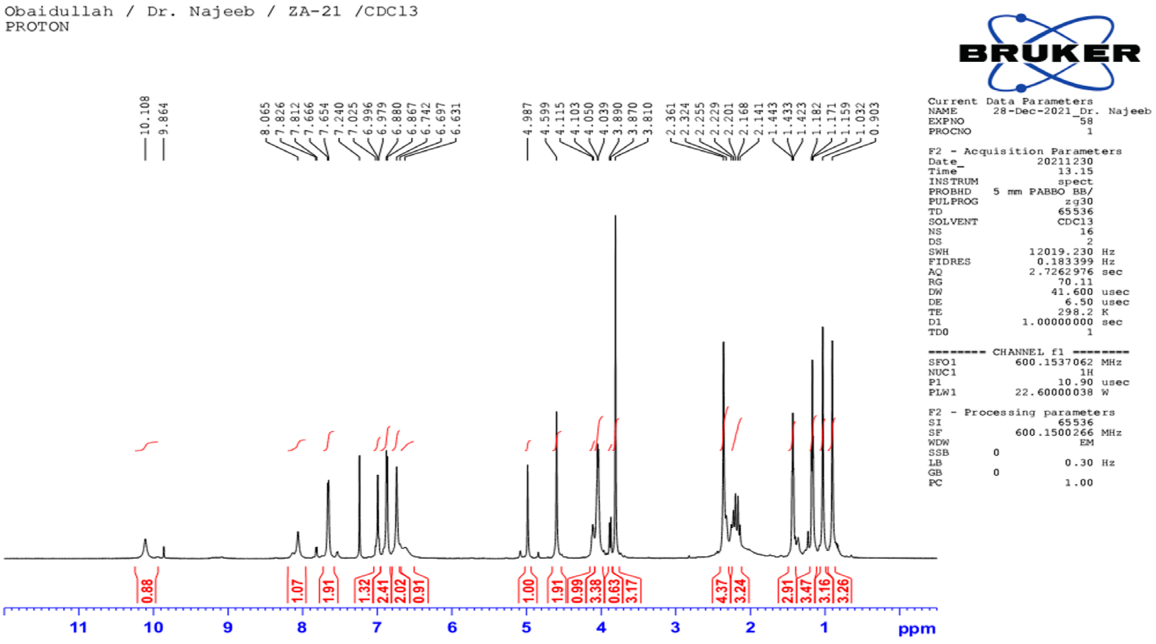
Obaidullah / Dr. Najeeb / ZA-21 /CDCl3
PROTON
BRUKER
Current Data Parameters
NAME 28-Dec-2021_Dr. Najeeb
EXPNO 58
PROCNO 1
F2 - Acquisition Parameters
Date_ 20211230
Time 13.15
INSTRUM spect
PROBHD 5 mm PABBO BB/
PULPROG zg30
TD 65536
SOLVENT CDCl3
NS 16
DS 2
SWH 12019.230 Hz
FIDRES 0.183399 Hz
AQ 2.7262976 sec
RG 70.11
DW 41.600 usec
DE 6.50 usec
TE 298.2 K
D1 1.00000000 sec
TD0 1
======== CHANNEL f1 ========
SFO1 600.1537062 MHz
NUC1 1H
P1 10.90 usec
PLW1 22.60000038 W
F2 - Processing parameters
SI 65536
SF 600.1500266 MHz
WDW EM
SSB 0
LB 0.30 Hz
GB 0
PC 1.00
ppm

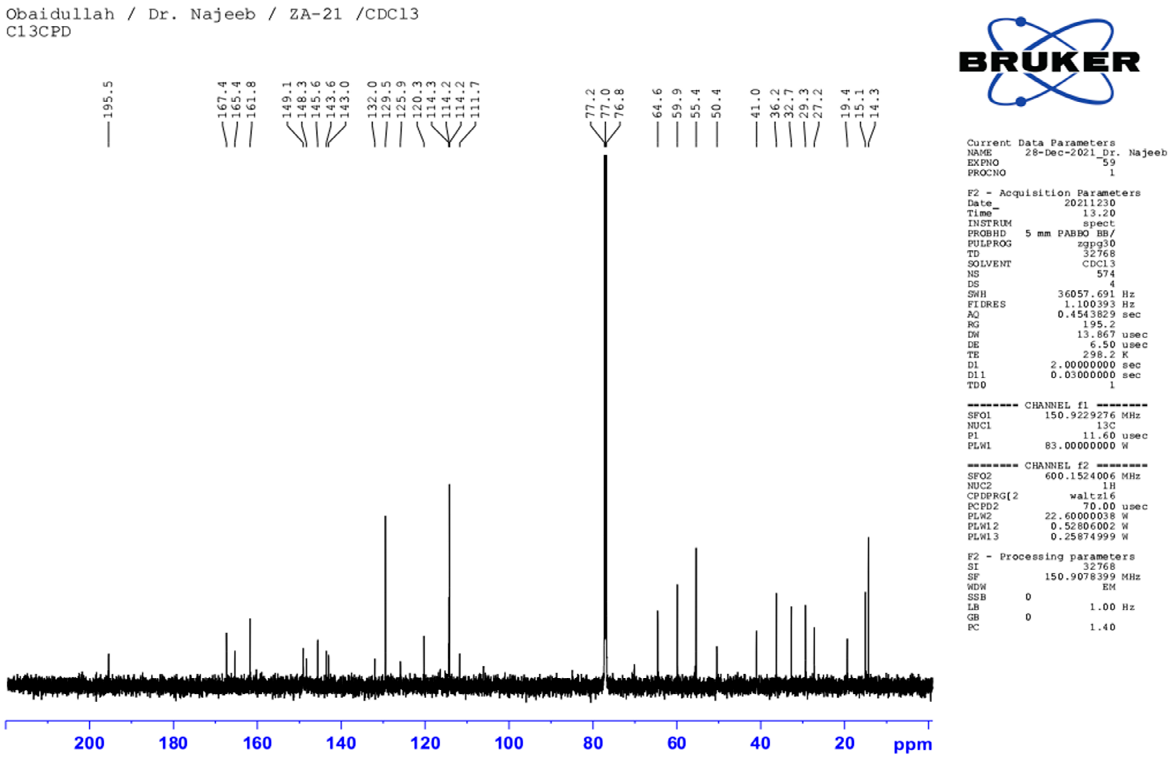

Figure S26: Mass, ^{1}H- and ^{13}C-NMR spectra of compound **24**

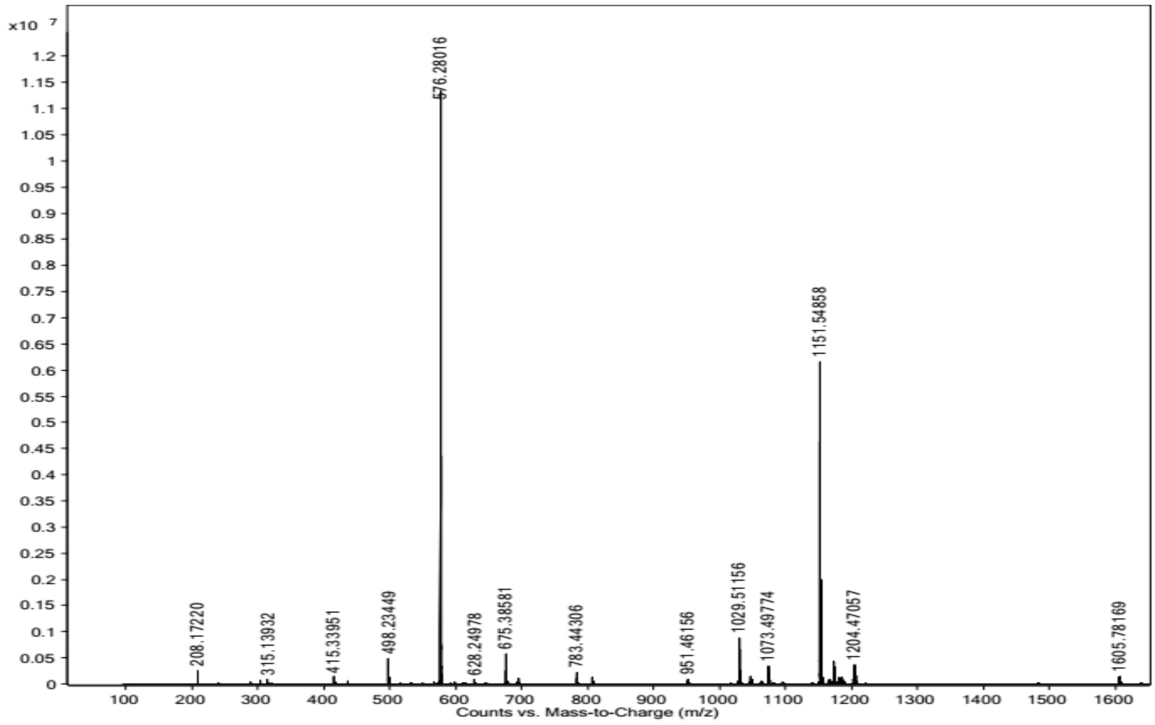

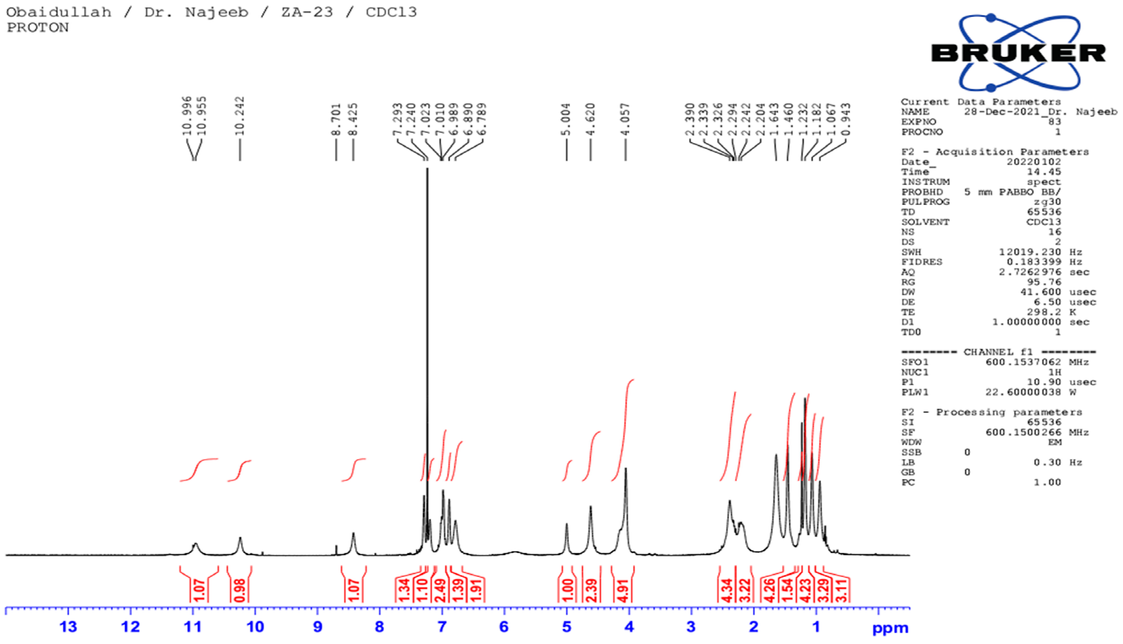

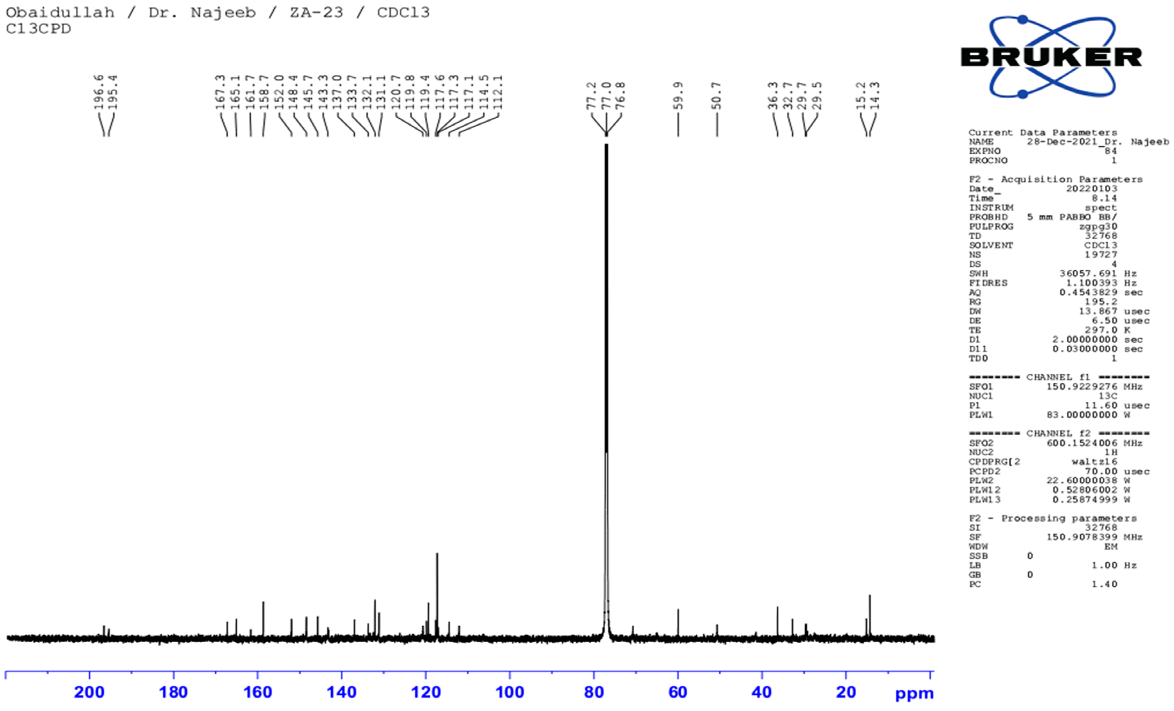

Figure S27: Mass, ^{1}H- and ^{13}C-NMR spectra of compound **25**

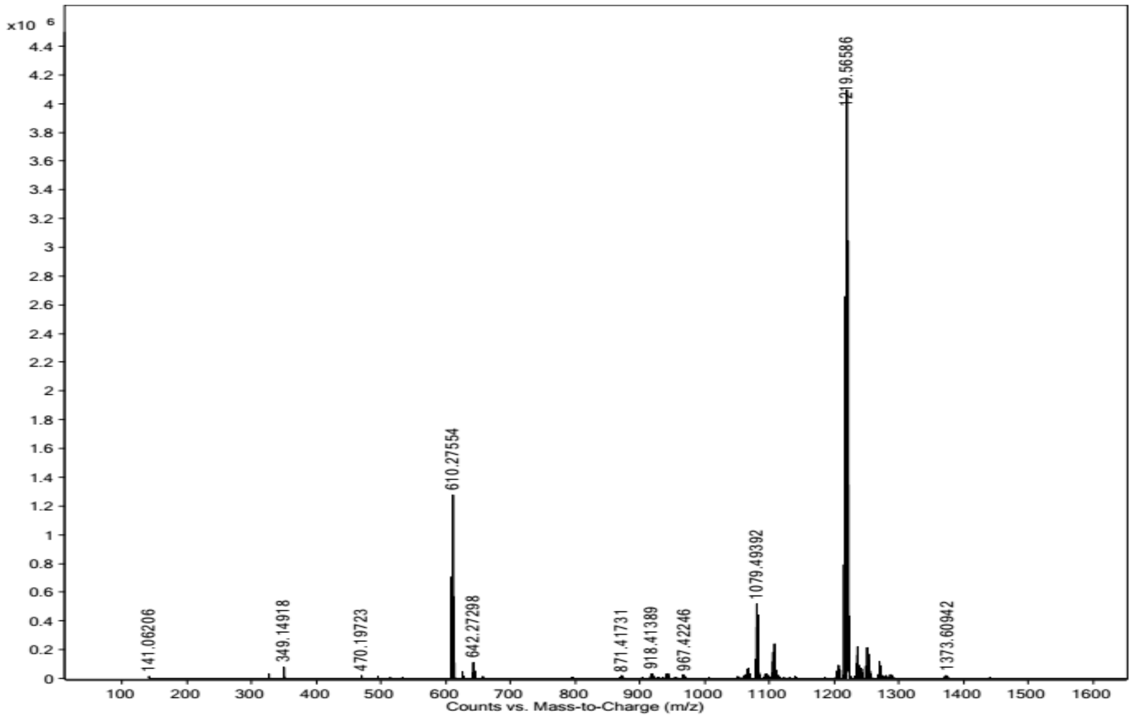
x10 6
141.06206
349.14918
470.19723
610.27554
642.27298
871.41731
918.41389
967.42246
1079.49392
1219.56586
1373.60942
Counts vs. Mass-to-Charge (m/z)

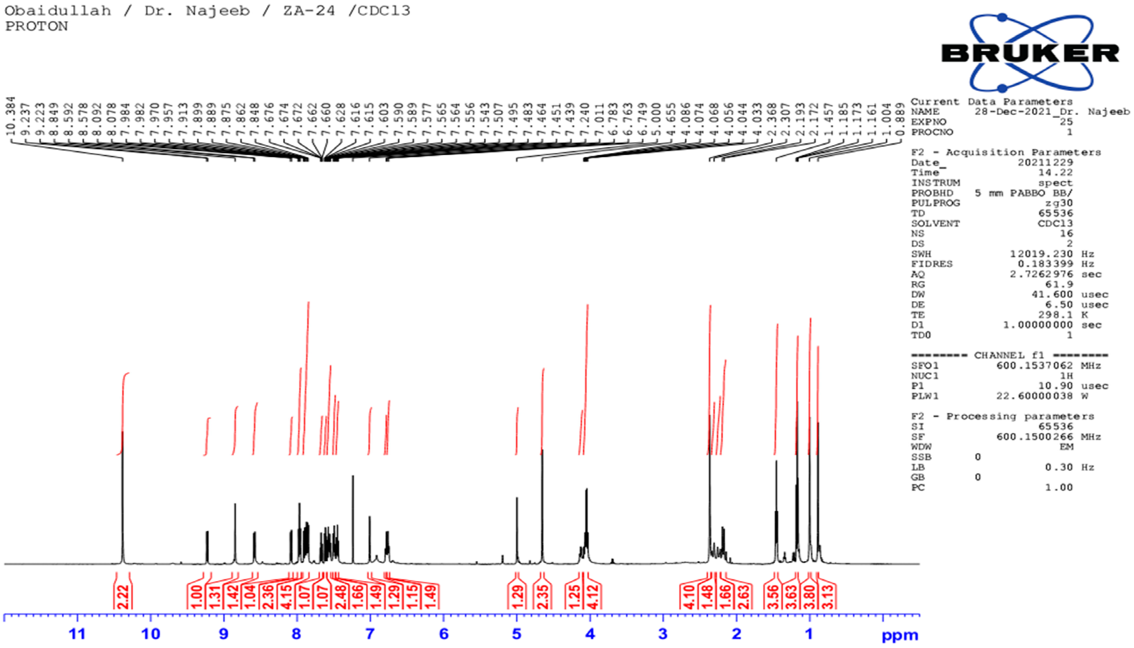
Obaidullah / Dr. Najeeb / ZA-24 /CDCl3
PROTON
BRUKER
Current Data Parameters
NAME 28-Dec-2021_Dr. Najeeb
EXPNO 25
PROCNO 1
F2 - Acquisition Parameters
Date_ 20211229
Time_ 14.22
INSTRUM spect
PROBHD 5 mm PABBO BB/
PULPROG zg30
TD 65536
SOLVENT CDCl3
NS 16
DS 2
SWH 12019.230 Hz
FIDRES 0.183399 Hz
AQ 2.7262976 sec
RG 61.9
DW 41.600 usec
DE 6.50 usec
TE 298.1 K
D1 1.00000000 sec
TD0 1
======== CHANNEL f1 ========
SFO1 600.1537062 MHz
NUC1 1H
P1 10.90 usec
PLW1 22.60000038 W
F2 - Processing parameters
SI 65536
SF 600.1500266 MHz
WDW EM
SSB 0
LB 0.30 Hz
GB 0
PC 1.00
ppm

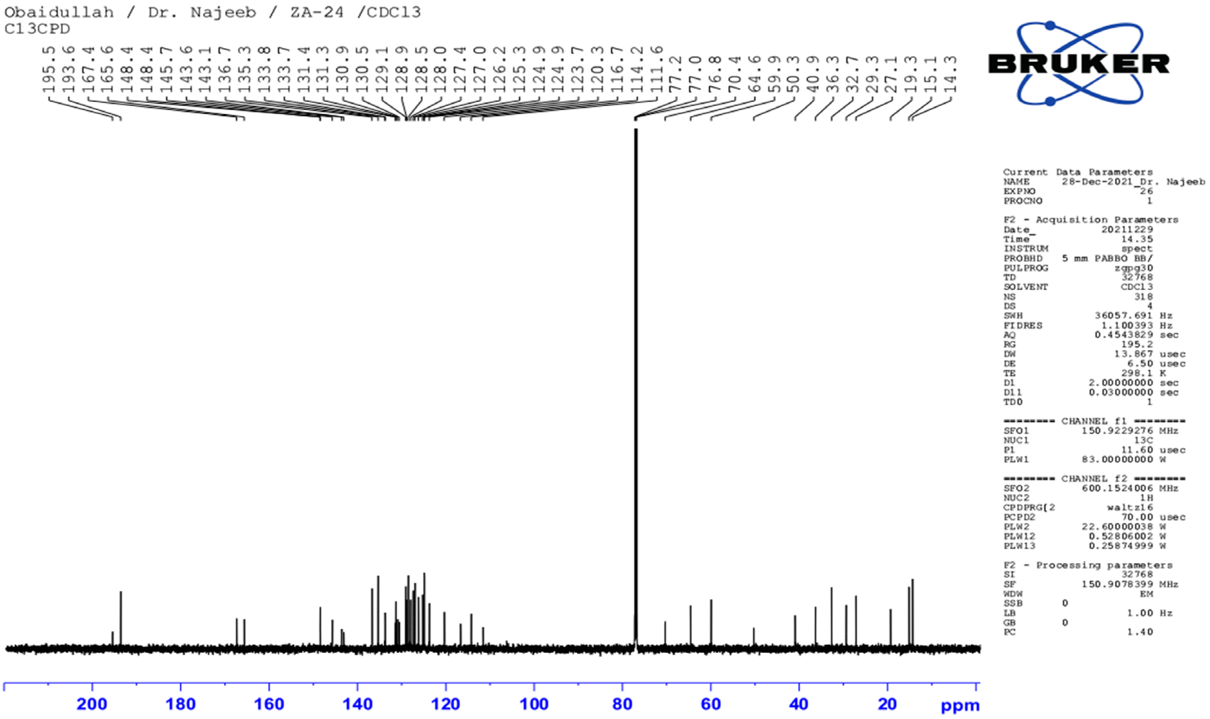

Figure S28: Mass, ^{1}H- and ^{13}C-NMR spectra of compound **26**

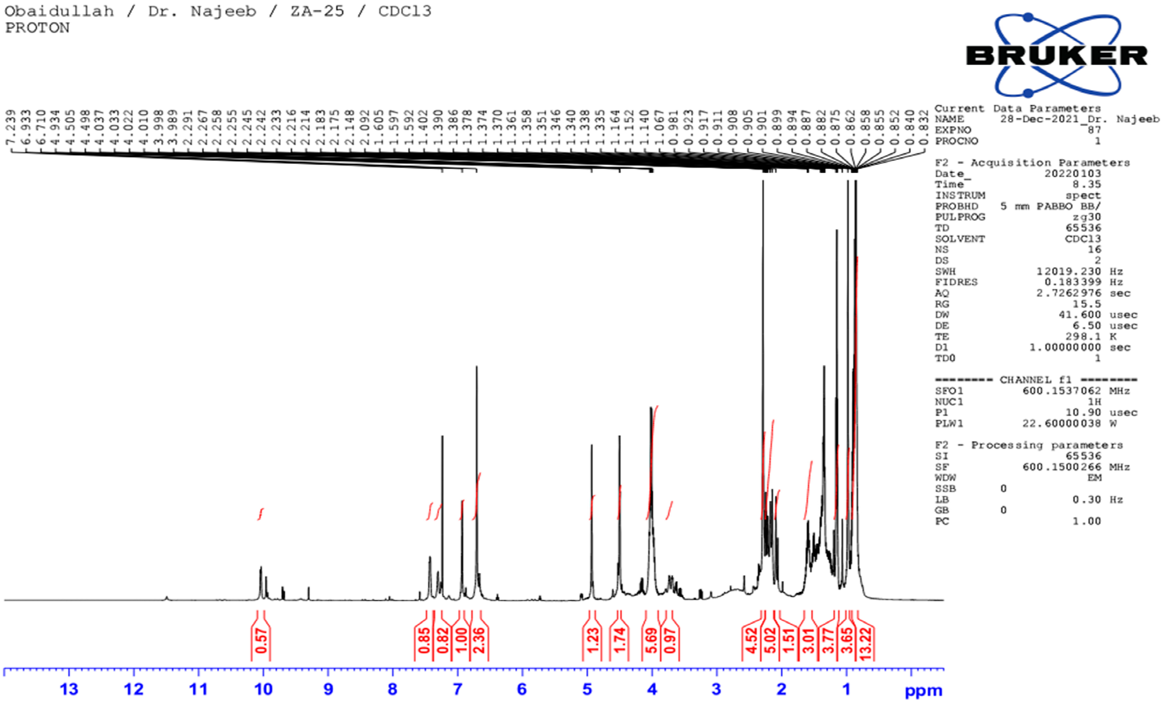

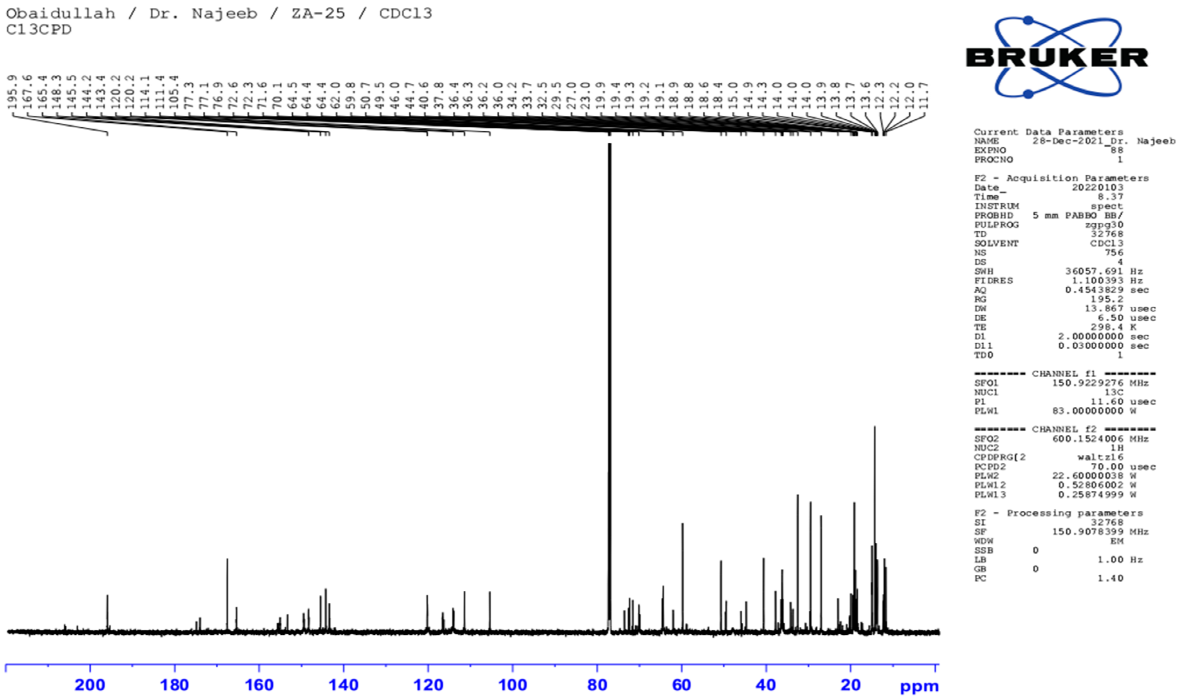

Figure S29. HR-ESI-MS, ^{1}H- and ^{13}C-NMR spectra of compounds **27**
